# A class of non-linear exposure-response models suitable for health impact assessment applicable to large cohort studies of ambient air pollution

**DOI:** 10.1007/s11869-016-0398-z

**Published:** 2016-03-02

**Authors:** Masoud M. Nasari, Mieczysław Szyszkowicz, Hong Chen, Daniel Crouse, Michelle C. Turner, Michael Jerrett, C. Arden Pope, Bryan Hubbell, Neal Fann, Aaron Cohen, Susan M. Gapstur, W. Ryan Diver, David Stieb, Mohammad H. Forouzanfar, Sun-Young Kim, Casey Olives, Daniel Krewski, Richard T. Burnett

**Affiliations:** 1 Environmental Health Science and Research Bureau, Health Canada, 200 Eglantine Driveway, Ottawa, Ontario K1A 0K9 Canada; 2 Public Health Ontario, Oakville, Ontario Canada; 3 McLaughlin Centre for Population Health Risk Assessment, Institute of Population Health, University of Ottawa, Ottawa, Ontario Canada; 4 Centre for Research in Environmental Epidemiology (CREAL), Madrid, Spain; 5 Universitat Pompeu Fabra (UPF), Barcelona, Spain; 6 CIBER Epidemiología y Salud Pública (CIBERESP), Barcelona, Spain; 7 Department of Environmental Health Sciences, University of California at Los Angeles, Los Angeles, CA USA; 8 Department of Economics, Brigham Young University, Provo, UT USA; 9 United States Environmental Protection Agency, Research Triangle Park, Durham, NC USA; 10 Health Effects Institute, Boston, MA USA; 11 Epidemiology Research Program, American Cancer Society, Atlanta, GA USA; 12 Institute of Health Metrics and Evaluation, Seattle, WA USA; 13 Institute of Health and Environment, Seoul National University, Seoul, South Korea; 14 Department of Environmental and Occupational Health Sciences, University of Washington, Seattle, WA USA

**Keywords:** Air pollution, Cohort, Exposure, Mortality, Particulate matter

## Abstract

The effectiveness of regulatory actions designed to improve air quality is often assessed by predicting changes in public health resulting from their implementation. Risk of premature mortality from long-term exposure to ambient air pollution is the single most important contributor to such assessments and is estimated from observational studies generally assuming a log-linear, no-threshold association between ambient concentrations and death. There has been only limited assessment of this assumption in part because of a lack of methods to estimate the shape of the exposure-response function in very large study populations. In this paper, we propose a new class of variable coefficient risk functions capable of capturing a variety of potentially non-linear associations which are suitable for health impact assessment. We construct the class by defining transformations of concentration as the product of either a linear or log-linear function of concentration multiplied by a logistic weighting function. These risk functions can be estimated using hazard regression survival models with currently available computer software and can accommodate large population-based cohorts which are increasingly being used for this purpose. We illustrate our modeling approach with two large cohort studies of long-term concentrations of ambient air pollution and mortality: the American Cancer Society Cancer Prevention Study II (CPS II) cohort and the Canadian Census Health and Environment Cohort (CanCHEC). We then estimate the number of deaths attributable to changes in fine particulate matter concentrations over the 2000 to 2010 time period in both Canada and the USA using both linear and non-linear hazard function models.

**Electronic supplementary material:**

The online version of this article (doi:10.1007/s11869-016-0398-z) contains supplementary material, which is available to authorized users.

## Introduction

Estimating the association between ambient concentrations of outdoor air pollution and mortality has traditionally been conducted with the use of cohort studies in which a group of subjects are identified, important mortality risk factors recorded, and the cohort is followed up for vital status and cause of death. Several cohort studies of ambient air pollution have been conducted in North America and Western Europe (Hoek et al., 2013). Although these studies have undergone extensive analyses, there has been little attention paid to the shape of the association between exposure and mortality.

Estimating the exposure-response relationships is critical to assessing the impact of specific regulatory actions to improve air quality on population mortality rates (Cohen et al., 2004, Ostro 2004, Lim et al., 2012; US EPA, 2012; Murray et al., 2015). Such analyses focus on predicting changes in the number of deaths associated with proposed or hypothesized changes in ambient air quality for specific populations. Change in death estimates such as

$$ \varDelta D={M}_0\left(1-\frac{1}{R\left(\beta, \varDelta z\right)}\right)\times \mathrm{pop} $$

can be calculated. Here, *ΔD* is the predicted change in the number of deaths for the population of interest, *M*_0_ is the baseline mortality rate, *Δz* is the predicted or observed change in ambient concentrations, and pop is the size of the target population. The mortality impact function, *R*(*β*, *Δz*), is often expressed as a relative risk function of a vector of unknown parameters *β*. Uncertainty is introduced into the analysis by simulation methods. Computer software is available to conduct such analyses (Global Burden of Disease (Lim et al., 2012, Murray et al., 2015), environmental Benefits Mapping Analysis Program (BenMAP) (US EPA 2015), World Health Organization (Ostro 2004), and Health Canada—Air Quality Benefits Assessment Tool—(Judek et al., 2012)).

The simplest form *R*(*β*, *Δz*) = *e* ^*β* × *Δz*^ for scalar *β* has been employed most often. The risk coefficient *β* is obtained from analyses of cohort studies almost exclusively based on the Cox proportional hazards model (Cox, 1972). In these models, a linear association between ambient concentration and the logarithm of the hazard rate, the instantaneous probability of death, is assumed.

Increasingly large study populations are now being used to examine the association between ambient concentrations of air pollution and adverse health outcomes. These studies link study specific data, population registries (Fisher et al., 2015), census information (Crouse et al., 2012, 2015; Hales et al., 2012; Cesaroni et al. 2013), or administrative health databases (Zeger et al., 2008; Greven et al. 2011; Carey et al., 2013) to vital status and cause of death over time and include hundreds of thousands to millions of deaths. Although these large sample sizes are attractive in terms of providing risk estimates with relatively small sampling errors, the suite of applicable analytical methods to characterize the exposure-response relation between air pollution and mortality is limited due to restrictions on the size of computer memory and analysis time.

Consequently, studies employing large cohorts often fit natural, restricted, or smoothing splines with a few degrees of freedom or a few categories of air pollution concentrations to describe the shape of the association between ambient concentrations and mortality because these functions can be estimated with standard computer software. Statistical tests are employed comparing these functions to linear in concentration models. These approaches require the selection of the number and placement of spline knots or categories of air pollution concentrations. They do not necessarily yield shapes that are suitable for health impact assessment, such as being monotonically non-decreasing. Smoothing splines are preferable in this regard in that they display less curvature but also may not be strictly monotonically increasing. Smoothing splines may also mask some detail of the shape of the concentration-response function, such as a threshold-type association, since air pollution typically explains only a small fraction of mortality, and as such the fitted smoothing spline often has little curvature. Smoothing splines also can pose computer implementation problems for very large cohorts. Unfortunately, no computer software is available to fit monotonic natural or smoothing splines for the Cox survival model, although monotonic smoothing splines have been implemented for the exponential family (Pya and Wood 2013). Finally, risk estimates from these non-parametric models are not as conveniently incorporated into current risk assessment software as are simple algebraic functions.

Due to these limitations, only very simple algebraic non-linear concentration-response functions have been examined. Krewski et al. (2009) and Crouse et al. (2012) used the logarithm of fine particulate matter (PM_2.5_) in their Cox survival models and showed that the log model was a superior predictor of mortality compared to models that included the untransformed concentration. Jerrett et al. (2009) fit a threshold function (i.e., no association below a fixed concentration and linear above) to the association between mortality from non-malignant respiratory disease and ground level ozone, again demonstrating a superior fit compared to the untransformed ozone concentration. These approaches to fitting algebraic risk functions are feasible since they are transformations of concentration and can be directly incorporated into the survival model structure required with standard software.

Non-linear concentration-mortality associations have been employed in the Global Burden of Disease 2004 project (Cohen et al., 2004). Here, the American Cancer Society Cancer Prevention Study II (CPS II) cohort was used to estimate the association between ambient fine particulate concentrations and mortality (Pope et al. 2002). A linear association was assumed from a counterfactual concentration of 7.5 to 30 μg/m^3^, the highest observed concentration at the time of any cohort study of PM_2.5_, with no additional risk assumed above this concentration. Sensitivity analyses were conducted assuming a linear association from the counterfactual to 50 μg/m^3^ and no additional risk above. A risk model based on the logarithm of concentration, whose risk parameter was estimated from the CPS II cohort, was also considered. These risk models were selected due to concerns that simple linear extrapolation of excess relative risk from the low concentrations observed in the USA, where the CPS II cohort was conducted, to much higher concentrations observed worldwide, would yield unreasonably large burden of disease estimates.

Burnett et al. (2014) suggest a more complex shape to describe the association between PM_2.5_ concentrations and mortality, with no association below some concentration, a near-linear association for low to moderate concentrations, and a diminishing change in risk as concentration increases over the global range of PM_2.5_. Using a meta-regression approach, Burnett et al. (2014) demonstrated that the PM_2.5_-mortality association was non-linear and more complex than could be described by a single unknown parameter such as that postulated by the logarithm of concentration. Burnett et al. (2014) incorporated information on risk from other sources of PM_2.5_ such as second-hand and active smoking and exposure to indoor sources of PM_2.5_ from the burning of biomass for cooking and heating. Concentrations from these sources are much larger than those observed in cohort studies of ambient air pollution that have been largely conducted in North America and Western Europe (Hoek et al., 2013). This information provided a means to estimate risk over the global range of ambient concentrations, the focus of their work.

These authors incorporated information from cohort studies of ambient air pollution by estimating study-specific risk based on contrasts in concentration from the study-specific mean to a counterfactual level. This non-linear risk model was used by the Global Burden of Disease 2010 project (Lim et al., 2012) to predict mortality burden for all 188 countries worldwide and has the form

$$ R\left(\beta, z\right)=\left\{\begin{array}{l}1\kern0.5em  if\kern0.5em z<{z}_{cf}\ \mathrm{otherwise}\\ {}1+{\beta}_1\times \left(1-{e}^{-{\beta}_2{\left(z-{z}_{cf}\right)}^{\beta_3}}\right)\end{array}\right\} $$

for counterfactual concentration *z*_*cf*_, below which no additional risk is assumed. Little power is available, however, to discriminate among shapes of the concentration-mortality association if only studies of ambient air pollution are used since their mean concentrations are similar. Thus additional information from other sources of fine particulate exposure was required to discern the shape of the concentration-mortality association. The unknown parameters in this model form cannot be estimated using standard survival model software and thus cannot be directly applied to the analysis of individual cohort studies.

As a result, there has been no consensus as to the shape of the concentration-mortality association solely based on information from existing cohort studies and no method has been suggested as to how to identify such shapes for use in health impact assessment. In this paper, we describe a modeling framework in which a class of flexible algebraic concentration-response functions can be fit to survival models using standard computer software and can accommodate very large cohorts. In addition, such models should ideally be able to be directly incorporated into existing health impact assessment computer software, both in terms of health impact predictions and their uncertainty. We illustrate our modeling approach with examples from the American Cancer Society Cancer Prevention Study II (CPS II) cohort and the Canadian Census Health and Environment cohort (CanCHEC).

### Relative risk model

In this section, we present a new class of concentration-response models that capture relationships between ambient concentrations and mortality in cohort studies which we a priori suggest are suitable for health impact assessment: linear, log-linear, threshold, and variations on sigmoidal shapes.

Consider the relative risk hazard model, *h*(*t*|*x*, *z*), of the form

$$ h\left(t\left|x,z\right.\right)={h}_o(t) \exp \left\{{\gamma}^{\prime }x+\beta *\omega \left(z\left|\mu, \tau \right.\right)*f(z)\right\}, $$

where *h*_*o*_(*t*) is the baseline hazard function of follow-up time *t*. Here, *f* is a known parametric monotonic function of air pollution concentration *z*, 0 < *ω*(*z*|*μ*, *τ*) < 1 is a known weighting function indexed by scalar values *μ* and *τ*, with *β* an unknown parameter to be estimated from the survival data using standard computer software. Here, *x* is a vector of known risk factors such as smoking history, diet, and education with corresponding unknown parameter vector *γ*. Our focus is on identifying the shape of the association between exposure and response and not on modeling the other risk factors. We a priori specify the risk factors in our analysis but for each model describing air pollution, we allow different estimates of *γ*.

Our model can be interpreted as a variable coefficient risk function where *β*(*z*) = *β* × *ω*(*z*|*μ*, *τ*) represents the risk coefficient that varies with concentration.

Since variations on a sigmoidal shape are of interest, we consider the logistic weighting function

$$ \omega \left(z\left|\mu, \tau \right.\right)={\left\{1+ \exp \left(-\left(\frac{z-\mu }{\tau \times r}\right)\right)\right\}}^{-1} $$

with *μ* a location parameter and *r* the range of *z*. The parameter *τ* controls the curvature of the weighting function. Larger values of *τ* produce shapes with less curvature. For example, when *τ* < 0.001 *ω* approximates an indicator function at *μ*. The weighting function is nearly linear for *τ* > 0.5.

We then consider two forms of *f*: *f*(*z*) = log(*z*) and *f*(*z*) = *z* that have been previously used to describe the relationship between outdoor air pollution exposure and mortality, where log(*z*) is the natural logarithm of concentration. We also consider values for *μ* selected based on percentiles of the distribution of *z*.

Concentration-response models that have been previously examined can be included within this framework. For example, the linear model can be specified by *f*(*z*) = *z* and setting *μ* to a large negative number such that *ω*(*z*|*μ*, *τ*) ~ 1, ∀ *z*. A similar specification can approximate the log-concentration model with *f*(*z*) = log(*z*). The threshold model is specified by *f*(*z*) = *z* − *T* with *ω*(*z*|*μ* = *T*, *τ* = 0.001) for threshold concentration *T*.

**Fig. 1:**
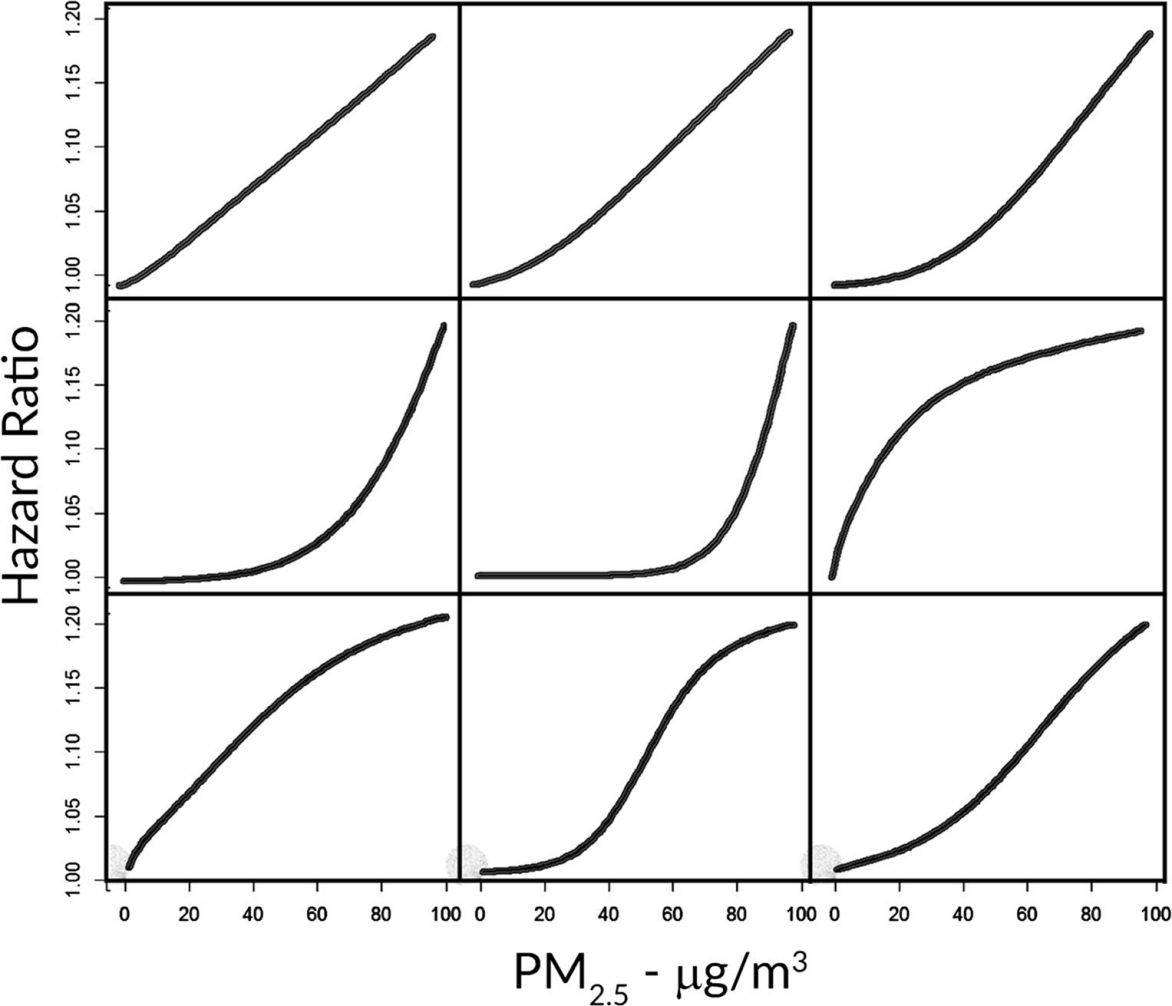
Selected hazard ratio forms

We have found in practice that for large variations in concentration our hazard function can have a marked curvature near *μ* and setting *τ* = 0.1 suitably reduces this undesirable curvature without dramatically changing the shape of the function. Functions that approximate powers of concentration can be constructed by setting *τ* = 0.2. Such power in concentration forms have been previously suggested for health impact functions (Burnett et al., 2014). Selected forms of the concentration-response function are displayed in Fig. 1 that indicate the variety of shapes that can be constructed from our model specification.

The unknown parameter *β* can be estimated using standard survival analysis software. The specific variable *ν*(*z*|*μ*) that best fits the data within our class is selected by the following procedure designed to minimize the number of model runs.

1. Create four weighting variables based on values of *μ* defined at the 0th, 25th, 50th, and 75th percentiles of the air pollution distribution with *τ* = 0.1 and an additional four variables with *τ* = 0.2. Multiply these eight weighting variables by the concentration or logarithm of concentration to create 16 variables. Run 16 Cox models based on these variables. Select the variable with the largest log-likelihood value among the 16 examined.
2. Given the best fitting *μ* value based on Step 1, fit two models setting *μ* to five percentile values greater than and less than the best fitting *μ*. For *μ* equal to the minimum concentration, subtract and increment equal to the difference between the 5th percentile and minimum concentration from the minimum concentration and denote this value as −5th percentile. Continue to take differences of minimum—10 % of increment and minimum—15 % of increment until log-likelihood is maximized.
3. If the log-likelihood values of the two models in Step 2 are not larger than the best fitting model in Step 1—STOP. Otherwise, run additional models with increments of *μ* set to five percentile values until the largest log-likelihood is achieved.

Computer code to conduct this search, written in both R and SAS by Hong Chen, is provided in the Electronic supplementary material.

### Incorporation into risk assessment models and uncertainty characterization

Suppose the current concentration is denoted by *z* ^*C*^ and we wish to predict the change in risk if a target population was exposed to concentrations predicted by future reduction scenarios, denoted by *z* ^*F*^. Then, the hazard ratio associated with such changes in concentration is

$$ \frac{\mathrm{HR}\left({z}^C\right)}{\mathrm{HR}\left({z}^F\right)}= \exp \left\{\varDelta \nu \left({z}^C,{z}^F\right)\times \widehat{\beta}\right\}, $$

where *Δν*(*z* ^*C*^, *z* ^*F*^) = *f*(*z* ^*C*^) × *ω*(*z* ^*C*^|*μ*) − *f*(*z* ^*F*^) × *ω*(*z* ^*F*^|*μ*) is the transformed change in air pollution. The change in the number of deaths associated with this change in exposure is calculated by

$$ \varDelta D={M}_0\left(1- \exp \left\{-\varDelta \nu \left({z}^C,{z}^F\right)\times \widehat{\beta}\right\}\right)\times \mathrm{pop}, $$

a form that can readily be incorporated into most health impact assessment software.

Uncertainty in estimates of *ΔD* is characterized by uncertainty of its components, namely *z*, *M*_0_, pop, and $\varDelta \nu \left({z}^C,{z}^F\right)\times \widehat{\beta}$. Uncertainty exists in $\varDelta \nu \left({z}^C,{z}^F\right)\times \widehat{\beta}$ from both uncertainty in the estimate $\widehat{\beta}$ for a specific variable definition *v*(*z*|*μ*) = *f*(*z*) × *ω*(*z*|*μ*) and the selection of the variable *v*(*z*|*μ*).

If *v*(*z*|*μ*) is assumed known, then

$$ \left.v\left(z\left|\mu \Big)\right.\times \beta \sim N\right(v\left(z\left|\mu \Big)\right.\times \widehat{\beta},v\right(z\left|\mu \Big)\right.\times s{e}_{\widehat{\beta}}\right), $$

with $s{e}_{\widehat{\beta}}$ the standard error of $\widehat{\beta}$ obtained from survival model software. Typical health impact assessment programs simulate a large number of realizations from this normal distribution resulting in an uncertainty distribution of excess deaths.

In our case, the form of *v*(*z*|*μ*) is not known a priori but has been determined from the data. One can incorporate the joint uncertainty in both *β* and *μ* by forming an “ensemble” model. Here, simulations of a large number of realizations of $v\left(z\left|\mu \right.\right)\times \widehat{\beta}$ weighted by the likelihood value for all the models fit in our model selection procedure are undertaken, as would be prescribed by Bayesian model averaging methods (Buckland et al., 1997).

### Illustrative examples

We illustrate the use of our model with an analysis of the association between estimates of ambient PM_2.5_ concentration and mortality in two large cohort studies: CPS II and CanCHEC. The analytic datasets used here are the same as that reported by Pope et al. (2015a) for CPS II and Crouse et al. (2015) for CanCHEC. We then compare the estimated number of excess deaths associated with changes in ambient concentrations between two time periods (~2000 and ~2010) for both the entire US and Canadian populations between our optimal or ensemble non-linear risk models for each cohort and the corresponding risk model that is linear in concentration.

### American Cancer Society Cancer Prevention Study II (CPS II) cohort

A total of 669,046 CPS II participants were assigned estimates of PM_2.5_ concentrations using a national-level hybrid land use regression and Bayesian Maximum Entropy interpolation model (Beckerman et al., 2013) for the 1998–2004 time period at their place of residence at the commencement of the study in 1982. Several mortality risk factors were included in the Cox survival model: education; marital status; body mass index (BMI); BMI squared; cigarette smoking status; cigarettes per day and cigarettes per day squared; years smoked and years smoked squared; started smoking at <18 years of age; passive smoking (hours); vegetable, fruit, and fiber and fat intake; beer, wine, and liquor consumption; occupational exposures; an occupational dirtiness index; and 1990 socio-demographic ecological covariates at both the ZIP code level and the ZIP code minus the county level mean (median household income; percentage of black residents, Hispanic residents, and percentage of adults with post-secondary education, unemployment, and poverty). The baseline hazard function was stratified by single year age groups, sex, and race.

**Fig. 2:**
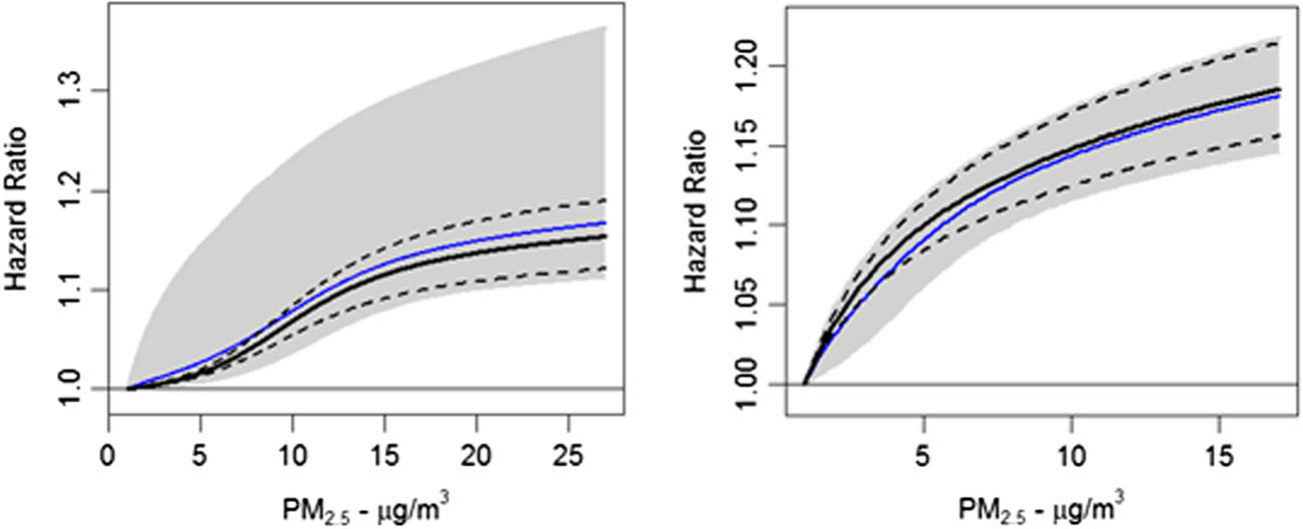
Hazard functions for CPS II (*left hand panel*) and CanCHEC (*right hand panel*). Optimal hazard function (*black solid line*) with uncertainty bounds (*dashed black lines*). Ensemble hazard function (*blue solid line*) with uncertainty bounds (*gray-shaded area*)

There were 237,201 deaths from all causes during the 1982–2004 follow-up period. [Note, we could not examine all non-accidental causes of death since we could not identify accidental causes prior to 1988 when subjects were linked to the computerized mortality files with specific causes of death.] Fine particulate concentrations ranged from 1.4 to 27.9 μg/m^3^. The best fitting “optimal” model was specified with *μ* given by the 5th percentile (8.2 μg/m^3^), *f*(*z*) = log(*z*), $\widehat{\beta}=0.0433$, and $s{e}_{\widehat{\beta}}=0.00446$ (Table 1). Similar results are presented for all models examined by our search algorithm and the ensemble likelihood-based weights assigned to each model. The optimal (black line) and ensemble (blue line) models are presented in Fig. 2 (left hand panel) in addition to their uncertainty intervals. The optimal and ensemble models are similar but the ensemble uncertainty interval is clearly wider than the corresponding interval for the optimal model. This is due to the non-trivial ensemble weights assigned to models with much larger estimates of *β* that correspond to smaller values of *μ* (Table 1). The linear in concentration model was $\widehat{\beta}=0.0071$ and $s{e}_{\widehat{\beta}}=0.00079$ with corresponding log-likelihood value −1,920,357.9. The log-likelihood value of the non-linear model (−1,920,350.7) was larger than that of the linear model suggesting the optimal non-linear model was an improved fit.

**Table 1:** Estimates of *β* and standard error by study (CPS II or CanCHEC) for non-linear models with *f*(*z*) = log(*z*) by value of *μ* and *τ*; likelihood weight used for ensemble estimates also presented

Study	μ μg/m^3^ (percentile)	*τ*	*β* (standard error)	Likelihood weight^a^
CPS II	−5.43 (−5 %)	0.1	0.0930 (0.00984)	0.036
	1.38 (0 %)	0.1	0.0802 (0.00843)	0.080
	8.19 (5 %)	0.1	0.0433 (0.00446)	0.460^b^
	9.04 (10 %)	0.1	0.0398 (0.00412)	0.324
	10.55 (25 %)	0.1	0.0351 (0.00369)	0.056
	1.38 (0 %)	0.2	0.0666 (0.00704)	0.044
CanCHEC	−4.10 (−10 %)	0.1	0.0620 (0.00469)	0.297
	−1.50 (−5 %)	0.1	0.0603 (0.00451)	0.363^b^
	1.10 (0 %)	0.1	0.0535 (0.00404)	0.329
	3.20 (5 %)	0.1	0.0399 (0.00307)	0.011

^a^All other models examined during our model search routine we assigned weights <0.001 and not reported

^b^Optimal model

### Canadian Census Health and Environment Cohort (CanCHEC)

CanCHEC has been described in detail previously (Crouse et al., 2012, 2015; Peters et al., 2013). It is a population-based cohort of 2.6 million subjects over 25 years of age who completed the 1991 census long-form. These subjects were linked to the Canadian Mortality Database using deterministic and probabilistic linkage methods from June 4, 1991 (census day) through December 31, 2006. For this illustrative analysis, we extracted all non-accidental deaths. Estimates of PM_2.5_ for the period 1998–2006 were obtained from a combination of satellite remote sensing information and a chemical transport model (van Donkelaar et al., 2013). We included in the Cox proportional hazards model covariates for visible minority status, marital status, highest level of education, immigrant status, employment status, aboriginal ancestry, occupational classification, and quintiles of household income (see Crouse et al., 2015 for details on the definitions of these variables). In addition to covariates recorded at the subject level, we calculated time-varying contextual variables from the closest census year (i.e., either 1991, 1996, 2001, or 2006). We stratified the baseline hazard by age (5 year groups) and sex.

The cohort experienced 328,585 non-accidental deaths during follow-up. Fine particulate concentrations ranged from 1.1 to 17.0 μg/m^3^. The optimal non-linear PM_2.5_ model was specified by *μ* = − 1.50, *f*(*z*) = log(*z*), $\widehat{\beta}=0.0603$, and $s{e}_{\widehat{\beta}}=0.00451$, with log-likelihood −3,196,246.5. This value of *μ* was determined by subtracting the difference between the 5th and 0th percentiles from the 0th percentile. Approximately 99 % of the likelihood based weights were assigned to the optimal model and models with adjacent values of *μ*, namely −1.5 and 3.7. The predicted hazard ratio for the optimal model (black solid line) and uncertainty bounds (black dashed line) are presented in Fig. 2, right hand panel. In addition, we present the ensemble hazard ratio of all models fit (blue solid line) and uncertainty bounds (gray-shaded area) in the right hand panel of Fig. 2. The optimal model hazard ratio is similar to the ensemble hazard ratio. However, the ensemble model uncertainty bounds are slightly larger than the optimal model bounds reflecting the additional uncertainty in the estimate of *μ*. Our estimate of the hazard function is clearly supra-linear in concentration and a better mortality predictor than the traditional linear in concentration model with $\widehat{\beta}=0.0080$, $s{e}_{\widehat{\beta}}=0.000644$, and log-likelihood −3,196,256.

## Estimating excess deaths associated with temporal changes in ambient PM_2.5_ concentrations

We have demonstrated that the optimal or best fitting hazard model within our class is non-linear for both the CPS II and CanCHEC cohorts (all causes of death for CPS II and non-accidental causes for CanCHEC) and a better predictor of mortality than a model which is linear in concentration. Of interest is how different these models are in predicting attributable deaths within the general population. We examined this issue using two datasets, one for Canada and the other for the USA.

The Canadian data consisted of estimates of ambient PM_2.5_ concentrations for each of 288 Census Divisions in Canada for two time periods: 1999–2001 and 2010–2012, in addition to the population over 25 and number of non-accidental deaths based on the 2010–2012 time period as complied by Stieb et al. (2015). The US data consisted of modeled estimates of ambient PM_2.5_ at the county level for the years 2000 and 2010 in addition to the population over age 30 and number of all cause of deaths for the year 2010. The modeled ambient PM_2.5_ concentration fields for the USA for 2000 and 2010 were provided by the Multiethnic Study of Atherosclerosis and Air Pollution (MESA-AIR) project team (Kim et al. 2015).

**Fig. 3:**
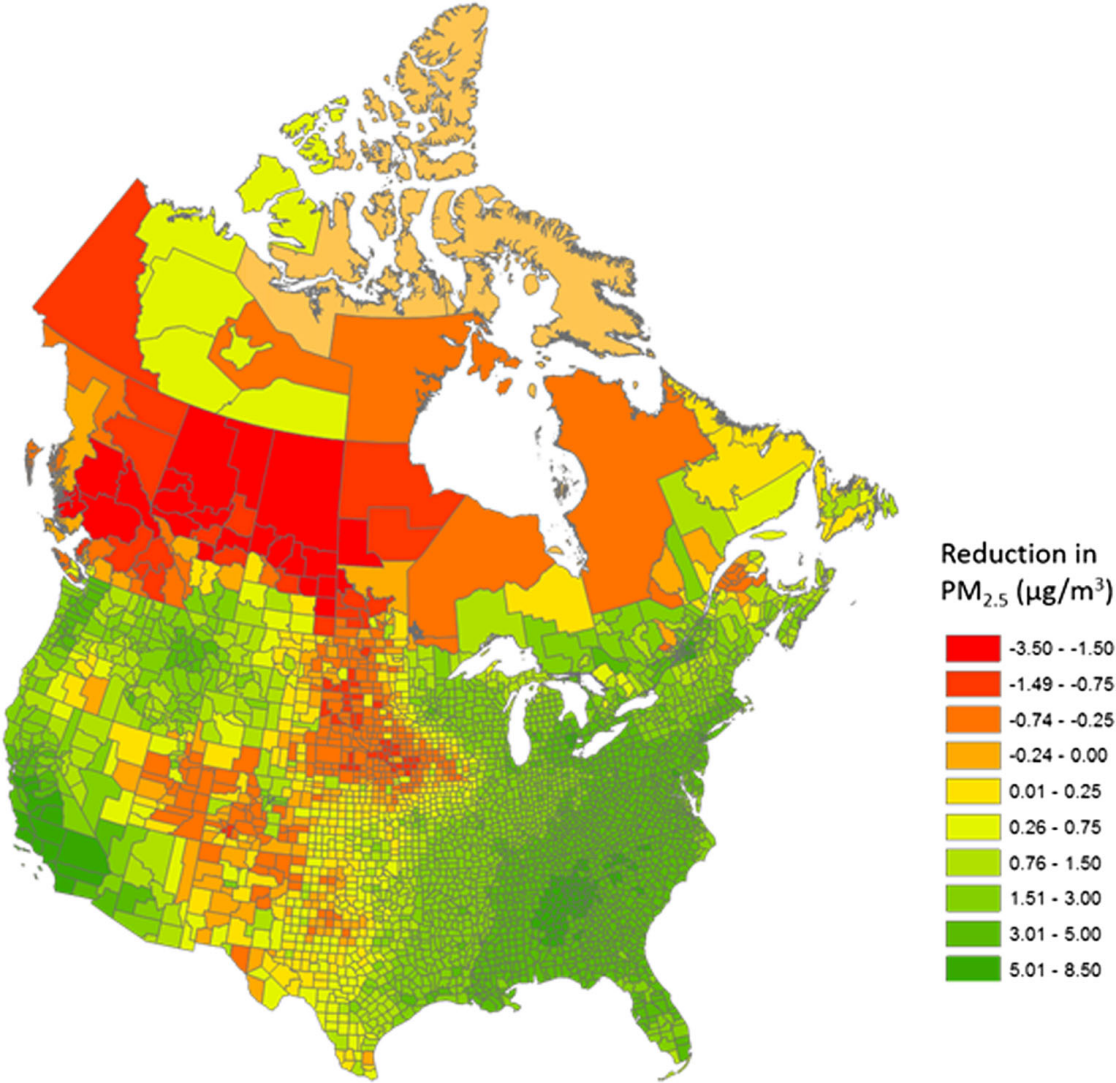
Change in PM_2.5_ concentrations over time. Census division are represented in Canada and counties in the USA. Time period displayed for Canada was based on 1999–2001 average and 2010–2012 average. Time period displayed for the USA was based on 2000 and 2010

PM_2.5_ air quality levels in much of North America improved between over time, with generally more widespread reductions in the Eastern and Northwestern USA and California and in the more southern census divisions in Canada (Fig. 3). The average change in concentration among census divisions in Canada was 1.1 μg/m^3^. However the population-weighted change was 2.0 μg/m^3^ or 24 % of 1999–2001 values based on a reduction from 8.5 μg/m^3^ in 1999–2001 to 6.5 μg/m^3^ in 2010–2012. The mean US county average concentration changed by 2.3 μg/m^3^ with the population-weighted concentration changing from 12.4 μg/m^3^ in 2000 to 8.7 μg/m^3^ in 2010, a decline of 3.7 μg/m^3^ or 17 % of 2000 concentrations. Greater changes in concentrations over time were observed for those areas with larger populations in both countries. Increases in PM_2.5_ occurred in 31 % of Canadian census divisions and 12 % of US counties, largely in the central region of the USA and the north and western regions of Canada, areas with sparser populations.

**Fig. 4:**
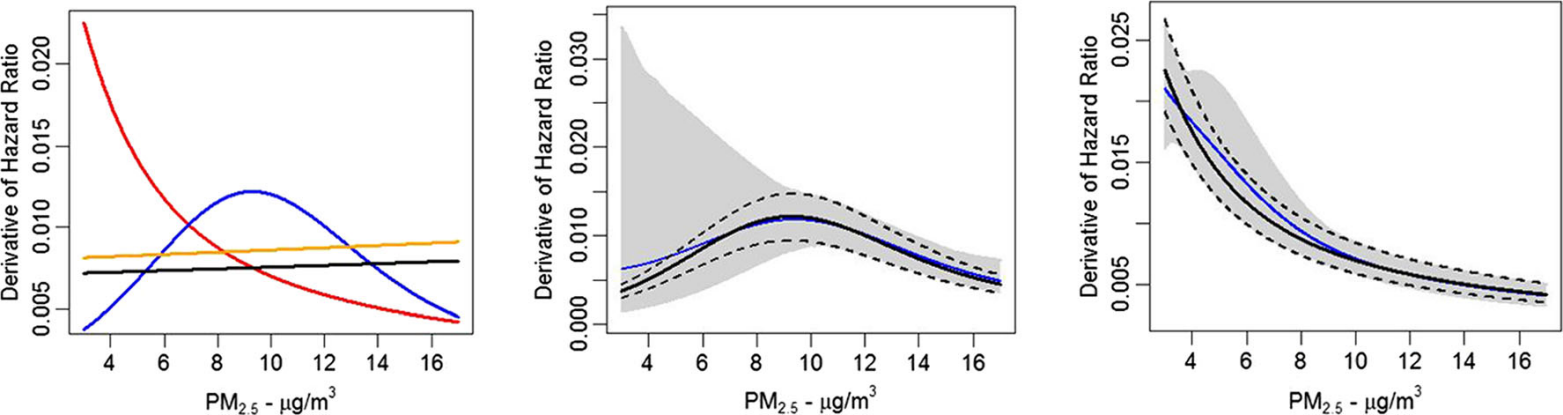
Derivative with respect to concentration of optimal non-linear models (*blue line* CPSII, *red line* CanCHEC) and linear in concentration models (*black line* CPSII, *orange line* CanCHEC) displayed in the *left hand panel*. Derivatives for CPS II (optimal model = *black line*, ensemble model = *blue line*) with uncertainty bounds (optimal model = *black dashed lines*, ensemble model = gray-shaded area) presented in the *middle panel* and CanCHEC (optimal model = *black line*, ensemble model = *blue line*) with uncertainty bounds (optimal model = *black dashed lines*, ensemble model = gray-shaded area) displayed in the *right hand panel*

The focus of our illustrative analysis is on changes in concentrations over time. As such, we are interested in how the hazard function changes with concentration. To understand this change, we plot the derivatives of the hazard functions (Fig. 4, left hand panel) with respect to concentration as suggested by Pope et al. 2015b. The CanCHEC hazard function derivative is greater than the CPS II derivative when PM_2.5_ <7 μg/m^3^. However, only 40 % of Canadians lived in areas below this level in 2000. The derivative of the CanCHEC optimal non-linear hazard function is greater than the derivative for the linear in concentration model when PM_2.5_ <9 μg/m^3^. Approximately half of Canadians lived in areas under his value in 2000. Estimates of deaths attributable to the difference in concentrations over time are presented in Table 2. Similar deaths were predicted for the linear in concentration (3477), optimal non-linear (3146), and ensemble non-linear (3323) CanCHEC models. This similarity is due to the fact that half of Canadians lived in regions where the non-linear model derivatives were greater/less than the linear model derivative. However, the excess deaths predicted by the CPS II linear in concentration model (3090) were smaller than the CPS II non-linear model (4302 for the optimal model and 4243 for the ensemble model) since more Canadians (60 %) lived in areas where the CPS II non-linear model derivative was larger than either the linear or non-linear model CanCHEC derivative.

**Table 2:** Estimates of excess deaths attributable to changes in PM_2.5_ concentration over time by form of hazard function (linear or non-linear), cohort (CanCHEC and CPS II), and country (Canada and USA)

Country (population weighted change in PM_2.5_)	Hazard model form—cohort	Number of excess deaths^a^ (95 % confidence interval)	Percent change in baseline mortality rate
Canada (2.0 μg/m^3^)	Linear—CanCHEC	3480 (2940–4020)	1.55
	Linear—CPS II	3090 (2430–3750)	1.38
	Non-linear optimal—CanCHEC	3146 (2700–3610)	1.41
	Non-linear ensemble—CanCHEC	3320 (2720–4060)	1.48
	Non-linear optimal—CPS II	4300 (3420–5200)	1.92
	Non-linear ensemble—CPS II	4240 (3100–5560)	1.90
	Combined non-linear ensemble^a^	3640 (2780–4500)	1.62
USA (3.7 μg/m^3^)	Linear—CanCHEC	68,700 (58,000–79,300)	2.82
	Linear—CPS II	60,900 (47,500–74,100)	2.50
	Non-linear optimal—CanCHEC	46,600 (39,700–53,400)	1.92
	Non-linear ensemble—CanCHEC	49,000 (40,700–57,100)	2.01
	Non-linear optimal—CPS II	77,700 (62,200–93,100)	3.20
	Non-linear ensemble—CPS II	76,700 (60,600–93,000)	3.15
	Combined non-linear ensemble^a^	61,900 (34,700–89,100)	2.54

^a^Meta-analytic combination of CanCHEC and CPS II ensemble models

Estimates of year 2000 and 2010 ambient PM_2.5_ concentrations along with the linear, optimal, and ensemble non-linear concentration-response hazard functions for the CanCHEC and CPS II cohorts were input into BenMAP to generate estimates of the number of deaths attributable to changes in exposure between 2000 and 2010 for the USA. The resulting estimates are presented in Table 2 for the CPS II-based linear model, the optimal and ensemble non-linear models based on CanCHEC, and both the optimal and ensemble non-linear models based on CPS II. For the USA, we observed that the CanCHEC model predicted fewer reductions in attributable deaths (46,600) compared to the linear model (60,900) and even fewer compared to the CPS II optimal (77,700) and ensemble (76,500) models. As with the Canadian data, these differences in attributable deaths are explained by the location in the exposure distribution where most of the change in concentration occurs. Only 5 % of the over 30 population in the USA lived in counties with 2000 concentrations less than 7 μg/m^3^, where the CanCHEC model derivative is greatest, while 61 % lived in counties with concentrations between 7 and 14 μg/m^3^, where the change in the CPS II model mortality response is greatest.

**Fig. 5:**
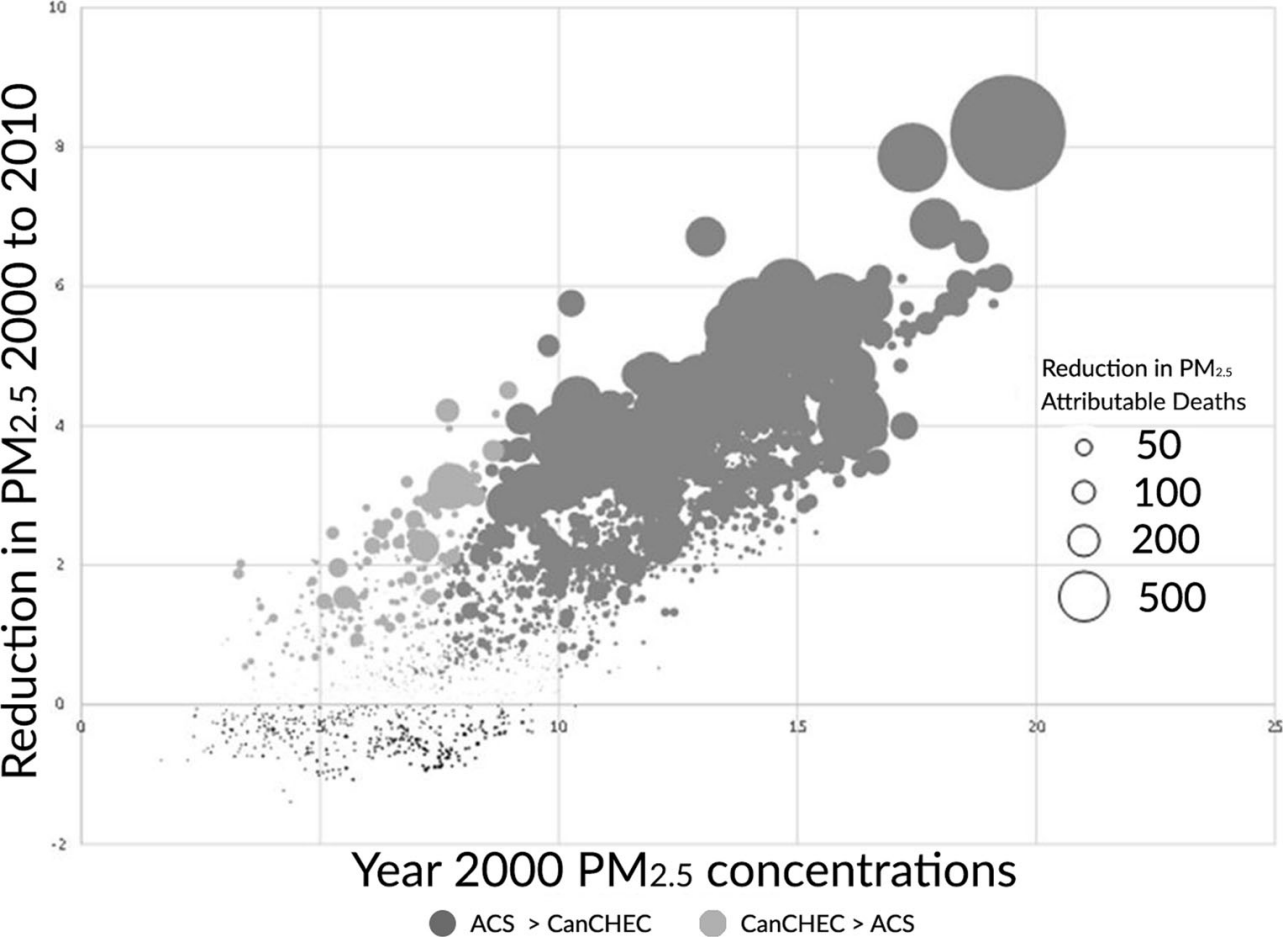
Estimated reductions in US premature deaths by combinations of 2000 PM_2.5_ concentration and PM_2.5_ change between 2000 and 2010

Figure 5 shows the distribution of county level estimated reduction in premature mortality for the USA by combinations of year 2000 PM_2.5_ concentrations and the change in PM_2.5_ between 2000 and 2010. Size of the circles is proportional to the predicted reductions in premature deaths for the CPS II optimal non-linear model (dark gray) or CanCHEC optimal non-linear model (light gray). The light gray circles indicate combinations of concentration levels and changes where the CanCHEC model predicts greater mortality impacts than the CPS II model. The dark gray circles indicate combinations of concentration levels and changes where the CPS II model predicts greater mortality impacts than the CanCHEC model. Black circles show the grid cells where there was an increase in PM_2.5_ between 2000 and 2010. The overall pattern of the distribution shows that the CPS II model predicts greater impacts in locations with greater concentrations and greater reductions, while the CanCHEC model gives greater impacts where concentrations are lower and reductions are smaller, consistent with the analysis of the model derivatives.

The incorporation of the additional uncertainty implied by alternative values of *μ* in estimates of excess deaths depends on the uncertainty in the derivative of the hazard function as shown in Fig. 4 for CPS II (middle panel) and CanCHEC (right hand panel) for both the optimal (dashed black lines) and ensemble (gray-shaded area) models. The uncertainty associated with the optimal and ensemble models was similar for both the CPS II and CanCHEC models when concentrations are greater than 8 μg/m^3^. However, the derivative of the ensemble models displayed much greater uncertainty for the CPS II model below this concentration and somewhat greater uncertainty for the CanCHEC model. This observation is consistent with our uncertainty estimates of excess deaths (Table 2) where in Canada, the ensemble models displayed greater uncertainty than the optimal models but no such pattern was observed in the USA due to the location of changes in concentration within the exposure distribution (at higher concentrations in the USA compared to Canada).

## Comparing and combining concentration-response functions

The forms of the concentration-mortality association identified in CanCHEC and CPS II are clearly different. The logarithm was selected for each cohort and the optimal value of *μ* was near the lower end of the exposure distribution for both cohorts (5th percentile for CPS II and the −5th percentile for CanCHEC). However, the rate of change for very low concentrations was greater in CanCHEC compared to CPS II and the opposite pattern was observed for medium and higher concentrations (Fig. 4).

This may be due to a few factors. The 5th percentile concentration for CPS II was 8.2 μg/m^3^ while that for CanCHEC was 3.2 μg/m^3^. This was due to both lower concentrations in Canada compared to the USA in general, and the fact that subjects in CanCHEC covered the entire population including those living in low exposure rural areas. There was, thus, additional uncertainty in the form of the function at lower concentrations for CPS II as evidenced by the need to include additional functions in the ensemble estimate with *μ* values (0th and −5th percentiles) lower than the optimal value at the 5th percentile.

However, the change in risk for concentrations larger than 9 μg/m^3^ was much greater for CPS II than that for CanCHEC (Fig. 4). This may be due to the form of the PM_2.5_ exposure model. The CPS II exposure model incorporated land use information including traffic counts while the CanCHEC exposure model used only remote sensing information. Turner et al. (2016) examined the effect of both regional and local variation in PM_2.5_ and mortality in CPS II cohort and found local variation, primarily induced by land use and traffic data, was a much stronger predictor than regional variation. The hazard ratio for a 10 μg/m^3^ change in PM_2.5_ based on regional variation was 1.05 (1.03, 1.07) while for local variation the hazard ratio was 1.27 (1.21, 1.34). This additional information may have improved the predictive power of the CPS II exposure model over the CanCHEC exposure model, especially in the center of the exposure distribution containing the majority of data.

We have presented two very different estimates of excess deaths attributable to changes in PM_2.5_ ambient concentrations over the first decade of this century (Table 2). We have suggested this could be due to both different concentration distributions and exposure models. Health impact assessments of PM_2.5_ have either used a single study, such as the US EPA (2012), or a meta-analysis of studies (Judek et al., 2012). Both these approaches use a linear in concentration risk model. The meta-analysis approach assumes a common, true, risk function and that each study is a random representation of that common function. In most cases, the risk function is characterized by a single parameter assumed to be normally distribution with a mean common to all studies and a study specific standard error. The meta-analysis approach uses a random effects model to estimate both a common mean and uncertainty as a function of true heterogeneity in risk among studies and within study error.

We can reduce the dimension of our ensemble estimates of risk for each study by first conducting the health impact assessment, which yields a single uncertainty distribution per study. We have found that the uncertainty distribution of excess deaths in our example is well approximated by a normal distribution. We can then pool the information between the two functions through the excess death distributions using the meta-analytic random effects procedure (Viechtbauer 2010). This approach yields mean estimates of excess deaths (Table 2) between the CanCHEC and CPS II estimates (61,900 for the USA and 3640 for Canada). However, since the two functions are very different, the uncertainty intervals are much wider than either function examined separately (Table 2).

## Discussion

We present an approach to characterizing the shape of the association between ambient concentrations of air pollution and mortality applicable to the analysis of large cohort studies and for use in health impact assessment. Our modeling approach is very simple to program and implement with standard computer software for survival analysis. The results can also be directly incorporated into existing health impact assessment software, including widely used software such as Health Canada’s AQBAT and the US EPA’s BenMAP. The computer code to implement our model identification and estimation procedure is provided in the Electronic supplementary material in both SAS and R.

Pope et al. (2015b) examine implications of using non-linear risk models for cost/benefit analysis. A feature of a linear model is that the magnitude of risk is proportional to the size of the change in exposure. However, for non-linear models, the location of exposure changes within the exposure distribution is also important (Pope et al. 2015b). The use of non-linear health impact assessment models makes the interpretation of such exposure changes more complex and a priori less predictable, as illustrated by our examples. We note, for example, that the linear in concentration model parameter was slightly greater for CanCHEC (0.008) than that for CPS II (0.007). However, in our examples for both Canada and the USA, the CPS II non-linear models predicted more deaths than did the CanCHEC non-linear models.

We suggest possible explanations for differences in the shape of the concentration-mortality function between CanCHEC and CPS II, namely the population size covered by low concentrations and the form of the exposure model. We suggest an approach to combining the distributions of excess deaths estimated from each model using standard meta-analysis methods to form a single summary uncertainty distribution. We also suggest that this approach is preferable to combining the non-linear functions themselves (Armstrong et al., 2014) and then conducting the health impact analysis since the focus of our analysis is on estimating disease burden associated with changes in exposure and not obtaining a common risk function. Our method combines both uncertainty in the risk function within each study and variation in the functions, as they pertain to burden estimates, between functions.

We present a new method to identify the shape of the association between air pollution and mortality in cohort study designs using the Cox proportional hazards model for analyses. However, our method is not restricted to the Cox survival model and can be used with any regression modeling technique. For example, the case-crossover design (Maclure 1991) is often used to examine the association between short-term exposure to air pollution and acute health events using a conditional logistic regression model. We provide computer code in both R and SAS to implement our search routine for such designs in the Electronic supplementary material.

## Electronic supplementary material

Below is the link to the electronic supplementary material.

ESM 1 (DOCX 121 kb)
