# Supplementary material for: A class of non-linear exposure-response models suitable for health impact assessment applicable to large cohort studies of ambient air pollution
Source: Air Qual Atmos Health. 2016 Mar 2;9(8):961–72. doi: 10.1007/s11869-016-0398-z (PMC5093184; doi:10.1007/s11869-016-0398-z)
Supplement: Supplementary file 1 — (DOCX 121 kb) [file 11869_2016_398_MOESM1_ESM.docx]

Supplementary Material

A Class of Non-Linear Exposure-Response Models Suitable for Health Impact Assessment Applicable to Large Cohort Studies of Ambient Air Pollution

Air Quality, Atmosphere & Health

Masoud M. Nasari, Mieczysław Szyszkowicz*, Hong Chen, Daniel Crouse, Michelle C. Turner, Michael Jerrett, C Arden Pope III, Bryan Hubbell, Neal Fann, Aaron Cohen, Susan M. Gapstur11, W. Ryan Diver, David Stieb, Mohammad H. Forouzanfar, Sun-Young Kim, Casey Olives, Daniel Krewski, Richard T. Burnett.

Dr. Mieczysław Szyszkowicz

Environmental Health Science and Research Bureau

Health Canada, Ottawa, Canada

200 Eglantine Driveway, Ottawa, ON

Ottawa, ON, K1A 0K9, Canada

Phone: (613) 762-1830

Fax: (613) 954-3768

Email:mietek.szyszkowicz@canada.ca

Should there be any question with this macro, please contact: Drs. Hong Chen (Hong.Chen@oahpp.ca) or Rick Burnett (Rick.Burnett@canada.ca)

Cox model

####################################SAS################################

************************************************************************

/*************************************************************************************************

*

* Nonlinear concentration-response function: model fitting and plotting procedure

*

* Date: Feb 24, 2016

* Version 2.10

*

* Purpose: This macro will fit a series of nonlinear concentration-response functions using

* cox proportional hazards models to identify optimal nonlinear relationship

*

* It will produce three outputs in the file directory specified by users:

*

* (1) a graph (.png) showing nonlinear relationship based on an optimal model, and if pre-specified, it will overlay an ensemble model,

* based on either all models examined or the best 3 models, according to their -2LogLik

* (2) a summary table (.pdf) listing coefficient, standard error, loglik, and function form of each cox model that was fitted

* (3) a summary table (.pdf) showing descriptive stats of air pollution exposure variable from the original and the trimmed datasets

*

* For more details about this modeling approach, please refer to the accompanying paper Masoud, Szyszkowicz,...,Burnett et al 2016 Air Quality, Atmosphere and Health

*

* Should there be any question with this macro, please contact:

* Drs Hong Chen (Hong.Chen@oahpp.ca) and Rick Burnett (Rick.Burnett@hc-sc.gc.ca)

*

*

* Please read the following notes before running the macro

*

* 1. All categorical variables should to be prepared as a series of dichotomous variables prior to calling macro

*

* 2. Exposure variable need to be define by using "fitvar" parameter

*

* 3. The following 3 parameters are optional: "strata", "label_exposure", and "label_unit"

*

* 4. To run time-fixed cox model, users need to specify "case" and "time" parameters,

* and omit "start" and "stop" parameters

*

* 5. To run time-varying cox model, users need to specify "case", "start", and "stop" parameters,

* and omit "time" parameter

*

* 6. If users do not wish to translate data, please specify translate=N or NO

*

* 7. By default, the plot shows the cr curve based on optimal model only.

* To overlay it with ensemble model, users need to specify "overlay" parameters.

* By defining overlay=all or overlay=ALL, users can overlay ensemble model based on all models examined.

* By defining overlay=any text string (except for "all" or "ALL"), users can overlay ensemble model based on 3 best models.

*

*

*

* To illustrate how to use this macro, 7 examples are given below:

*

* Example 1: (translate data, fit time-fixed cox models, with strata variable, only show optimal model)

*

* %fitap(datain=cohort_ABC,perc_trim=1,translate=yes,dataout=allmodels,case=status,time=time,fitvar=no2,covvars=age sex, strata=inst,

* label_exposure=NO2, label_unit=ppb, output_path=D:\Working directory\Cohort);

*

* Example 2: (translate data, fit time-fixed cox models, without strata variable, only show optimal model)

*

* %fitap(datain=cohort_ABC,perc_trim=1,translate=y,dataout=allmodels,case=status,time=time,fitvar=no2,covvars=age sex, strata=,

* label_exposure=NO2, output_path=D:\Working directory\Cohort);

*

* Example 3: (translate data, fit time-fixed cox models, with two strata variables, only show optimal model)

*

* %fitap(datain=cohort_ABC,perc_trim=5,translate=YES,dataout=allmodels,case=death,time=time,fitvar=pm25,covvars=ses,strata=age sex,

* label_exposure=pm25, label_unit=ug/m3, output_path=D:\Working directory\Cohort);

*

* Example 4: (translate data, fit time-varying cox models, with strata variable, only show optimal model)

*

* %fitap(datain=cohort_ABC,perc_trim=1,dataout=allmodels,case=status,start=T1,stop=T2,fitvar=pm25,covvars=sex ses,strata=age,

* label_exposure=pm25, label_unit=ug/m3, output_path=D:\Working directory\Cohort);

*

* Example 5: (no translate data, fit time-varying cox models, with strata variable, only show optimal model)

*

* %fitap(datain=cohort_ABC,perc_trim=1,translate=N,dataout=allmodels,case=Status,start=TStart,stop=TStop,fitvar=pm25,covvars=sex ses income bmi,strata=age,

* label_exposure=pm25, label_unit=ug/m3, output_path=D:\Working directory\Cohort);

*

* Example 6: (no translate data, fit time-varying cox models, with strata variable, overlay ensemble model based on all models examined)

*

* Note that to specify an ensemble model based on all models examined, please define overlay=all or overlay=ALL

*

* %fitap(datain=cohort_ABC,perc_trim=1,translate=no,dataout=allmodels,case=Status,start=TStart,stop=TStop,fitvar=pm25,covvars=sex ses income bmi,strata=age,

* label_exposure=pm25, label_unit=ug/m3, output_path=D:\Working directory\Cohort, overlay=all);

*

* Example 7: (no translate data, fit time-varying cox models, with strata variable, overlay ensemble model based on best 3 models examined)

*

* Note that to specify an ensemble model based on best 3 models examined, please define overlay=any text string, except for "all" or "ALL"

*

* %fitap(datain=cohort_ABC,perc_trim=1,translate=No,dataout=allmodels,case=Status,start=TStart,stop=TStop,fitvar=pm25,covvars=sex ses income bmi,strata=age,

* label_exposure=pm25, label_unit=ug/m3, output_path=D:\Working directory\Cohort, overlay=3);

*

*************************************************************************************************/

/**************************

* main function to select optimal model and ensemble model

***************************/

option LINESIZE=MAX;

%macro fitap(datain=,perc_trim=0,translate=yes,dataout=,case=,time=,start=,stop=,fitvar=,covvars=,strata=,label_exposure=,label_unit=,overlay=,output_path=);

/*Clean up any existing global macro variables*/

%symdel low_pct low_wc low_pct_1 low_wc_1 linear_model time start stop LL_min_all sum_LL_all LL_min_3 sum_LL_3;

/*Consider non-linear models first*/

%let linear_model = 0;

/*convert any conc < 1 to 1*/

data &datain.; set &datain.;

if &fitvar.<1 then &fitvar.=1;

run;

/*Trim data*/

%if 0<=&perc_trim.<= 10 %then %do;

%let trim_l=%SYSEVALF(&perc_trim.);

%let trim_r=%SYSEVALF(100-&perc_trim.);

%end;

%else %do; %put "WARNING: perc_trim must be an integer between 0 and 10"; %abort;%end;

proc univariate data=&datain. noprint;

var &fitvar.;

output out=percentiles pctlpts=&trim_l. &trim_r. pctlpre=ppp;

run;

data _null_;set percentiles;call symput('pctll',ppp%left(&trim_l.));call symput('pctlr',ppp%left(&trim_r.));run;

data aftertrim; set &datain.; where &pctll.<=&fitvar.<=&pctlr.; run;

/*Descriptive statistics of exp variable in the original and trimmed datasets*/

proc means data=&datain. n nmiss min q1 mean median q3 max;

var &fitvar.;

output out=stats_original n=total_obs nmiss=miss_obs min=min_ap q1=q1_ap mean=mean_ap median=median_ap q3=q3_ap max=max_ap;

run;

proc means data=aftertrim n nmiss min q1 mean median q3 max;

var &fitvar.;

output out=stats_aftertrim n=total_obs nmiss=miss_obs min=min_ap q1=q1_ap mean=mean_ap median=median_ap q3=q3_ap max=max_ap;

run;

data stats_original (drop=_type_ _freq_); set stats_original;

data_description="original dataset";

exp_var="&fitvar.";

run;

data stats_aftertrim (drop=_type_ _freq_); set stats_aftertrim;

data_description="trimmed dataset";

exp_var="&fitvar.";

run;

data overall_stat; set stats_original stats_aftertrim; run;

/*Translate data*/

%put "check translate";

%put &translate.;

%put &pctll.;

%put &pctlr.;

%if %upcase(&translate.)= YES or %upcase(&translate.)= Y %then %do;

%let Tran=1; /* translate z to have the min of 1 */

%end;

%else %do;

%let Tran=0; /* no translate */

%let pctll=0; /* no translate */

%end;

%put &Tran.;

%put &pctll.;

/*retain an original copy to be reused at each time when modelpct() is called*/

data aftertrim_backup; set aftertrim; run;

/*set tau=0.1*/

%let set_tau=0.1;

%let model_tau=1;

/* %LET tau=%SYSEVALF(&set_tau.*(&p100.-&p0.)); */

%LET count=0;

/*run 8 models to decide the model type and pct with smallest coefficient*/

%do mdltp=1 %TO 2;

%do pctt=0 %TO 75 %BY 25;

%modelpct(modeltype=&mdltp.,pct=&pctt.);

%end;%end;

data fourmodel_1;set mtll_:; run;

proc sort data=fourmodel_1;by WithCovariates;run;

data fourmodel_1;set fourmodel_1; if _N_=1;run;

data _null_;set fourmodel_1;

call symput('modeltp_a',modeltype); call symput('low_pct_a',pct); call symput('low_wc_a',WithCovariates);

run;

/*set tau=0.2*/

%let set_tau=0.2;

%let model_tau=2;

/* %LET tau=%SYSEVALF(&set_tau.*(&p100.-&p0.)); */

%LET count=0;

/*run 8 models to decide the model type and pct with smallest coefficient*/

%do mdltp=1 %TO 2;

%do pctt=0 %TO 75 %BY 25;

%modelpct(modeltype=&mdltp.,pct=&pctt.);

%end;%end;

data fourmodel_2;set mtll_:; run;

proc sort data=fourmodel_2;by WithCovariates;run;

data fourmodel_2;set fourmodel_2; if _N_=1;run;

data _null_;set fourmodel_2;

call symput('modeltp_b',modeltype); call symput('low_pct_b',pct); call symput('low_wc_b',WithCovariates);

run;

/*compare and find the optimal tau*/

%if &low_wc_a.<=low_wc_b. %then %do;

%let modeltp=&modeltp_a.;

%let low_pct=&low_pct_a.;

%let low_wc=&low_wc_a.;

%let set_tau=0.1;

/* %LET tau=%SYSEVALF(&set_tau.*(&p100.-&p0.)); */

%let model_tau=9;

%put &set_tau.;

%put &model_tau.;

/*retain rejected tau and related models*/

data dataout_reject; set dataout_2; call symput('set_tau_reject',0.2); run;

%end;

%else %do;

%let modeltp=&modeltp_b.;

%let low_pct=&low_pct_b.;

%let low_wc=&low_wc_b.;

%let set_tau=0.2;

/* %LET tau=%SYSEVALF(&set_tau.*(&p100.-&p0.)); */

%let model_tau=9;

%put &set_tau.;

%put &model_tau.;

/*retain rejected tau and related models*/

data dataout_reject; set dataout_1; call symput('set_tau_reject',0.1); run;

%end;

/*compare -5/+5 percentile around low_pct from above, which is either 0 or 25 or 50 or 75 */

%let low_wc_1=&low_wc.;

%let low_pct_1=&low_pct.;

%do pct_=(&low_pct.+5) %to (&low_pct.-5) %by -10;

%modelpct(modeltype=&modeltp., pct=&pct_.);

data _null_; set fits; call symput('new_wc',WithCovariates); call symput('new_pct',pct); run;

%if &new_wc.<&low_wc. %then %do; %let low_wc_1=&new_wc.; %let low_pct_1=&new_pct.; %end;

%end;

/* STOP if reaching mu=-15th, 100th, or LL is no longer smaller */

%do %while ( &low_pct_1. >= -10 and &low_pct_1. <= 95 and &low_pct_1. NE &low_pct.);

%let low_pct_temp = %SYSEVALF(&low_pct_1. + (&low_pct_1. - &low_pct.));

%let low_pct=&low_pct_1.;

%modelpct(modeltype=&modeltp., pct=&low_pct_temp.);

data _null_; set fits; call symput('new_wc',WithCovariates); call symput('new_pct',pct); run;

%if &new_wc.<&low_wc_1. %then %do; %let low_wc_1=&new_wc.; %let low_pct_1=&new_pct.; %end;

%end;

proc datasets;delete mtll: percentiles fourmodel: fits ;run;quit;

/*drop last run if mu was -20 or 105*/

data &dataout.; set &dataout.; where (pct NE -20); run;

data &dataout.; set &dataout.; where (pct NE 105); run;

/*add tau and append the rejected model outputs*/

proc sort data=&dataout.; by iteration; run;

data &dataout.; set &dataout.; tau=&set_tau.; run;

data dataout1_8; set &dataout.; where iteration<=8; run;

data dataout9_n; set &dataout.; where iteration>8; run;

data dataout9_n; set dataout9_n; iteration=iteration+8; run;

proc sort data=dataout_reject;by iteration;run;

data dataout_reject; set dataout_reject; tau=&set_tau_reject.; iteration=iteration+8; run;

data &dataout.; set dataout1_8 dataout_reject; run;

data &dataout.; set &dataout. dataout9_n; run;

/* print out chosen percentage and corresponding coefficient */

data &dataout.;set &dataout.;

rename pct=mu;

rename pctl=z_at_mu;

drop WithoutCovariates;

run;

proc sort data=&dataout.;by iteration;run;

/* Calculate ensemble weights using 3 models around the best fit, ie., based on best mu with +/- 5th%

The three model include last 2 models from the search + a 3rd model corresponding mu+5 of last model */

%symdel best_LL final_mu;

data &dataout. (drop=WithCovariates); set &dataout.; format LL d18.5; LL=WithCovariates; run;

data &dataout.; set &dataout.; rename LL=WithCovariates; run;

/* find optimal model corresonding to minimum LL */

proc sql noprint;

select min(WithCovariates) into: best_LL from &dataout.;

quit;

data &dataout.; set &dataout.; id=1; run;

data qaqc1; set &dataout.; run;

proc sort data=qaqc1; by WithCovariates; run;

data qaqc2; set qaqc1; id=_n_; run;

data qaqc2; set qaqc2; rename WithCovariates=best_LL2; where id=1; run;

data &dataout.; merge &dataout. (in=fro) qaqc2 (keep=id best_LL2); by id; if fro; run;

data &dataout.; set &dataout.;

if (WithCovariates = best_LL2) then do;

best3=1; call symput('final_mu',mu);

end;

run;

data &dataout.; set &dataout.; drop id best_LL2; run;

data &dataout.; set &dataout.; final_mu=&final_mu.; final_form=&modeltp.; run;

/* find 2 other alternative models */

proc sql noprint;

select sum(best3) into: count_best_LL from &dataout.; /* num of models with same smallest LL */

quit;

proc sql noprint;

select max(iteration) into: last_iteration from &dataout.; /* iteration id for last run */

quit;

%if &count_best_LL.=2 %then %do; /* a special case where last 2 runs had same smallest LL */

data &dataout.; set &dataout.; if mu=final_mu and tau=&set_tau. then call symput('best_final_LL_iteration',iteration); run;

%if &last_iteration. > &best_final_LL_iteration. %then %do; /* followed by additional run with a larger LL*/

data &dataout.; set &dataout.;

if iteration eq &last_iteration. then best3=3;

run;

%end;

%if &last_iteration. eq &best_final_LL_iteration. %then %do; /* followed by no more additional run*/

data &dataout.; set &dataout.;

if &new_pct. > &low_pct_1. and mu=(final_mu-10) and final_form=modeltype and tau=&set_tau. then best3=3; /* ascending*/

else if &new_pct. < &low_pct_1. and mu=(final_mu+10) and final_form=modeltype and tau=&set_tau. then best3=3; /* descending*/

run;

%end;

%end;

%if &count_best_LL.=1 %then

%do;

data &dataout.; set &dataout.;

if final_mu=-15 then do; /* a special case where last run reached mu=-15 */

if mu=-10 and tau=&set_tau. then best3=2;

if mu=-5 and tau=&set_tau. then best3=3;

end;

else if final_mu=100 then do; /* a special case where last run reached mu=100 */

if mu=95 and tau=&set_tau. then best3=2;

if mu=90 and tau=&set_tau. then best3=3;

end;

else do; /* all other cases */

if mu=(final_mu-5) and final_form=modeltype and tau=&set_tau. then best3=2;

if mu=(final_mu+5) and final_form=modeltype and tau=&set_tau. then best3=3;

end;

run;

%end;

/* derive LL from -2LL */

data &dataout.; set &dataout.;

LL = WithCovariates/(-2);

run;

/* calculate ensemble weights for all models */

proc sql noprint;

select min(LL) into: LL_min_all from &dataout.;

quit;

data &dataout.; set &dataout.;

LL_diff = exp(LL-&LL_min_all.);

run;

proc sql noprint;

select sum(LL_diff) into: sum_LL_all

from &dataout.;

quit;

data &dataout.; set &dataout.; wt=LL_diff/&sum_LL_all.;run;

data &dataout.; set &dataout.; drop LL_diff; run;

/* calculate ensemble weights for 3 final models */

data final3models; set &dataout.; where best3>=1; run;

proc sql noprint;

select min(LL) into: LL_min_3 from final3models;

quit;

data final3models; set final3models; LL_diff = exp(LL-&LL_min_3.); run;

proc sql noprint;

select sum(LL_diff) into: sum_LL_3 from final3models;

quit;

data &dataout.; set &dataout.;

if best3>=1 then LL_diff = exp(LL-&LL_min_3.);

else LL_diff=.;

run;

data &dataout.; set &dataout.; wt_final3=LL_diff/&sum_LL_3.;run;

data &dataout.; set &dataout.; if best3=. then wt_final3=.; drop LL_diff; run;

/* Bootstrap to sample beta based on joint model */

%if %length(&overlay.)>0 %then %do;

%simulate_z2(indata=&dataout., model="joint", over_lay=&overlay.);

proc sort data=newap3; by id; run;

%simulate_beta(indata=&dataout., model="joint", over_lay=&overlay.);

proc sort data=simdata; by id; run;

data newap3; merge newap3(in=fro) simdata; by id; if fro; run;

/* count max num of sim ap data points */

proc contents data=newap3 out=output_z; run;

proc sort data=output_z; by varnum; run;

data _null_;

set output_z;

call symputx("maximum",varnum-2);

run;

/* beta*transformed(z) for each simulated z data points */

data newap3 (drop=beta id); set newap3;

array CC{&maximum.} C1-C&maximum.;

do i=1 to &maximum.;

CC{i}=exp(beta*CC{i});

end;

run;

/* derive median and 2.5th% and 97th% */

%do i=1 %to &maximum.;

data newap_&i.; set newap3;

keep C&i.;

run;

proc univariate data=newap_&i. noprint;

var C&i.;

output out=beta_distr_&i. pctlpts= 2.5 50 97.5 pctlpre=P;

run;

data beta_distr_&i.;set beta_distr_&i.; id=&i.; run;

%if &i.=1 %then %do;

data beta_distr; set beta_distr_&i.; run;

%end;

%else %do;

data beta_distr; set beta_distr beta_distr_&i.; run; /* contain 4 variables: id, p2.5, p50, p97.5 */

%end;

%end;

data newap4(keep = z_sim);

do i = &pctll. to &pctlr. by 0.1;

z_sim = i;

output;

end;

run;

data newap4; set newap4; id=_n_; run;

proc sort data=newap4; by id; run;

proc sort data=beta_distr; by id; run;

data beta_distr; merge beta_distr(in=fro) newap4; if fro; by id; run;

%if %upcase(&translate.)= YES or %upcase(&translate.)= Y %then %do;

%if &modeltp.=1 %then %do;

data newap_joint; set beta_distr;

rename p50=rr_mean;

rename p97_5=rr_ucl;

rename p2_5=rr_lcl;

ap = z_sim;

run;

%end;

%else %if &modeltp.=2 %then %do;

data newap_joint; set beta_distr;

rename p50=rr_mean;

rename p97_5=rr_ucl;

rename p2_5=rr_lcl;

ap = z_sim;

run;

%end;

%end;

%else %do;

data newap_joint; set beta_distr;

rename p50=rr_mean;

rename p97_5=rr_ucl;

rename p2_5=rr_lcl;

ap = z_sim;

run;

%end;

%end;

/* Bootstrap to sample beta based on optimal model */

%simulate_z2(indata=&dataout., model="optimal");

proc sort data=newap3; by id; run;

%simulate_beta(indata=&dataout., model="optimal");

proc sort data=simdata; by id; run;

data newap3; merge newap3(in=fro) simdata; by id; if fro; run;

/* count max num of sim ap data points */

proc contents data=newap3 out=output_z; run;

proc sort data=output_z; by varnum; run;

data _null_;

set output_z;

call symputx("maximum",varnum-2);

run;

/* beta*transformed(z) for each simulated z data points */

data newap3 (drop=beta id); set newap3;

array CC{&maximum.} C1-C&maximum.;

do i=1 to &maximum.;

CC{i}=exp(beta*CC{i});

end;

run;

/* derive median and 2.5th% and 97th% */

%do i=1 %to &maximum.;

data newap_&i.; set newap3;

keep C&i.;

run;

proc univariate data=newap_&i. noprint;

var C&i.;

output out=beta_distr_&i. pctlpts= 2.5 50 97.5 pctlpre=P;

run;

data beta_distr_&i.;set beta_distr_&i.; id=&i.; run;

%if &i=1 %then %do;

data beta_distr; set beta_distr_&i.; run;

%end;

%else %do;

data beta_distr; set beta_distr beta_distr_&i.; run; /* contain 4 variables: id, p2.5, p50, p97.5 */

%end;

%end;

data newap4(keep = z_sim);

do i = &pctll. to &pctlr. by 0.1;

z_sim = i;

output;

end;

run;

data newap4; set newap4; id=_n_; run;

proc sort data=newap4; by id; run;

proc sort data=beta_distr; by id; run;

data beta_distr; merge beta_distr(in=fro) newap4; if fro; by id; run;

%if %upcase(&translate.)= YES or %upcase(&translate.)= Y %then %do;

%if &modeltp.=1 %then %do;

data newap_optimal; set beta_distr;

rename p50=rr_mean;

rename p97_5=rr_ucl;

rename p2_5=rr_lcl;

ap = z_sim;

run;

%end;

%else %if &modeltp.=2 %then %do;

data newap_optimal; set beta_distr;

rename p50=rr_mean;

rename p97_5=rr_ucl;

rename p2_5=rr_lcl;

ap = z_sim;

run;

%end;

%end;

%else %do;

data newap_optimal; set beta_distr;

rename p50=rr_mean;

rename p97_5=rr_ucl;

rename p2_5=rr_lcl;

ap = z_sim;

run;

%end;

/* plot joint and optimal models */

%plot_cr(indata_joint=newap_joint, indata_optimal=newap_optimal, expo=&label_exposure., unit=&label_unit., over_lay=&overlay.);

/* add a pure linear model with z */

%let linear_model = 1;

%put &linear_model.;

%put &modeltp.;

%put &low_pct_1.;

%modelpct(modeltype=&modeltp., pct=&low_pct_1.);

/* add a pure log(z) model */

%let linear_model = 2;

proc datasets;delete fits ;run;quit;

%modelpct(modeltype=&modeltp., pct=&low_pct_1.);

/* print description of original/trimmed datasets and model fitting, and clean up working directory */

ods pdf file="&output_path.\model_fitting_summary.pdf";

title;

proc print data=overall_stat;

title "Summary statistics of air pollution exposure variable in the original and trimmed datasets";

run;

title;

title;

data &dataout.;set &dataout. (drop=LL);

length model_function $15.;

if modeltype=1 then model_function='z*logit';

else if modeltype=2 then model_function='log(z)*logit';

else if modeltype=3 then model_function='pure linear';

else if modeltype=4 then model_function='pure log';

rename WithCovariates=LL;

drop WithoutCovariates pct pctl best3 final_form final_mu modeltype;

run;

/* proc sort data=&dataout.;by iteration;run; */

data &dataout.; set &dataout.; drop iteration Criterion; run; /* simplif summary table so as to fit in one page */

data &dataout.; set &dataout.; rename mu=location; run;

data &dataout.; set &dataout.; rename z_at_mu=mu; rename wt_final3=final_wt; run;

/* data &dataout.; set &dataout.; tau=&set_tau.; run; */

data prt; set &dataout.; run;

proc print data=prt;

title "Summary description of model fitting";

run;

title;

ods pdf close;

/* export output table to csv format */

proc export data=&dataout.

outfile="&output_path.\model_fitting_summary.csv"

dbms=csv

replace;

run;

/* clean up non-essential datasets */

proc datasets library=work;

delete sample: sample_all beta_distr final3models

newap newap_joint newap_optimal param prt overall_stat simdata temp_sim temp_sim2 qaqc: percentiles output_z

Z_sim_distr new_datain mtll: fits combined Beta_distr: dataout: newap: ;

run;quit;

%Mend fitap;

/**************************

* fit cox PH model with z

**************************/

%macro modelpct(modeltype=,pct=);

/*translate ap data to have the min of 1 or 0, depending on log or linear model*/

/*note that this only applies to translate=Y*/

/*note that if translate=N, then &pctll.=0 and &Tran.=0*/

/*for log model: translate dataset to have the min of 1*/

%if &modeltype.=2 %then %do;

data aftertrim; set aftertrim_backup; z=&fitvar.-&pctll.+&Tran.; run;

%end;

/*for linear model: translate dataset to have the min of 0*/

%else %if &modeltype.=1 %then %do;

data aftertrim; set aftertrim_backup; z=&fitvar.-&pctll.; run;

%end;

proc sql noprint;

select max(z) into: p100 from aftertrim;

select min(z) into: p0 from aftertrim;

quit;

proc univariate data=aftertrim noprint;

var z;

output out=percentiles pctlpts= 5 to 95 by 5 pctlpre=P;

run;

data percentiles; set percentiles; p_5=&p0.-(p5-&p0.); run; /* add a variable p_5 denoting 5 pctl below p0*/

data percentiles; set percentiles; p_10=&p0.-(p10-&p0.); run; /* add a variable p_10 denoting 10 pctl below p0*/

data percentiles; set percentiles; p_15=&p0.-(p15-&p0.); run; /* add a variable p_15 denoting 15 pctl below p0*/

proc transpose data=percentiles out=percentiles;run;

data _null_;set percentiles;

call symput(_name_,col1);

run;

/* calc tau based on set_tau */

%LET tau=%SYSEVALF(&set_tau.*(&p100.-&p0.));

/*calc log transformed ap data*/

%if &pct.= -5 %then %do;

%let mu=&p_5.; /* if 5 percentile less than p0, then change to _5 */

data aftertrim; set aftertrim;

%if &modeltype.=1 %then %do;

APvar=(z)*(1/(1+exp(-(z-&mu.)/&tau.)));

%end;

%if &modeltype.=2 %then %do;

APvar= log(z)*(1/(1+exp(-(z-&mu.)/&tau.)));

%end;run;

%end;

%else %if &pct. = -10 %then %do;

%let mu=&p_10.; /* if 10 percentile less than p0, then change to _10 */

data aftertrim; set aftertrim;

%if &modeltype.=1 %then %do;

APvar=(z)*(1/(1+exp(-(z-&mu.)/&tau.)));

%end;

%if &modeltype.=2 %then %do;

APvar= log(z)*(1/(1+exp(-(z-&mu.)/&tau.)));

%end;run;

%end;

%else %if &pct. < -10 %then %do;

%let mu=&p_15.; /* if 15 or more percentile less than p0, then change to _15 */

data aftertrim; set aftertrim;

%if &modeltype.=1 %then %do;

APvar=(z)*(1/(1+exp(-(z-&mu.)/&tau.)));

%end;

%if &modeltype.=2 %then %do;

APvar= log(z)*(1/(1+exp(-(z-&mu.)/&tau.)));

%end;run;

%end;

%else %do;

%let mu=&&p&pct.;

data aftertrim; set aftertrim;

%if &modeltype.=1 %then %do;

APvar=(z)*(1/(1+exp(-(z-&mu.)/&tau.)));

%end;

%if &modeltype.=2 %then %do;

APvar= log(z)*(1/(1+exp(-(z-&mu.)/&tau.)));

%end;run;

%end;

/* non-linear model*/

%if &linear_model. eq 0 %then %do;

%if %length(&start.)=0 %then %do;

%if %length(&time.)=0 %then %do; %put "WARNING: TIME variable is missing"; %abort;%end;

%if %length(&strata.)=0 %then %do;

proc phreg data=aftertrim;

model &time.*&case.(0)=APvar &covvars.;

ods output FitStatistics=fits(where=(Criterion='-2 LOG L'))

ParameterEstimates=param (where=(parameter='APvar'));

run;

%end;

%else %do;

proc phreg data=aftertrim;

model &time.*&case.(0)=APvar &covvars.;

strata &strata.;

ods output FitStatistics=fits(where=(Criterion='-2 LOG L'))

ParameterEstimates=param (where=(parameter='APvar'));

run;

%end;

%end;

%if %length(&start.)>0 %then %do;

%if %length(&stop.)=0 %then %do; %put "WARNING: STOP variable is missing"; %abort;%end;

%if %length(&strata.)=0 %then %do;

proc phreg data=aftertrim;

model (&start.,&stop.)*&case.(0)=APvar &covvars.;

ods output FitStatistics=fits(where=(Criterion='-2 LOG L'))

ParameterEstimates=param (where=(parameter='APvar'));

run;

%end;

%else %do;

proc phreg data=aftertrim;

model (&start.,&stop.)*&case.(0)=APvar &covvars.;

strata &strata.;

ods output FitStatistics=fits(where=(Criterion='-2 LOG L'))

ParameterEstimates=param (where=(parameter='APvar'));

run;

%end;

%end;

%end;

/* pure linear z model */

%if &linear_model. eq 1 %then %do;

%if %length(&start.)=0 %then %do;

%if %length(&time.)=0 %then %do; %put "WARNING: TIME variable is missing"; %abort;%end;

%if %length(&strata.)=0 %then %do;

proc phreg data=aftertrim;

model &time.*&case.(0)=z &covvars.;

ods output FitStatistics=fits(where=(Criterion='-2 LOG L'))

ParameterEstimates=param (where=(parameter='z'));

run;

%end;

%else %do;

proc phreg data=aftertrim;

model &time.*&case.(0)=z &covvars.;

strata &strata.;

ods output FitStatistics=fits(where=(Criterion='-2 LOG L'))

ParameterEstimates=param (where=(parameter='z'));

run;

%end;

%end;

%if %length(&start.)>0 %then %do;

%if %length(&stop.)=0 %then %do; %put "WARNING: STOP variable is missing"; %abort;%end;

%if %length(&strata.)=0 %then %do;

proc phreg data=aftertrim;

model (&start.,&stop.)*&case.(0)=z &covvars.;

ods output FitStatistics=fits(where=(Criterion='-2 LOG L'))

ParameterEstimates=param (where=(parameter='z'));

run;

%end;

%else %do;

proc phreg data=aftertrim;

model (&start.,&stop.)*&case.(0)=z &covvars.;

strata &strata.;

ods output FitStatistics=fits(where=(Criterion='-2 LOG L'))

ParameterEstimates=param (where=(parameter='z'));

run;

%end;

%end;

%end;

/* pure log(z) model */

%if &linear_model. eq 2 %then %do;

data new_datain; set aftertrim; ap_new=log(z); run;

%if %length(&start.)=0 %then %do;

%if %length(&time.)=0 %then %do; %put "WARNING: TIME variable is missing"; %abort;%end;

%if %length(&strata.)=0 %then %do;

proc phreg data=new_datain;

model &time.*&case.(0)=ap_new &covvars.;

ods output FitStatistics=fits(where=(Criterion='-2 LOG L'))

ParameterEstimates=param (where=(parameter='ap_new'));

run;

%end;

%else %do;

proc phreg data=new_datain;

model &time.*&case.(0)=ap_new &covvars.;

strata &strata.;

ods output FitStatistics=fits(where=(Criterion='-2 LOG L'))

ParameterEstimates=param (where=(parameter='ap_new'));

run;

%end;

%end;

%if %length(&start.)>0 %then %do;

%if %length(&stop.)=0 %then %do; %put "WARNING: STOP variable is missing"; %abort;%end;

%if %length(&strata.)=0 %then %do;

proc phreg data=new_datain;

model (&start.,&stop.)*&case.(0)=ap_new &covvars.;

ods output FitStatistics=fits(where=(Criterion='-2 LOG L'))

ParameterEstimates=param (where=(parameter='ap_new'));

run;

%end;

%else %do;

proc phreg data=new_datain;

model (&start.,&stop.)*&case.(0)=ap_new &covvars.;

strata &strata.;

ods output FitStatistics=fits(where=(Criterion='-2 LOG L'))

ParameterEstimates=param (where=(parameter='ap_new'));

run;

%end;

%end;

%end;

data param; set param; call symput('new_coef',Estimate); call symput('new_std',StdErr);run;

%if &linear_model. eq 0 %then %do;

data fits;set fits; length modeltype $4.; modeltype=&modeltype.; pct=&pct.; pctl=&mu.; coef=&new_coef.;

stderr=&new_std.;

run;

/*data fits;set fits; if modeltype=1 then pctl=pctl-&Tran.; run;*/

%end;

%if &linear_model. eq 1 %then %do;

data fits;set fits; length modeltype $4.; modeltype=3; pct=.; pctl=.; coef=&new_coef.; stderr=&new_std.; run;

%end;

%if &linear_model. eq 2 %then %do;

data fits;set fits; length modeltype $4.; modeltype=4; pct=.; pctl=.; coef=&new_coef.; stderr=&new_std.; run;

%end;

/* first 8 model runs corresponding to tau=0.1 */

%if &model_tau.=1 %then %do;

%LET count=%SYSEVALF(&count. + 1);

data fits;set fits; iteration=&count.; run;

/* the very first model examined */

%if &count.=1 %then %do;

data dataout_1;set fits;run; %put "lastly here here here"; %put &count.; %end;

%else %do;

data dataout_1;set dataout_1 fits; run; %end;

%if &pct.<0 %then %do; %let abs_pct=%sysfunc(abs(&pct.)); data mtll_&modeltype._&abs_pct._;set fits; run;; %end;

%else %do; data mtll_&modeltype._&pct.;set fits; run; %end;

%end;

/* second 8 model runs corresponding to tau=0.2 */

%else %if &model_tau.=2 %then %do;

%LET count=%SYSEVALF(&count. + 1);

data fits;set fits; iteration=&count.; run;

/* the very first model examined */

%if &count.=1 %then %do;

data dataout_2;set fits;run; %put "lastly here here here"; %put &count.; %end;

%else %do;

data dataout_2;set dataout_2 fits; run; %end;

%if &pct.<0 %then %do; %let abs_pct=%sysfunc(abs(&pct.)); data mtll_&modeltype._&abs_pct._;set fits; run;; %end;

%else %do; data mtll_&modeltype._&pct.;set fits; run; %end;

%end;

/* continue the rest of model runs, based on selected tau, either 0.1 or 0.2 */

%else %if &model_tau.=9 %then %do;

%LET count=%SYSEVALF(&count. + 1);

data fits;set fits; iteration=&count.; run;

/* based on the selected tau, initiate output dataset*/

%if &count.=9 %then %do;

%if &set_tau.=0.1 %then %do;

data &dataout.;set dataout_1;run;

%end;

%else %if &set_tau.=0.2 %then %do;

data &dataout.;set dataout_2;run;

%end;

%end;

/* append the 9th model and onwards*/

%put "Append the 9th model and onwards";

data &dataout.;set &dataout. fits; run;

%if &pct.<0 %then %do; %let abs_pct=%sysfunc(abs(&pct.)); data mtll_&modeltype._&abs_pct._;set fits; run; %end;

%else %do; data mtll_&modeltype._&pct.;set fits; run; %end;

%end;

run;

%Mend modelpct;

/**************************

* Simulate z over 1000 realizations

**************************/

%macro simulate_z1(modeltype=, nn=);

/* based on range of trimmed and translated ap_data */

%if %upcase(&translate.)= YES or %upcase(&translate.)= Y %then %do;

%let max_ap = %SYSEVALF(&pctlr.-&pctll.+&Tran.);

data newap(keep = z_sim);

do i = &Tran. to &max_ap. by 0.1;

z_sim = i;

output;

end;

run;

%end;

%else %do;

data newap(keep = z_sim);

do i = &pctll. to &pctlr. by 0.1;

z_sim = i;

output;

end;

run;

%end;

/* if linear model, have the min of 0 */

%if &modeltype.=1 %then %do;

data newap; set newap;

z_sim=z_sim-&Tran.;

run;

%end;

/* prepare nn x _n_ matrix to store z_sim */

proc sort data=newap; by z_sim; run;

data newap; set newap; seqno = _n_ ; run;

proc transpose data=newap out=newap_wide prefix=C; /* col name is C1, C2, ..., Cxxx */

id seqno;

var z_sim;

run;

data newap2 (drop=_name_ i); set newap_wide; /* nn*num_z matrix */

do i=1 to &nn.;

output;

end;

run;

/* extract percentiles of z_sim */

proc sql noprint;

select max(z_sim) into: z_p100 from newap;

select min(z_sim) into: z_p0 from newap;

quit;

proc univariate data=newap noprint;

var z_sim;

output out=percentiles pctlpts= 0 to 100 by 5 pctlpre=z_p;

run;

data percentiles; set percentiles; z_p_5=&z_p0.-(z_p5-&z_p0.); run; /* add a variable z_p_5 denoting 5 pctl below z_p0*/

data percentiles; set percentiles; z_p_10=&z_p0.-(z_p10-&z_p0.); run; /* add a variable z_p_10 denoting 10 pctl below z_p0*/

data percentiles; set percentiles; z_p_15=&z_p0.-(z_p15-&z_p0.); run; /* add a variable z_p_15 denoting 15 pctl below z_p0*/

proc transpose data=percentiles out=percentiles;run;

%mend simulate_z1;

%macro simulate_z2(indata=, model=, over_lay=);

/* prepare z for use in simulation, varying depending on perc, set_tau, and funcform */

%if &model.="joint" %then %do;

/* all models examined */

%if %length(&over_lay.)>0 %then %do;

%if %upcase(&over_lay.)=ALL %then %do;

data temp_sim; set &indata.; run;

data temp_sim; set temp_sim; seqno = _n_; wt_final3=wt; run;

%end;

%else %if %upcase(&over_lay.) ne ALL %then %do;

data temp_sim; set &indata.; where best3>=1; run;

data temp_sim; set temp_sim; seqno = _n_; run;

%end;

proc sql noprint;

select max(seqno) into: num_models from temp_sim; /* total number of models examined */

quit;

%let counter=1;

%do k=1 %to &num_models.;

data _null_; set temp_sim;

call symput('wt_each',wt_final3); call symput('z_tau',tau);

call symput('z_mu',mu);call symput('mtype',modeltype);

where seqno=&k.;

run;

/* num of models */

%let N=%SYSEVALF(1000*&wt_each., floor);

%if &N.> 0 %then %do;

/* simulate ap data */

%simulate_z1(modeltype=&mtype., nn=&N.);

data _null_;set percentiles;

call symput(_name_,col1);

run;

proc sql noprint;

select max(seqno) into: num_z from newap; /* total number of z_sim data points */

quit;

%let xxx=&num_z.;

/* calc tau based on set_tau of each model run */

%LET z_tau_val=%SYSEVALF(&z_tau.*(&z_p100.-&z_p0.));

/*calc log transformed ap data*/

%if &z_mu.= -5 %then %do;

%let z_mu_val=&z_p_5.; /* if 5 percentile less than p0, then change to _5 */

data newap2; set newap2;

array CC{&xxx.} C1-C&xxx.;

%do i=1 %to &xxx.;

%if &mtype.=1 %then %do;

CC{&i.}=(CC{&i.})*(1/(1+exp(-(CC{&i.}-&z_mu_val.)/&z_tau_val.)));

%end;

%if &mtype.=2 %then %do;

CC{&i.}= log(CC{&i.})*(1/(1+exp(-(CC{&i.}-&z_mu_val.)/&z_tau_val.)));

%end;

%end;

run;

%end;

%else %if &z_mu. = -10 %then %do;

%let z_mu_val=&z_p_10.; /* if 10 percentile less than p0, then change to _10 */

data newap2; set newap2;

array CC{&xxx.} C1-C&xxx.;

%do i=1 %to &xxx.;

%if &mtype.=1 %then %do;

CC{&i.}=(CC{&i.})*(1/(1+exp(-(CC{&i.}-&z_mu_val.)/&z_tau_val.)));

%end;

%if &mtype.=2 %then %do;

CC{&i.}= log(CC{&i.})*(1/(1+exp(-(CC{&i.}-&z_mu_val.)/&z_tau_val.)));

%end;

%end;

run;

%end;

%else %if &z_mu. < -10 %then %do;

%let z_mu_val=&z_p_15.; /* if 15 or more percentile less than p0, then change to _15 */

data newap2; set newap2;

array CC{&xxx.} C1-C&xxx.;

%do i=1 %to &xxx.;

%if &mtype.=1 %then %do;

CC{&i.}=(CC{&i.})*(1/(1+exp(-(CC{&i.}-&z_mu_val.)/&z_tau_val.)));

%end;

%if &mtype.=2 %then %do;

CC{&i.}= log(CC{&i.})*(1/(1+exp(-(CC{&i.}-&z_mu_val.)/&z_tau_val.)));

%end;

%end;

run;

%end;

%else %do;

%let xx=&z_mu.;

%let z_mu_val=&&z_p&xx.;

%put &z_mu_val.;

data newap2; set newap2;

array CC{&xxx.} C1-C&xxx.;

%do i=1 %to &xxx.;

%if &mtype.=1 %then %do;

CC{&i.}=(CC{&i.})*(1/(1+exp(-(CC{&i.}-&z_mu_val.)/&z_tau_val.)));

%end;

%if &mtype.=2 %then %do;

CC{&i.}= log(CC{&i.})*(1/(1+exp(-(CC{&i.}-&z_mu_val.)/&z_tau_val.)));

%end;

%end;

run;

%end;

%if &counter.=1 %then %do;

data newap3; set newap2; run;

%end;

%else %do;

data newap3; set newap3 newap2; run;

%end;

%let counter=%SYSEVALF(&counter.+1);

%end; /* if &N.> 0 */

%end; /* do k=1 */

%end; /*if %length(&over_lay.)>0*/

%end; /*if &model.="joint"*/

/* optimal model */

%if &model.="optimal" %then %do;

data temp_sim2; set &indata.; where best3=1; run;

proc sort data=temp_sim2 NODUPKEY; by best3; run; /* in case last 2 models had same LL */

data _null_; set temp_sim2;

call symput('wt_each',wt_final3); call symput('z_tau',tau);

call symput('z_mu',mu);call symput('mtype',modeltype);

run;

/* num of models */

%let N=1000;

%simulate_z1(modeltype=&mtype., nn=&N.);

data _null_;set percentiles;

call symput(_name_,col1);

run;

proc sql noprint;

select max(seqno) into: num_z from newap; /* total number of z_sim data points */

quit;

%let xxx=&num_z.;

/* calc tau based on set_tau of each model run */

%LET z_tau_val=%SYSEVALF(&z_tau.*(&z_p100.-&z_p0.));

/* extract mu */

%if &z_mu.= -5 %then %do;

%let z_mu_val=&z_p_5.;

%end;

%else %if &z_mu.= -10 %then %do;

%let z_mu_val=&z_p_10.;

%end;

%else %if &z_mu.= -15 %then %do;

%let z_mu_val=&z_p_15.;

%end;

%else %do;

%let xx=&z_mu.;

%let z_mu_val=&&z_p&xx.;

%end;

data newap3; set newap2;

array CC{&xxx.} C1-C&xxx.;

%do i=1 %to &xxx.;

%if &mtype.=1 %then %do;

CC{&i.}=(CC{&i.})*(1/(1+exp(-(CC{&i.}-&z_mu_val.)/&z_tau_val.)));

%end;

%if &mtype.=2 %then %do;

CC{&i.}= log(CC{&i.})*(1/(1+exp(-(CC{&i.}-&z_mu_val.)/&z_tau_val.)));

%end;

%end;

run;

%end;

data newap3; set newap3; id=_n_; run;

data newap3; set newap3; where 1<=id<=1000; run;

%mend simulate_z2;

/**************************

* Simulate beta over 1000 realizations

**************************/

%macro simulate_beta(indata=, model=, over_lay=);

%put %upcase(&over_lay.);

%if &model.="joint" %then %do;

/* all models examined */

%if %length(&over_lay.)>0 and %upcase(&over_lay.)=ALL %then %do;

data temp_sim; set &indata.; run;

data temp_sim; set temp_sim; seqno = _n_; run;

proc sql noprint;

select max(seqno) into: num_models from temp_sim; /* total number of models examined */

quit;

%do k=1 %to &num_models.;

data _null_; set temp_sim;

call symput('wt_each',wt); call symput('coef_each',coef);call symput('std_each',stderr);

where seqno=&k.;

run;

%let N=%SYSEVALF(1000*&wt_each., floor);

data sample_&k.(keep=beta);

call streaminit(4321);

do i=1 to &N.;

beta=rand("Normal", &coef_each., &std_each.);

output;

end;

run;

%if &k.=1 %then %do;

data sample_all; set sample_&k.; run;

%end;

%else %do;

data sample_all; set sample_all sample_&k.; run;

%end;

%end;

data simdata; set sample_all; run;

data simdata; set simdata; id=_n_; run;

%end;

/* 3 best models examined */

%if %length(&over_lay.)>0 and %upcase(&over_lay.) ne ALL %then %do;

data temp_sim; set &indata.; where best3>=1; run;

data temp_sim; set temp_sim; seqno = _n_; run;

proc sql noprint;

select max(seqno) into: num_models from temp_sim; /* total number of models examined */

quit;

/* first model */

data _null_; set temp_sim;

call symput('wt_each',wt_final3); call symput('coef_each',coef);call symput('std_each',stderr);

where seqno=1;

run;

%let N=%SYSEVALF(1000*&wt_each., floor);

data sample1(keep=beta);

call streaminit(4321);

do i=1 to &N.;

beta=rand("Normal", &coef_each., &std_each.);

output;

end;

run;

/* second model */

data _null_; set temp_sim;

call symput('wt_each',wt_final3); call symput('coef_each',coef);call symput('std_each',stderr);

where seqno=2;

run;

%let N=%SYSEVALF(1000*&wt_each., floor);

data sample2(keep=beta);

call streaminit(4321);

do i=1 to &N.;

beta=rand("Normal", &coef_each., &std_each.);

output;

end;

run;

/* third model */

data _null_; set temp_sim;

call symput('wt_each',wt_final3); call symput('coef_each',coef);call symput('std_each',stderr);

where seqno=3;

run;

%let N=%SYSEVALF(1000*&wt_each., floor);

data sample3(keep=beta);

call streaminit(4321);

do i=1 to &N.;

beta=rand("Normal", &coef_each., &std_each.);

output;

end;

run;

data sample_all; set sample1; run;

data sample_all; set sample_all sample2; run;

data sample_all; set sample_all sample3; run;

data simdata; set sample_all; run;

data simdata; set simdata; id=_n_; run;

%end;

%end;

/* optimal model */

%if &model.="optimal" %then %do;

data temp_sim2; set &indata.; where best3=1; run;

proc sort data=temp_sim2 NODUPKEY; by best3; run; /* in case last 2 models had same LL */

data _null_; set temp_sim2;

call symput('wt_each',wt_final3); call symput('coef_each',coef); call symput('std_each',stderr);

run;

%let N=1000;

data sample(keep=beta);

call streaminit(4321);

do i=1 to &N.;

beta=rand("Normal", &coef_each., &std_each.);

output;

end;

run;

data sample_all; set sample; run;

data simdata; set sample_all; id=_n_; run;

%end;

%mend simulate_beta;

/**************************

* plot c-r function

**************************/

%macro plot_cr(indata_joint=, indata_optimal=, expo=, unit=, over_lay=);

%if %length(&expo.)=0 %then %do;

%let label2='Air pollution concentration';

%end;

%else %do;

%IF %length(&unit.)>0 %then %do; %let label2="&expo. concentration (&unit.)"; %end;

%IF %length(&unit.)=0 %then %do; %let label2="&expo. concentration"; %end;

%end;

%put &label2.;

%put &unit.;

%if %length(&over_lay.)>0 %then %do;

data &indata_joint.; set &indata_joint.; model="Ensemble"; run;

data &indata_optimal.; set &indata_optimal.; model="Optimal"; run;

data combined; set &indata_joint. &indata_optimal.; run;

ods listing close;

ods html image_dpi=200 file='fitmodel.html' path="&output_path." style=listing;

ods graphics / reset noborder width=600px height=400px imagename="Nonlinear_&sysdate.";

title1 "Nonlinear C-R Curve";

proc sgplot data=combined;

series x=ap y=rr_mean / group=model;

band x=ap lower=rr_lcl upper=rr_ucl / group=model transparency=0.75;

xaxis label=&label2.;

yaxis label='Hazard Ratio (95% CI)';

refline 1;

keylegend / location=inside position=topleft across=1;

run;

ods html close;

ods listing;

%end;

%else %do;

data &indata_optimal.; set &indata_optimal.; model="Optimal"; run;

data combined; set &indata_optimal.; run;

ods listing close;

ods html image_dpi=200 file='fitmodel.html' path="&output_path." style=listing;

ods graphics / reset noborder width=600px height=400px imagename="Nonlinear_&sysdate.";

title1 "Nonlinear C-R Curve";

proc sgplot data=combined;

series x=ap y=rr_mean / group=model;

band x=ap lower=rr_lcl upper=rr_ucl / group=model transparency=0.75;

xaxis label=&label2.;

yaxis label='Hazard Ratio (95% CI)';

refline 1;

keylegend / location=inside position=topleft across=1;

run;

ods html close;

ods listing;

%end;

%mend plot_cr;

*****The Ednd of SAS******

Cox model

################################R#####################################

######################################################################

########################################################################

##

## Nonlinear concentration-response function: model fitting and plotting procedure

##

## Date: Feb 24, 2016

## Version 2.10

##

## Purpose: This program will fit a series of nonlinear concentration-response functions using

## Cox proportional hazards modeling to identify optimal nonlinear relationship

##

## It produces 3 outputs: (1) a graph showing nonlinear relationship based on an optimal model and an ensemble model,

## (2) a summary table listing coefficient, standard error, loglik, and function form of each cox model that was examined, and

## (3) a descriptive table showing distribution of air pollution exposure variable in the origianl and trimmed datasets

##

##

## For more details about this modeling approach, please refer to the accompanying paper Masoud, Szyszkowicz,...,Burnett et al 2016 Air Quality, Atmosphere and Health

##

## Should there be any question with this program, please contact:

## Drs Hong Chen (Hong.Chen@oahpp.ca) and Rick Burnett (Rick.Burnett@hc-sc.gc.ca)

##

##

## Please read the following notes before running the program

##

## 1. All categorical variables should be defined as factor variables before calling bestcox().

## To do this, you may use, for example, inputdata$education <- as.factor(inputdata$education).

##

## 2. When calling bestcox(), you need to define the parameters based on the column names of your input dataset.

##

## 3. Your input dataset should be a dataframe.

##

## 4. By default, the plot will show only nonlinear curve based on optimal model. This can be done either by leaving out

## overlay parameter in the bestcox() or explicitly define overlay=NA.

##

## To overlay ensemble model based on the 3 best models examined, define overlay="best3" in the bestcox().

##

## To overlay ensemble model based on all models examined, define overlay="all" in the bestcox().

##

## 5. By default, the bestcox() will translate air pollution exposure variable such that it has a minimum of unity.

##

## If users do not wish to translate data, you need to specify translate=F or FALSE

##

## 6. By default, output_dir is set as R's default working directory

##

## If users wish to specify a different output directory, you need to ensure to have write permission

##

##

## To illustrate how to use this routine, 5 examples are given below:

##

## Example 1: (fit a time-fixed cox model, without trimming and translating air pollution variable, without strata variable, and overlay ensemble model based on 3 best models)

## out <- bestcox(data = inputdata, translate=F, timename = "time", casename = "status", lowperc = 0, upperc = 100,

## expo_name = "no2", cova_name = c("age","sex"), expo_unit = "ppb", overlay=3, output_dir="F:\\your output directory\\")

## out

##

## Example 2: (fit a time-fixed cox model, with trimmed and translated air pollution variable, without strata variable, and overlay ensemble model based on 3 best models)

## out <- bestcox(data = inputdata, translate=T, timename = "time", casename = "death", lowperc = 1, upperc = 99,

## expo_name = c("pm25"), cova_name = c("age", "sex"), strata_name= NA, expo_unit = c("ug/m3"), overlay="best3", output_dir="F:\\your output directory\\")

## out

##

## Example 3: (fit a time-fixed cox model, with trimmed and translated air pollution variable, with strata variable, and overlay ensemble model based on all models examined)

## out <- bestcox(data = inputdata, timename = "time", casename = "status", lowperc = 1, upperc = 99,

## expo_name = c("no2"), cova_name = c("age"), strata_name= c("sex", "inst"), expo_unit = c("ppb"), overlay="all", output_dir="F:\\your output directory\\")

## out

##

## Example 4: (fit a time-varying cox model, with trimmed and translated air pollution variable, without strata variable, and only show optimal model)

## out <- bestcox(data = inputdata, start = "tstart", end="tstop", casename="status", lowperc = 1, upperc = 99,

## expo_name="no2", cova_name = c("sex", "age", "income", "bmi"), expo_unit = c("ppb"), overlay=NA, output_dir="F:\\your output directory\\")

## out

##

## Example 5: (fit a time-varying cox model, with trimmed and translated air pollution variable, without strata variable, and only show optimal model)

##

## out <- bestcox(data = inputdata, start = c("T1"), end=c("T2"), casename = "Status", lowperc = 1, upperc = 99,

## expo_name = c("pm25"), cova_name = c("bmi"), strata_name= NA, expo_unit = c("ug/m3"), output_dir="F:\\your output directory\\")

## out

##

########################################################################

#########################################################

## function to search for the optimal nonlinear model

#########################################################

library(survival)

library(MASS)

bestcox <- function(data,timename,translate=TRUE,start=NA,end=NA,casename,lowperc,upperc,expo_name,cova_name,strata_name=NA,expo_unit,overlay=NA,output_dir=NA){

data$ap <- data[,expo_name]

data$ap[data$ap<1] <- 1 # convert any conc < 1 to 1

# Characterize the distribution of air pollution exposure variable in the original dataset

size_original <- length(data$ap)

summary_original <- summary(data$ap)

# Trim data, data translation, and tau

data_trim <- subset(data, ap <= quantile(data$ap,upperc/100,na.rm = T) & ap >= quantile(data$ap, lowperc/100, na.rm = T))

rm(data) # remove original dataset to save memory space

if (translate){

ap.min <- min(data_trim$ap, na.rm = T) # translate AP

tran <- 1

} else {

ap.min <- 0 # no translate

tran <- 0

}

data_trim$ap_trans <- data_trim$ap - ap.min + tran # translate z to have the min of 1

data_trim$ap_trans_lm <- data_trim$ap - ap.min # translate z to have the min of 0

data_trim$ap_trans_log <- log(data_trim$ap_trans)

set_tau <- NA

if (length(set_tau)==0 || any(is.na(set_tau)) || set_tau=='') {

# set_tau <- 0.1

tau.2 <- 0.1*(max(data_trim$ap_trans, na.rm = T) - min(data_trim$ap_trans, na.rm = T))

# set_tau <- 0.2

tau.2[2]<- 0.2*(max(data_trim$ap_trans, na.rm = T) - min(data_trim$ap_trans, na.rm = T))

num_tau <- 2

}else{

tau <- set_tau*(max(data_trim$ap_trans, na.rm = T) - min(data_trim$ap_trans, na.rm = T))

num_tau <- 1

}

step_history <- NULL

# Create a function to extract LL from Cox PH model

coxmodel <- function(funcform, loca_perc, method = "breslow"){

# Create f(z)*Wt

if (loca_perc < 0){

loca_para <- min(data_trim$ap_trans, na.rm = T) + (min(data_trim$ap_trans, na.rm = T) - quantile(data_trim$ap_trans, abs(loca_perc)/100, na.rm = T))

loca_para_lm <- min(data_trim$ap_trans_lm, na.rm = T) + (min(data_trim$ap_trans_lm, na.rm = T) - quantile(data_trim$ap_trans_lm, abs(loca_perc)/100, na.rm = T)) # translate z to have the min of 0

}else{

loca_para <- quantile(data_trim$ap_trans, loca_perc/100, na.rm = T)

loca_para_lm <- quantile(data_trim$ap_trans_lm, loca_perc/100, na.rm = T) # translate z to have the min of 0

}

logit_w <- 1/(1+exp(-(data_trim$ap_trans - loca_para)/tau))

logit_w_lm <- 1/(1+exp(-(data_trim$ap_trans_lm - loca_para_lm)/tau)) # translate z to have the min of 0

capture_mu <- NA # capture mu

if (funcform == "linear"){

data_trim$expo <- logit_w_lm * data_trim$ap_trans_lm # translate z to have the min of 0

capture_mu <- loca_para_lm # capture mu

}

if (funcform == "log"){

data_trim$expo <- logit_w * data_trim$ap_trans_log

capture_mu <- loca_para # capture mu

}

if (funcform == "pure.linear"){

data_trim$expo <- data_trim$ap_trans_lm # translate z to have the min of 0

capture_mu <- NA # capture mu

}

if (funcform == "pure.log"){

data_trim$expo <- data_trim$ap_trans_log

capture_mu <- NA # capture mu

}

rm(logit_w) # remove logit_w to save memory space

rm(logit_w_lm) # remove logit_w_lm to save memory space

# Create coxph formula

## without strata variable

if (length(strata_name)==0 || any(is.na(strata_name)) || strata_name=='') {

if (length(start)==0 || any(is.na(start)) || start=='') {

coxformula <- paste("Surv(",timename,",",casename,")~expo+",paste(cova_name,collapse="+"),sep="")

} else {

coxformula <- paste("Surv(",start,",",end,",",casename,")~expo+",paste(cova_name,collapse="+"),sep="")

}

## with strata variable

} else {

if (length(start)==0 || any(is.na(start)) || start=='') {

coxformula <- paste("Surv(",timename,",",casename,")~expo+",paste(cova_name,collapse="+"),"+","strata(",paste(strata_name, collapse=","),")",sep="")

} else {

coxformula <- paste("Surv(",start,",",end,",",casename,")~expo+",paste(cova_name,collapse="+"),"+","strata(",paste(strata_name, collapse=","),")",sep="")

}

}

# Call coxph

coxfit <- coxph(as.formula(coxformula), data = data_trim, method = method)

est <- summary(coxfit)$coefficients[c("expo"),c("coef","se(coef)")]

result <- data.frame(coef = est[1], se.coef=est[2], LL = coxfit$loglik[2], mu=capture_mu) # capture mu

rm(coxfit) # remove coxfit to save memory space

return(result)

} # end of Cox PH model

# Transformation Form Selection (8 or 16 Models, depending if users specify tau)

if (length(set_tau)==0 || any(is.na(set_tau)) || set_tau=='') {

tau <- tau.2[1] # tau=0.1*range

step_a <- data.frame(funcform = c(rep("linear",4),rep("log",4)), loca_perc = rep(c(0,25,50,75),2),

coef = NA, se.coef = NA, LL = NA, mu=NA)

for (i in 1:nrow(step_a)){

step_a[i,c("coef","se.coef","LL", "mu")] <- coxmodel(step_a$funcform[i], step_a$loca_perc[i])

}

step_a$tau <- tau

LL.step_a <- step_a[step_a$LL == max(step_a$LL,na.rm = T),]$LL

tau <- tau.2[2] # tau=0.2*range

step_b <- data.frame(funcform = c(rep("linear",4),rep("log",4)), loca_perc = rep(c(0,25,50,75),2),

coef = NA, se.coef = NA, LL = NA, mu=NA)

for (i in 1:nrow(step_b)){

step_b[i,c("coef","se.coef","LL", "mu")] <- coxmodel(step_b$funcform[i], step_b$loca_perc[i])

}

step_b$tau <- tau

LL.step_b <- step_b[step_b$LL == max(step_b$LL,na.rm = T),]$LL

if (LL.step_a > LL.step_b){

step_0 <- step_a

rejected.models <- step_b # retain rejected tau and related model outputs

rejected.tau <- step_b[1,]$tau # retain rejected tau and related model outputs

}else{

step_0 <- step_b

rejected.models <- step_a # retain rejected tau and related model outputs

rejected.tau <- step_a[1,]$tau # retain rejected tau and related model outputs

}

}else{

step_0 <- data.frame(funcform = c(rep("linear",4),rep("log",4)), loca_perc = rep(c(0,25,50,75),2),

coef = NA, se.coef = NA, LL = NA, mu=NA)

for (i in 1:nrow(step_0)){

step_0[i,c("coef","se.coef","LL", "mu")] <- coxmodel(step_0$funcform[i], step_0$loca_perc[i])

}

step_0$tau <- tau

}

step_history <- step_0

step_history <- subset(step_history, select=-c(tau)) # drop tau

step_0 <- step_0[step_0$LL == max(step_0$LL,na.rm = T),]

funcform <- as.character(step_0$funcform)

tau <- step_0$tau # define best tau

set_tau <- tau/(max(data_trim$ap_trans, na.rm = T) - min(data_trim$ap_trans, na.rm = T)) # best set_tau (0.1 or 0.2)

step_0 <- subset(step_0, select=-c(tau)) # drop tau

set_tau_rejected <- rejected.tau/(max(data_trim$ap_trans, na.rm = T) - min(data_trim$ap_trans, na.rm = T)) # rejected set_tau

# Transformation Location Parameter Selection

#para0 <- step_0$loca_perc

#

#if(para0 == 25){

# step_temp <- cbind(funcform, loca_perc = 0 ,as.vector(coxmodel(funcform, 0)))

# step_0 <- rbind(step_0, step_temp)

# step_history <- rbind(step_history, step_temp)

#}else if(para0 == 50){

# step_temp <- cbind(funcform, loca_perc = 75 ,as.vector(coxmodel(funcform, 75)))

# step_0 <- rbind(step_0, step_temp)

# step_history <- rbind(step_history, step_temp)

#}

#step_0 <- step_0[step_0$LL == max(step_0$LL,na.rm = T),]

para0 <- as.numeric(step_0$loca_perc)

step_temp <- rbind(cbind(funcform, loca_perc = (para0 + 5) ,as.vector(coxmodel(funcform, para0 + 5))),

cbind(funcform, loca_perc = (para0 - 5) ,as.vector(coxmodel(funcform, para0 - 5))))

step_0 <- rbind(step_0, step_temp)

step_history <- rbind(step_history, step_temp)

step_1 <- step_0[step_0$LL == max(step_0$LL,na.rm = T),]

para1 <- as.numeric(step_1$loca_perc)

# STOP if reaching 15% below mu=1 or LL is not smaller

##while(!(para1 %in% c(-5,100,para0))){

while(!(para1 %in% c(-15,100,para0))){

para_temp <- para1 + (para1 - para0)

para0 <- para1

para1 <- para_temp

step_temp <- cbind(funcform, loca_perc = para1 ,as.vector(coxmodel(funcform, para1)))

step_0 <- rbind(step_1, step_temp)

step_history <- rbind(step_history, step_temp)

step_1 <- step_0[step_0$LL == max(step_0$LL,na.rm = T),]

para1 <- as.numeric(step_1$loca_perc)

}

# Reformat step_history and add back rejected model outputs

rownames(step_history) <- NULL

step_history$tau <- set_tau # define best tau (0.1 or 0.2)

step_history_1to8 <- step_history[1:8,]

step_history_9tolast <- step_history[9:length(step_history[,1]),]

rejected.models$tau <- set_tau_rejected # define set_tau_rejected (0.2 or 0.1)

step_history_1to8 <- rbind(step_history_1to8, rejected.models)

step_history <- rbind(step_history_1to8, step_history_9tolast) # re populate "step_history"

# Add ensemble weights for 3 models around the best fit, ie., based on best mu with + and - 5th percentile

step_history_sort3 <- step_history

rownames(step_history_sort3) <- NULL # re start row number

bestLL <- max(step_history_sort3$LL)

step_history_sort3$best3 <- ifelse(step_history_sort3$LL==bestLL, 1, 0)

step_history_sort3$iteration <- rownames(step_history_sort3)

best.models <- step_history_sort3[step_history_sort3$best3==1,]

best.models.sort <- best.models[order(best.models$iteration),]

best.final.LL.iteration <- best.models.sort[length(best.models.sort[,1]),]$iteration

final.mu <- best.models.sort[length(best.models.sort[,1]),]$loca_perc

best.model.form <- best.models.sort[length(best.models.sort[,1]),]$funcform

if (sum(step_history_sort3$best3)==2) {

if (length(step_history_sort3[,1]) > best.final.LL.iteration){

step_history_sort3[length(step_history_sort3[,1]),]$best3 <- 3

} else if (length(step_history_sort3[,1]) == best.final.LL.iteration) {

sec.final.mu <- best.models.sort[(length(best.models.sort[,1])-1),]$loca_perc

if (final.mu > sec.final.mu) {

step_history_sort3[step_history_sort3$loca_perc==(final.mu-10) & step_history_sort3$funcform==best.model.form,]$best3 <- 3

} else {

step_history_sort3[step_history_sort3$loca_perc==(final.mu+10) & step_history_sort3$funcform==best.model.form,]$best3 <- 3

}

}

} else if (sum(step_history_sort3$best3)==1) {

##if (final.mu==-5) {

## step_history_sort3[step_history_sort3$loca_perc==0 & step_history_sort3$funcform==best.model.form,]$best3 <- 2

## step_history_sort3[step_history_sort3$loca_perc==5 & step_history_sort3$funcform==best.model.form,]$best3 <- 3

if (final.mu==-15) {

step_history_sort3[step_history_sort3$loca_perc==-10 & step_history_sort3$funcform==best.model.form,]$best3 <- 2

step_history_sort3[step_history_sort3$loca_perc==-5 & step_history_sort3$funcform==best.model.form,]$best3 <- 3

} else if (final.mu==100) {

step_history_sort3[step_history_sort3$loca_perc==95 & step_history_sort3$funcform==best.model.form,]$best3 <- 2

step_history_sort3[step_history_sort3$loca_perc==90 & step_history_sort3$funcform==best.model.form,]$best3 <- 3

} else {

step_history_sort3[step_history_sort3$loca_perc==(final.mu-5) & step_history_sort3$funcform==best.model.form,]$best3 <- 2

step_history_sort3[step_history_sort3$loca_perc==(final.mu+5) & step_history_sort3$funcform==best.model.form,]$best3 <- 3

}

}

# in rare occasion, models with set_tau_rejected may be assigned best3=2 or 3, thus need to be assigned to 0

step_history_sort3[step_history_sort3$tau==set_tau_rejected,]$best3 <-0

nn <- length(step_history_sort3[step_history_sort3$best3>=1,]$LL) # number of best fit models, normally this should be 3

step_history_sort3$LL.diff <- NA

step_history_sort3[step_history_sort3$best3>=1,]$LL.diff <- exp(step_history_sort3[step_history_sort3$best3>=1,]$LL - min(step_history_sort3[step_history_sort3$best3>=1,]$LL))

step_history_sort3$wt.final3 <- NA

step_history_sort3[step_history_sort3$best3>=1,]$wt.final3 <- step_history_sort3[step_history_sort3$best3>=1,]$LL.diff / sum(step_history_sort3[step_history_sort3$best3>=1,]$LL.diff, na.rm = T)

step_history_sort3<-subset(step_history_sort3, select =-c(LL.diff, best3, iteration))

if (nn >= 1) {

step_history_sort <- step_history_sort3

} else {

step_history_sort <- step_history

step_history_sort$wt <- NA

step_history_sort$wt.final3 <- NA

}

# Add ensemble weights for all the models

step_history_sort$wt <- exp(step_history_sort$LL-min(step_history_sort$LL))/sum(exp(step_history_sort$LL-min(step_history_sort$LL)))

rownames(step_history_sort) <- NULL

# Plot

if (nn >= 1) {

finalmodels.best.nn <- subset(step_history_sort, !is.na(wt.final3)) ## by default, limit to the best nn=3 models

finalmodels.best.nn.final <- finalmodels.best.nn[order(finalmodels.best.nn$LL),]

##if overlay==ALL then ensemble model would include all models examined

if (!is.na(overlay) & toupper(as.character(overlay))=='ALL'){

nn <- length(step_history_sort[,1])

step_history_sort.nn <- step_history_sort

step_history_sort.nn$wt.final3 <- step_history_sort$wt

finalmodels.best.nn.final <- step_history_sort.nn[order(step_history_sort.nn$LL),]

}

##result <- try(plot.bestmodel(ap_data=data_trim$ap, finalmodels=finalmodels.best.nn.final, expo_name=expo_name, unit=expo_unit, nn=nn))

result <- try(plot.bestmodel(ap_data=data_trim$ap, finalmodels=finalmodels.best.nn.final, expo_name=expo_name, unit=expo_unit, nn=nn, overlay=overlay, set_tau=set_tau, set_tau_reject=set_tau_rejected, tran=tran, translate=translate))

if (class(result)=="try-error") {

print("Unable to find joint model! Plot is suppressed and only summary table is produced.")

next

}

}else{

print("Unable to find optimal model! Plot is suppressed and only summary table is produced.")

}

# Pure linear z model: exp(beta*z) (note that loca_perc = 75 is only a place holder)

step_pure_linear <- cbind(funcform="pure.linear", loca_perc = 75 ,as.vector(coxmodel(funcform="pure.linear", 75)))

step_pure_linear$loca_perc <- NA

step_pure_linear$tau <- NA # NA for tau

step_pure_linear$wt.final3 <- NA

step_pure_linear$wt <- NA

rownames(step_pure_linear) <- NULL

# Pure log(z) model: exp(beta*log(z)) (note that loca_perc = 75 is only a place holder)

step_pure_log <- cbind(funcform="pure.log", loca_perc = 75 ,as.vector(coxmodel(funcform="pure.log", 75)))

step_pure_log$loca_perc <- NA

step_pure_log$tau <- NA # NA for tau

step_pure_log$wt.final3 <- NA

step_pure_log$wt <- NA

rownames(step_pure_log) <- NULL

# Output summary table from each step including coef, std, and wt

step_history_sort <- rbind(step_history_sort,step_pure_linear)

step_history_sort <- rbind(step_history_sort,step_pure_log)

##colnames(step_history_sort)[colnames(step_history_sort)=="loca_perc"] <- "location"

colnames(step_history_sort)[2] <- "location"

colnames(step_history_sort)[4] <- "se"

colnames(step_history_sort)[8] <- "finalwt"

# re-order summary table

step_history_sort_final <- step_history_sort[,c("funcform","location","mu","tau","coef","se","LL","wt","finalwt")]

step_history_sort_final$LL <- format(round(step_history_sort_final$LL, 5), nsmall = 6) ## show 6 decimals of LL

#return(step_history_sort_final)

#if (num_tau == 1) {

# step_history_sort_final$tau <- set_tau

#}else{

#step_history_sort_final$tau <- NA

#step_history_sort_final[1:8,]$tau <- 0.1

#step_history_sort_final[9:16,]$tau <- 0.2

#step_history_sort_final[17:length(step_history_sort_final[,1])-2,]$tau <- set_tau

# step_history_sort_final$tau <- set_tau

#}

# write.table(step_history_sort_final, file="search.results.csv", sep = ",", col.names = NA)

write.table(step_history_sort_final, file=paste(output_dir, "search.results.csv", sep=""), sep = ",", col.names = NA)

# add 2 summary tables showing the distribution of air pollution exposure variable in the original and trimmed datasets, respectively

#size_original <- length(data$ap)

size_trimmed <- length(data_trim$ap)

#summary_original <- summary(data$ap)

summary_trimmed <- summary(data_trim$ap)

overall_summary <- list("count_of_obs_in_original_dataset"=size_original,

"distr_of_exp_in_original_dataset"=summary_original,

"count_of_obs_in_trimmed_dataset"=size_trimmed,

"distr_of_exp_in_trimmed_dataset"=summary_trimmed,

"summary_model_fitting"=step_history_sort_final)

# output all 3 summary tables: (1) summary of model fitting and (2) distr of exp var in both original and trimmed datasets

return(overall_summary)

}

#########################################################

## function to plot nonlinear CR relationship

#########################################################

plot.bestmodel <- function(ap_data, finalmodels, expo_name, unit, nn, overlay, set_tau, set_tau_reject, tran, translate){

x <- seq(tran, max(ap_data)-min(ap_data)+tran, 0.1) # based on range of trimmed and translated ap_data

nx <- length(x)

if (finalmodels[length(finalmodels[,1]),]$funcform == "linear") {

x <- x-tran # translate z to have the min of 0

}

if (translate) {

x <- x

} else {

x <- seq(min(ap_data), max(ap_data), 0.1) # if translate=FALSE, use full range of original AP data

nx <- length(x)

}

# prepare x1 for use in simulation, varying depending on perc, set_tau, and funcform

sim.x1 <- function(x_sim, perc, set_tau_sim, funcform_sim){

if (perc < 0){

mu <- min(x_sim, na.rm = T) + (min(x_sim, na.rm = T)-quantile(x_sim, abs(perc)/100, na.rm = T))

}else{

mu <- quantile(x_sim, perc/100, na.rm = T)

}

tau_sim <- set_tau_sim*(max(x_sim)-min(x_sim))

logit <- exp((x_sim-mu)/tau_sim)/(1+exp((x_sim-mu)/tau_sim))

if (funcform_sim== "linear"){

x1<-x_sim*logit

}

if (funcform_sim == "log"){

x1<-log(x_sim)*logit

}

# x1 <- x1-min(x1) # set min(x1) as reference, thus beta*min(x1)=0

return(x1)

}

# Consider only optimal model - simulate 1000 realizations based on se.coef alone

nsim<-1000

ran<-matrix(0, nsim, 1)

rr<-matrix(0, nsim, nx)

medRR<-matrix(0, nx, 1)

upcl<-matrix(0, nx, 1)

lowcl<-matrix(0, nx, 1)

loca_perc_sim <- finalmodels[finalmodels$LL==max(finalmodels$LL, na.rm=T),]$loca_perc

funcform_sim <- finalmodels[length(finalmodels[,1]),]$funcform

x1 <- sim.x1(x_sim=x, perc=loca_perc_sim, set_tau_sim=set_tau, funcform_sim=funcform_sim)

for (i in 1:nsim) {

ran[i,]<-rnorm(1, finalmodels[length(finalmodels[,1]),]$coef, finalmodels[length(finalmodels[,1]),]$se.coef)

for (j in 1:length(x)) {

rr[i,j]<-exp(ran[i,1]*x1[j])

}

}

for (j in 1:length(x)) {

medRR[j] <- mean(rr[,j])

lowcl[j] <- quantile(rr[,j], 0.025)

upcl[j] <- quantile(rr[,j], 0.975)

}

# Incorporate 3 best models or ALL models - simulate 1000 realizations based on se.coef AND weights derived from LL

if (nn >= 2) {

nsim<-1000

ran.3<-matrix(0, nsim, 1)

rr.3<-matrix(0, nsim, nx)

medRR.3<-matrix(0, nx, 1)

upcl.3<-matrix(0, nx, 1)

lowcl.3<-matrix(0, nx, 1)

pp <- 1 # position variable in the 1000 sim

nn <- nn # consider top 3 models

nsim.sum <- 0 # count of nsim to ensure the last run lead to rownum of exactly 1000

# k from 0 to nn-1: varying depending on models included and pooled

for (k in 0:(nn-1)) {

nsim.wt <- nsim * round(finalmodels[length(finalmodels[,1])-k,]$wt.final3, digits = 3)

loca_perc_sim <- finalmodels[length(finalmodels[,1])-k,]$loca_perc

funcform_sim <- finalmodels[length(finalmodels[,1])-k,]$funcform

if (finalmodels[length(finalmodels[,1])-k,]$tau==set_tau_reject){

x1 <- sim.x1(x_sim=x, perc=loca_perc_sim, set_tau_sim=set_tau_reject, funcform_sim=funcform_sim)

}else{

x1 <- sim.x1(x_sim=x, perc=loca_perc_sim, set_tau_sim=set_tau, funcform_sim=funcform_sim)

}

if (k==nn-1) {nsim.wt <- nsim - nsim.sum}

for (i in pp:(pp+nsim.wt-1)) {

# for models with weights~0, i may exceed 1000 thus throw an error on out of bound

if (i<=1000){

ran.3[i,]<-rnorm(1, finalmodels[length(finalmodels[,1])-k,]$coef, finalmodels[length(finalmodels[,1])-k,]$se.coef)

for (j in 1:length(x)){

rr.3[i,j]<-exp(ran.3[i,1]*x1[j])

}

}

}

pp <- pp+nsim.wt

nsim.sum <- nsim.sum+nsim.wt

}

for (j in 1:length(x)) {

medRR.3[j] <- mean(rr.3[,j])

lowcl.3[j] <- quantile(rr.3[,j], 0.025)

upcl.3[j] <- quantile(rr.3[,j], 0.975)

}

}

# transform x back to original scale of AP data for plotting

if (translate) {

if (finalmodels[length(finalmodels[,1]),]$funcform == "linear") {

x <- x+min(ap_data) # translate z to have the min of 0

} else {

x <- x+min(ap_data)-tran # translate z to have the min of 1

}

}

##whether or not overlay ensemble model

if (length(overlay)==0 || any(is.na(overlay)) || overlay==''){

# plot optimal model only

par(las = 1 , cex = 1)

# use predicted values from optimal model for the plot

medRR.3 <- medRR

lowcl.3 <- lowcl

upcl.3 <- upcl

plot(x, upcl.3, lwd=4, type="l", col="#DEEBF7", frame.plot=T, ylim=c(min(lowcl.3, na.rm=TRUE)-0.25, max(upcl.3, na.rm=TRUE)+0.25), ylab="Hazard Ratio", xlab=paste(toupper(expo_name), " (", unit, ")"))

polygon(x=c(x, rev(x)), y=c(lowcl.3, rev(upcl.3)), col="#DEEBF7", border=NA, lty=2)

lines(x, medRR.3, lwd=3, col="#08519C")

lines(x, lowcl.3, lwd=2, col="#DEEBF7")

nn <- 1000

} else {

# plot joint model

par(las = 1 , cex = 1)

if (nn < 2) {

# if only optimal model exists, use predicted values from the optimal model for the plot

medRR.3 <- medRR

lowcl.3 <- lowcl

upcl.3 <- upcl

}

# in rare occasion, max(upcl.3)==Inf and/or min(lowcl.3)==Inf

upcl.3.excluded.inf <- upcl.3[upcl.3<100]

max.ylim <- max(upcl.3.excluded.inf, na.rm=TRUE)+0.25

if (is.na(max.ylim)){max.ylim <- 5}

lowcl.3.excluded.inf <- lowcl.3[lowcl.3<100]

min.ylim <- min(lowcl.3.excluded.inf, na.rm=TRUE)-0.25

if (is.na(min.ylim)){min.ylim <- 0}

plot(x, upcl.3, lwd=4, type="l", col="#DEEBF7", frame.plot=T, ylim=c(min.ylim, max.ylim), ylab="Hazard Ratio", xlab=paste(toupper(expo_name), " (", unit, ")"))

polygon(x=c(x, rev(x)), y=c(lowcl.3, rev(upcl.3)), col="#DEEBF7", border=NA, lty=2)

lines(x, medRR.3, lwd=3, col="#08519C")

lines(x, lowcl.3, lwd=2, col="#DEEBF7")

# Overlay optimal model

lines(x, medRR, lwd=2, col="red")

lines(x, lowcl, lwd=1, lty=2, col="red")

lines(x, upcl, lwd=1, lty=2, col="red")

}

# Add rugs, legend, and reference line

## add rugs=ticks at datapoints

####axis(side = 1 , line = -1.2 , at = jitter(x) , labels = F , tick = T , tcl = 0.8 , lwd.ticks = 0.1 , lwd = 0)

## rugs and labels at 1Q, median and 3Q

####axis(side = 1 , line = -1.0 , at = fivenum(x)[2:4], lwd = 0 , tick = T, tcl = 1.2 , lwd.ticks = 1 , col.ticks = "black" , labels = c("Quartile 1","Median","Quartile 3"), cex.axis = 0.7, col.axis = "black" , padj = -2.8)

####axis(side = 1 , line = 0.0 , at = fivenum(x)[2:4], lwd = 0 , tick = T, tcl = 0.2 , lwd.ticks = 1 , col.ticks = "black", labels = FALSE)

## add legend and RR=1 line

if (nn > 100) {

legend("topleft", inset=c(0,0), c("optimal model"), col=c("blue"), lty=1, lwd=3)

} else if (nn >= 2) {

legend("topleft", inset=c(0,0), c("ensemble model", "optimal model"), col=c("blue", "red"), lty=1, lwd=3)

} else {

legend("topleft", inset=c(0,0), c("optimal model", "optimal model"), col=c("blue", "red"), lty=1, lwd=3)

}

abline(1,0, col = "gray", lty=3, lwd=1)

box(bty = "n")

}

#############################################################################################################################################The end of R

Conditional logistic

$$$$$$$$$$$$$$$$$$$$$$$$$$$$$$$$$$$$$$$$$$$$$$$$$$$$$$$$$$$$$$$$$$$$$$$$$$$$$$$$$$$$$$$$$$$$$$$$$$$$$$$$$$$$$$$$$$$$$$$$$$$$$$$$$$$$$$$$$$$$$$$$$$$$$$$$$$$$$$$$$$$$$$$$$$$$$$$$$$$$$$$$$$$$$$$$$$$$$$$$$$$$$$SAS$$$$$$$$$$$$$$$$$$$$$$$$$$$$$$$$$$$$$$

/*************************************************************************************************

*

* Nonlinear concentration-response function: model fitting and plotting procedure

*

* Date: Feb 24, 2016

* Version 2.10

*

* Purpose: This macro will fit a series of nonlinear concentration-response functions using

* conditional logistic modeling to identify optimal nonlinear relationship

*

* It will produce three outputs to the file directory specified by users:

*

* (1) a graph (.png) showing nonlinear relationship based on an optimal model, and if pre-specified, it will overlay an ensemble model,

* based on either all models examined or the best 3 models, according to their -2LogLik

* (2) a summary table (.pdf) listing coefficient, standard error, loglik, and function form of each logistic regression model that was fitted

* (3) a summary table (.pdf) showing descriptive stats of air pollution exposure variable from the original and the trimmed datasets

*

* For more details about this modeling approach, please refer to the accompanying paper Masoud, Szyszkowicz,...,Burnett et al 2016 Air Quality, Atmosphere and Health

*

* Should there be any question with this macro, please contact:

* Drs Hong Chen (Hong.Chen@oahpp.ca) and Rick Burnett (Rick.Burnett@hc-sc.gc.ca)

*

*

* Please read the following notes before running this macro

*

* 1. All categorical variables should to be prepared as a series of dichotomous variables prior to calling macro

*

* 2. Exposure variable need to be define by using "fitvar" parameter

*

* 3. The following 2 parameters are optional: "label_exposure" and "label_unit"

*

* 4. To run conditional logistic model, please specify "case" and "time", and omit "start" and "stop" parameters.

*

* Time variable can be define as: time=2-outcome, prior to invoking this model fitting function.

*

* 5. If users do not wish to translate data, please specify translate=N or NO

*

* 6. By default, the plot displays a concentration-response curve based on the optimal model only.

* To overlay a c-r curve using ensemble model, users need to specify "overlay" parameters.

* By defining overlay=all or overlay=ALL, users can overlay ensemble model based on all models examined.

* By defining overlay=any text string (except for "all" or "ALL"), users can overlay ensemble model based on the 3 best models.

*

*

*

* To illustrate how to use this macro, 4 examples are given below:

*

* Example 1: (translate data, with 1 case matching with n controls on age, only show optimal model)

*

* %fitap(datain=cohort_ABC,perc_trim=1,translate=yes,dataout=allmodels,case=case,time=time,fitvar=no2,covvars=sex, strata=age,

* label_exposure=NO2, label_unit=ppb, output_path=D:\Working directory\Cohort);

*

* Example 2: (translate data, with 1 case matching with n controls on age and sex, only show optimal model)

*

* %fitap(datain=cohort_ABC,perc_trim=5,translate=YES,dataout=allmodels,case=case,time=time,fitvar=pm25,covvars=ses,strata=age sex,

* label_exposure=pm25, label_unit=ug/m3, output_path=D:\Working directory\Cohort);

*

* Example 3: (no translate data, with 1 case matching with n controls on match_id and age, overlay ensemble model based on all models examined)

*

* Note that to specify an ensemble model based on all models examined, please define overlay=all or overlay=ALL

*

* %fitap(datain=cohort_ABC,perc_trim=1,translate=no,dataout=allmodels,case=case,time=time,fitvar=pm25,covvars=sex ses income bmi,strata=match_id age,

* label_exposure=pm25, label_unit=ug/m3, output_path=D:\Working directory\Cohort, overlay=all);

*

* Example 4: (no translate data, with 1 case matching with n controls on age and sex, overlay ensemble model based on best 3 models examined)

*

* Note that to specify an ensemble model based on best 3 models examined, please define overlay=any text string, except for "all" or "ALL"

*

* %fitap(datain=cohort_ABC,perc_trim=1,translate=No,dataout=allmodels,case=Status,time=time,fitvar=pm25,covvars=ses obese income smk,strata=age sex,

* label_exposure=pm25, label_unit=ug/m3, output_path=D:\Working directory\Cohort, overlay=3);

*

*************************************************************************************************/

/**************************

* main function to select optimal model and ensemble model

***************************/

option LINESIZE=MAX;

%macro fitap(datain=,perc_trim=0,translate=yes,dataout=,case=,time=,start=,stop=,fitvar=,covvars=,strata=,label_exposure=,label_unit=,overlay=,output_path=);

/*Clean up any existing global macro variables*/

%symdel low_pct low_wc low_pct_1 low_wc_1 linear_model time start stop LL_min_all sum_LL_all LL_min_3 sum_LL_3;

/*Consider non-linear models first*/

%let linear_model = 0;

/*convert any conc < 1 to 1*/

data &datain.; set &datain.;

if &fitvar.<1 then &fitvar.=1;

run;

/*Trim data*/

%if 0<=&perc_trim.<= 10 %then %do;

%let trim_l=%SYSEVALF(&perc_trim.);

%let trim_r=%SYSEVALF(100-&perc_trim.);

%end;

%else %do; %put "WARNING: perc_trim must be an integer between 0 and 10"; %abort;%end;

proc univariate data=&datain. noprint;

var &fitvar.;

output out=percentiles pctlpts=&trim_l. &trim_r. pctlpre=ppp;

run;

data _null_;set percentiles;call symput('pctll',ppp%left(&trim_l.));call symput('pctlr',ppp%left(&trim_r.));run;

data aftertrim; set &datain.; where &pctll.<=&fitvar.<=&pctlr.; run;

/*Descriptive statistics of exp variable in the original and trimmed datasets*/

proc means data=&datain. n nmiss min q1 mean median q3 max;

var &fitvar.;

output out=stats_original n=total_obs nmiss=miss_obs min=min_ap q1=q1_ap mean=mean_ap median=median_ap q3=q3_ap max=max_ap;

run;

proc means data=aftertrim n nmiss min q1 mean median q3 max;

var &fitvar.;

output out=stats_aftertrim n=total_obs nmiss=miss_obs min=min_ap q1=q1_ap mean=mean_ap median=median_ap q3=q3_ap max=max_ap;

run;

data stats_original (drop=_type_ _freq_); set stats_original;

data_description="original dataset";

exp_var="&fitvar.";

run;

data stats_aftertrim (drop=_type_ _freq_); set stats_aftertrim;

data_description="trimmed dataset";

exp_var="&fitvar.";

run;

data overall_stat; set stats_original stats_aftertrim; run;

/*Translate data*/

%put "check translate";

%put &translate.;

%put &pctll.;

%put &pctlr.;

%if %upcase(&translate.)= YES or %upcase(&translate.)= Y %then %do;

%let Tran=1; /* translate z to have the min of 1*/

%end;

%else %do;

%let Tran=0; /* no translate */

%let pctll=0; /* no translate */

%end;

%put &Tran.;

/*retain an original copy to be reused at each time when modelpct() is called*/

data aftertrim_backup; set aftertrim; run;

/*set tau=0.1*/

%let set_tau=0.1;

%let model_tau=1;

/* %LET tau=%SYSEVALF(&set_tau.*(&p100.-&p0.)); */

%LET count=0;

/*run 8 models to decide the model type and pct with smallest coefficient*/

%do mdltp=1 %TO 2;

%do pctt=0 %TO 75 %BY 25;

%modelpct(modeltype=&mdltp.,pct=&pctt.);

%end;%end;

data fourmodel_1;set mtll_:; run;

proc sort data=fourmodel_1;by WithCovariates;run;

data fourmodel_1;set fourmodel_1; if _N_=1;run;

data _null_;set fourmodel_1;

call symput('modeltp_a',modeltype); call symput('low_pct_a',pct); call symput('low_wc_a',WithCovariates);

run;

/*set tau=0.2*/

%let set_tau=0.2;

%let model_tau=2;

/* %LET tau=%SYSEVALF(&set_tau.*(&p100.-&p0.)); */

%LET count=0;

/*run 8 models to decide the model type and pct with smallest coefficient*/

%do mdltp=1 %TO 2;

%do pctt=0 %TO 75 %BY 25;

%modelpct(modeltype=&mdltp.,pct=&pctt.);

%end;%end;

data fourmodel_2;set mtll_:; run;

proc sort data=fourmodel_2;by WithCovariates;run;

data fourmodel_2;set fourmodel_2; if _N_=1;run;

data _null_;set fourmodel_2;

call symput('modeltp_b',modeltype); call symput('low_pct_b',pct); call symput('low_wc_b',WithCovariates);

run;

/*compare and find the optimal tau*/

%if &low_wc_a.<=low_wc_b. %then %do;

%let modeltp=&modeltp_a.;

%let low_pct=&low_pct_a.;

%let low_wc=&low_wc_a.;

%let set_tau=0.1;

/* %LET tau=%SYSEVALF(&set_tau.*(&p100.-&p0.)); */

%let model_tau=9;

%put &set_tau.;

%put &model_tau.;

/*retain rejected tau and related models*/

data dataout_reject; set dataout_2; call symput('set_tau_reject',0.2); run;

%end;

%else %do;

%let modeltp=&modeltp_b.;

%let low_pct=&low_pct_b.;

%let low_wc=&low_wc_b.;

%let set_tau=0.2;

/* %LET tau=%SYSEVALF(&set_tau.*(&p100.-&p0.)); */

%let model_tau=9;

%put &set_tau.;

%put &model_tau.;

/*retain rejected tau and related models*/

data dataout_reject; set dataout_1; call symput('set_tau_reject',0.1); run;

%end;

/*compare -5/+5 percentile around low_pct from above, which is either 0 or 25 or 50 or 75 */

%let low_wc_1=&low_wc.;

%let low_pct_1=&low_pct.;

%do pct_=(&low_pct.+5) %to (&low_pct.-5) %by -10;

%modelpct(modeltype=&modeltp., pct=&pct_.);

data _null_; set fits; call symput('new_wc',WithCovariates); call symput('new_pct',pct); run;

%if &new_wc.<&low_wc. %then %do; %let low_wc_1=&new_wc.; %let low_pct_1=&new_pct.; %end;

%end;

/* STOP if reaching mu=-15th, 100th, or LL is no longer smaller */

%do %while ( &low_pct_1. >= -10 and &low_pct_1. <= 95 and &low_pct_1. NE &low_pct.);

%let low_pct_temp = %SYSEVALF(&low_pct_1. + (&low_pct_1. - &low_pct.));

%let low_pct=&low_pct_1.;

%modelpct(modeltype=&modeltp., pct=&low_pct_temp.);

data _null_; set fits; call symput('new_wc',WithCovariates); call symput('new_pct',pct); run;

%if &new_wc.<&low_wc_1. %then %do; %let low_wc_1=&new_wc.; %let low_pct_1=&new_pct.; %end;

%end;

proc datasets;delete mtll: percentiles fourmodel fits ;run;quit;

/*drop last run if mu was -20 or 105*/

data &dataout.; set &dataout.; where (pct NE -20); run;

data &dataout.; set &dataout.; where (pct NE 105); run;

/*add tau and append the rejected model outputs*/

proc sort data=&dataout.; by iteration; run;

data &dataout.; set &dataout.; tau=&set_tau.; run;

data dataout1_8; set &dataout.; where iteration<=8; run;

data dataout9_n; set &dataout.; where iteration>8; run;

data dataout9_n; set dataout9_n; iteration=iteration+8; run;

proc sort data=dataout_reject;by iteration;run;

data dataout_reject; set dataout_reject; tau=&set_tau_reject.; iteration=iteration+8; run;

data &dataout.; set dataout1_8 dataout_reject; run;

data &dataout.; set &dataout. dataout9_n; run;

/* print out chosen percentage and corresponding coeffieent */

data &dataout.;set &dataout.;

rename pct=mu;

rename pctl=z_at_mu;

drop WithoutCovariates;

run;

proc sort data=&dataout.;by iteration;run;

/* Calculate ensemble weights using 3 models around the best fit, ie., based on best mu with +/- 5th%

The three model include last 2 models from the search + a 3rd model corresponding mu+5 of last model */

%symdel best_LL final_mu;

data &dataout. (drop=WithCovariates); set &dataout.; format LL d18.5; LL=WithCovariates; run;

data &dataout.; set &dataout.; rename LL=WithCovariates; run;

/* find optimal model corresonding to minimum LL */

proc sql noprint;

select min(WithCovariates) into: best_LL from &dataout.;

quit;

data &dataout.; set &dataout.; id=1; run;

data qaqc1; set &dataout.; run;

proc sort data=qaqc1; by WithCovariates; run;

data qaqc2; set qaqc1; id=_n_; run;

data qaqc2; set qaqc2; rename WithCovariates=best_LL2; where id=1; run;

data &dataout.; merge &dataout. (in=fro) qaqc2 (keep=id best_LL2); by id; if fro; run;

data &dataout.; set &dataout.;

if (WithCovariates = best_LL2) then do;

best3=1; call symput('final_mu',mu);

end;

run;

data &dataout.; set &dataout.; drop id best_LL2; run;

data &dataout.; set &dataout.; final_mu=&final_mu.; final_form=&modeltp.; run;

/* find 2 other alternative models */

proc sql noprint;

select sum(best3) into: count_best_LL from &dataout.; /* num of models with same smallest LL */

quit;

proc sql noprint;

select max(iteration) into: last_iteration from &dataout.; /* iteration id for last run */

quit;

%if &count_best_LL.=2 %then %do; /* a special case where last 2 runs had same smallest LL */

data &dataout.; set &dataout.; if mu=final_mu and tau=&set_tau. then call symput('best_final_LL_iteration',iteration); run;

%if &last_iteration. > &best_final_LL_iteration. %then %do; /* followed by additional run with a larger LL*/

data &dataout.; set &dataout.;

if iteration eq &last_iteration. then best3=3;

run;

%end;

%if &last_iteration. eq &best_final_LL_iteration. %then %do; /* followed by no more additional run*/

data &dataout.; set &dataout.;

if &new_pct. > &low_pct_1. and mu=(final_mu-10) and final_form=modeltype and tau=&set_tau. then best3=3; /* ascending*/

else if &new_pct. < &low_pct_1. and mu=(final_mu+10) and final_form=modeltype and tau=&set_tau. then best3=3; /* descending*/

run;

%end;

%end;

%if &count_best_LL.=1 %then

%do;

data &dataout.; set &dataout.;

if final_mu=-15 then do; /* a special case where last run reached mu=-15 */

if mu=-10 and tau=&set_tau. then best3=2;

if mu=-5 and tau=&set_tau. then best3=3;

end;

else if final_mu=100 then do; /* a special case where last run reached mu=100 */

if mu=95 and tau=&set_tau. then best3=2;

if mu=90 and tau=&set_tau. then best3=3;

end;

else do; /* all other cases */

if mu=(final_mu-5) and final_form=modeltype and tau=&set_tau. then best3=2;

if mu=(final_mu+5) and final_form=modeltype and tau=&set_tau. then best3=3;

end;

run;

%end;

/* derive LL from -2LL */

data &dataout.; set &dataout.;

LL = WithCovariates/(-2);

run;

/* calculate ensemble weights for all models */

proc sql noprint;

select min(LL) into: LL_min_all from &dataout.;

quit;

data &dataout.; set &dataout.;

LL_diff = exp(LL-&LL_min_all.);

run;

proc sql noprint;

select sum(LL_diff) into: sum_LL_all

from &dataout.;

quit;

data &dataout.; set &dataout.; wt=LL_diff/&sum_LL_all.;run;

data &dataout.; set &dataout.; drop LL_diff; run;

/* calculate ensemble weights for 3 final models */

data final3models; set &dataout.; where best3>=1; run;

proc sql noprint;

select min(LL) into: LL_min_3 from final3models;

quit;

data final3models; set final3models; LL_diff = exp(LL-&LL_min_3.); run;

proc sql noprint;

select sum(LL_diff) into: sum_LL_3 from final3models;

quit;

data &dataout.; set &dataout.;

if best3>=1 then LL_diff = exp(LL-&LL_min_3.);

else LL_diff=.;

run;

data &dataout.; set &dataout.; wt_final3=LL_diff/&sum_LL_3.;run;

data &dataout.; set &dataout.; if best3=. then wt_final3=.; drop LL_diff; run;

/* Bootstrap to sample beta based on joint model */

%if %length(&overlay.)>0 %then %do;

%simulate_z2(indata=&dataout., model="joint", over_lay=&overlay.);

proc sort data=newap3; by id; run;

%simulate_beta(indata=&dataout., model="joint", over_lay=&overlay.);

proc sort data=simdata; by id; run;

data newap3; merge newap3(in=fro) simdata; by id; if fro; run;

/* count max num of sim ap data points */

proc contents data=newap3 out=output_z; run;

proc sort data=output_z; by varnum; run;

data _null_;

set output_z;

call symputx("maximum",varnum-2);

run;

/* beta*transformed(z) for each simulated z data points */

data newap3 (drop=beta id); set newap3;

array CC{&maximum.} C1-C&maximum.;

do i=1 to &maximum.;

CC{i}=exp(beta*CC{i});

end;

run;

/* derive median and 2.5th% and 97th% */

%do i=1 %to &maximum.;

data newap_&i.; set newap3;

keep C&i.;

run;

proc univariate data=newap_&i. noprint;

var C&i.;

output out=beta_distr_&i. pctlpts= 2.5 50 97.5 pctlpre=P;

run;

data beta_distr_&i.;set beta_distr_&i.; id=&i.; run;

%if &i.=1 %then %do;

data beta_distr; set beta_distr_&i.; run;

%end;

%else %do;

data beta_distr; set beta_distr beta_distr_&i.; run; /* contain 4 variables: id, p2.5, p50, p97.5 */

%end;

%end;

data newap4(keep = z_sim);

do i = &pctll. to &pctlr. by 0.1;

z_sim = i;

output;

end;

run;

data newap4; set newap4; id=_n_; run;

proc sort data=newap4; by id; run;

proc sort data=beta_distr; by id; run;

data beta_distr; merge beta_distr(in=fro) newap4; if fro; by id; run;

%if %upcase(&translate.)= YES or %upcase(&translate.)= Y %then %do;

%if &modeltp.=1 %then %do;

data newap_joint; set beta_distr;

rename p50=rr_mean;

rename p97_5=rr_ucl;

rename p2_5=rr_lcl;

ap = z_sim;

run;

%end;

%else %if &modeltp.=2 %then %do;

data newap_joint; set beta_distr;

rename p50=rr_mean;

rename p97_5=rr_ucl;

rename p2_5=rr_lcl;

ap = z_sim;

run;

%end;

%end;

%else %do;

data newap_joint; set beta_distr;

rename p50=rr_mean;

rename p97_5=rr_ucl;

rename p2_5=rr_lcl;

ap = z_sim;

run;

%end;

%end;

/* Bootstrap to sample beta based on optimal model */

%simulate_z2(indata=&dataout., model="optimal");

proc sort data=newap3; by id; run;

%simulate_beta(indata=&dataout., model="optimal");

proc sort data=simdata; by id; run;

data newap3; merge newap3(in=fro) simdata; by id; if fro; run;

/* count max num of sim ap data points */

proc contents data=newap3 out=output_z; run;

proc sort data=output_z; by varnum; run;

data _null_;

set output_z;

call symputx("maximum",varnum-2);

run;

/* beta*transformed(z) for each simulated z data points */

data newap3 (drop=beta id); set newap3;

array CC{&maximum.} C1-C&maximum.;

do i=1 to &maximum.;

CC{i}=exp(beta*CC{i});

end;

run;

/* derive median and 2.5th% and 97th% */

%do i=1 %to &maximum.;

data newap_&i.; set newap3;

keep C&i.;

run;

proc univariate data=newap_&i. noprint;

var C&i.;

output out=beta_distr_&i. pctlpts= 2.5 50 97.5 pctlpre=P;

run;

data beta_distr_&i.;set beta_distr_&i.; id=&i.; run;

%if &i=1 %then %do;

data beta_distr; set beta_distr_&i.; run;

%end;

%else %do;

data beta_distr; set beta_distr beta_distr_&i.; run; /* contain 4 variables: id, p2.5, p50, p97.5 */

%end;

%end;

data newap4(keep = z_sim);

do i = &pctll. to &pctlr. by 0.1;

z_sim = i;

output;

end;

run;

data newap4; set newap4; id=_n_; run;

proc sort data=newap4; by id; run;

proc sort data=beta_distr; by id; run;

data beta_distr; merge beta_distr(in=fro) newap4; if fro; by id; run;

%if %upcase(&translate.)= YES or %upcase(&translate.)= Y %then %do;

%if &modeltp.=1 %then %do;

data newap_optimal; set beta_distr;

rename p50=rr_mean;

rename p97_5=rr_ucl;

rename p2_5=rr_lcl;

ap = z_sim;

run;

%end;

%else %if &modeltp.=2 %then %do;

data newap_optimal; set beta_distr;

rename p50=rr_mean;

rename p97_5=rr_ucl;

rename p2_5=rr_lcl;

ap = z_sim;

run;

%end;

%end;

%else %do;

data newap_optimal; set beta_distr;

rename p50=rr_mean;

rename p97_5=rr_ucl;

rename p2_5=rr_lcl;

ap = z_sim;

run;

%end;

/* plot joint and optimal models */

%plot_cr(indata_joint=newap_joint, indata_optimal=newap_optimal, expo=&label_exposure., unit=&label_unit., over_lay=&overlay.);

/* add a pure linear model with z */

%let linear_model = 1;

%put &linear_model.;

%put &modeltp.;

%put &low_pct_1.;

%modelpct(modeltype=&modeltp., pct=&low_pct_1.);

/* add a pure log(z) model */

%let linear_model = 2;

proc datasets;delete fits ;run;quit;

%modelpct(modeltype=&modeltp., pct=&low_pct_1.);

/* print description of original/trimmed datasets and model fitting, and clean up working directory */

ods pdf file="&output_path.\model_fitting_summary.pdf";

title;

proc print data=overall_stat;

title "Summary statistics of air pollution exposure variable in the original and trimmed datasets";

run;

title;

title;

data &dataout.;set &dataout. (drop=LL);

length model_function $15.;

if modeltype=1 then model_function='z*logit';

else if modeltype=2 then model_function='log(z)*logit';

else if modeltype=3 then model_function='pure linear';

else if modeltype=4 then model_function='pure log';

rename WithCovariates=LL;

drop WithoutCovariates pct pctl best3 final_form final_mu modeltype;

run;

/* proc sort data=&dataout.;by iteration;run; */

data &dataout.; set &dataout.; drop iteration Criterion; run; /* simplif summary table so as to fit in one page */

data &dataout.; set &dataout.; rename mu=location; run;

data &dataout.; set &dataout.; rename z_at_mu=mu; rename wt_final3=final_wt; run;

/* data &dataout.; set &dataout.; tau=&set_tau.; run; */

data prt; set &dataout.; run;

proc print data=prt;

title "Summary description of model fitting";

run;

title;

ods pdf close;

/* export output table to csv format */

proc export data=&dataout.

outfile="&output_path.\model_fitting_summary.csv"

dbms=csv

replace;

run;

/* clean up non-essential datasets */

proc datasets library=work;

delete ssample: sample_all beta_distr final3models

newap newap_joint newap_optimal param prt overall_stat simdata temp_sim temp_sim2 qaqc: percentiles output_z

Z_sim_distr new_datain mtll: fits combined Beta_distr: dataout: newap: ;

run;quit;

%Mend fitap;

/**************************

* fit cox PH model with z

**************************/

%macro modelpct(modeltype=,pct=);

/*translate ap data to have the min of 1 or 0, depending on log or linear model*/

/*note that this only applies to translate=Y*/

/*note that if translate=N, then &pctll.=0 and &Tran.=0*/

/*for log model: translate dataset to have the min of 1*/

%if &modeltype.=2 %then %do;

data aftertrim; set aftertrim_backup; z=&fitvar.-&pctll.+&Tran.; run;

%end;

/*for linear model: translate dataset to have the min of 0*/

%else %if &modeltype.=1 %then %do;

data aftertrim; set aftertrim_backup; z=&fitvar.-&pctll.; run;

%end;

proc sql noprint;

select max(z) into: p100 from aftertrim;

select min(z) into: p0 from aftertrim;

quit;

proc univariate data=aftertrim noprint;

var z;

output out=percentiles pctlpts= 5 to 95 by 5 pctlpre=P;

run;

data percentiles; set percentiles; p_5=&p0.-(p5-&p0.); run; /* add a variable p_5 denoting 5 pctl below p0*/

data percentiles; set percentiles; p_10=&p0.-(p10-&p0.); run; /* add a variable p_10 denoting 10 pctl below p0*/

data percentiles; set percentiles; p_15=&p0.-(p15-&p0.); run; /* add a variable p_15 denoting 15 pctl below p0*/

proc transpose data=percentiles out=percentiles;run;

data _null_;set percentiles;

call symput(_name_,col1);

run;

/* calc tau based on set_tau */

%LET tau=%SYSEVALF(&set_tau.*(&p100.-&p0.));

/*calc log transformed ap data*/

%if &pct.= -5 %then %do;

%let mu=&p_5.; /* if 5 percentile less than p0, then change to _5 */

data aftertrim; set aftertrim;

%if &modeltype.=1 %then %do;

APvar=(z)*(1/(1+exp(-(z-&mu.)/&tau.)));

%end;

%if &modeltype.=2 %then %do;

APvar= log(z)*(1/(1+exp(-(z-&mu.)/&tau.)));

%end;run;

%end;

%else %if &pct. = -10 %then %do;

%let mu=&p_10.; /* if 10 percentile less than p0, then change to _10 */

data aftertrim; set aftertrim;

%if &modeltype.=1 %then %do;

APvar=(z)*(1/(1+exp(-(z-&mu.)/&tau.)));

%end;

%if &modeltype.=2 %then %do;

APvar= log(z)*(1/(1+exp(-(z-&mu.)/&tau.)));

%end;run;

%end;

%else %if &pct. < -10 %then %do;

%let mu=&p_15.; /* if 15 or more percentile less than p0, then change to _15 */

data aftertrim; set aftertrim;

%if &modeltype.=1 %then %do;

APvar=(z)*(1/(1+exp(-(z-&mu.)/&tau.)));

%end;

%if &modeltype.=2 %then %do;

APvar= log(z)*(1/(1+exp(-(z-&mu.)/&tau.)));

%end;run;

%end;

%else %do;

%let mu=&&p&pct.;

data aftertrim; set aftertrim;

%if &modeltype.=1 %then %do;

APvar=(z)*(1/(1+exp(-(z-&mu.)/&tau.)));

%end;

%if &modeltype.=2 %then %do;

APvar= log(z)*(1/(1+exp(-(z-&mu.)/&tau.)));

%end;run;

%end;

/* non-linear model*/

%if &linear_model. eq 0 %then %do;

%if %length(&start.)=0 %then %do;

%if %length(&time.)=0 %then %do; %put "WARNING: TIME variable is missing"; %abort;%end;

%if %length(&strata.)=0 %then %do;

proc phreg data=aftertrim;

model &time.*&case.(0)=APvar &covvars./TIES=DISCRETE;

ods output FitStatistics=fits(where=(Criterion='-2 LOG L'))

ParameterEstimates=param (where=(parameter='APvar'));

run;

%end;

%else %do;

proc phreg data=aftertrim ;

model &time.*&case.(0)=APvar &covvars./TIES=DISCRETE;

strata &strata.;

ods output FitStatistics=fits(where=(Criterion='-2 LOG L'))

ParameterEstimates=param (where=(parameter='APvar'));

run;

%end;

%end;

%if %length(&start.)>0 %then %do;

%if %length(&stop.)=0 %then %do; %put "WARNING: STOP variable is missing"; %abort;%end;

%if %length(&strata.)=0 %then %do;

proc phreg data=aftertrim ;

model (&start.,&stop.)*&case.(0)=APvar &covvars./TIES=DISCRETE;

ods output FitStatistics=fits(where=(Criterion='-2 LOG L'))

ParameterEstimates=param (where=(parameter='APvar'));

run;

%end;

%else %do;

proc phreg data=aftertrim ;

model (&start.,&stop.)*&case.(0)=APvar &covvars./TIES=DISCRETE;

strata &strata.;

ods output FitStatistics=fits(where=(Criterion='-2 LOG L'))

ParameterEstimates=param (where=(parameter='APvar'));

run;

%end;

%end;

%end;

/* pure linear z model */

%if &linear_model. eq 1 %then %do;

%if %length(&start.)=0 %then %do;

%if %length(&time.)=0 %then %do; %put "WARNING: TIME variable is missing"; %abort;%end;

%if %length(&strata.)=0 %then %do;

proc phreg data=aftertrim ;

model &time.*&case.(0)=z &covvars./TIES=DISCRETE;

ods output FitStatistics=fits(where=(Criterion='-2 LOG L'))

ParameterEstimates=param (where=(parameter='z'));

run;

%end;

%else %do;

proc phreg data=aftertrim ;

model &time.*&case.(0)=z &covvars./TIES=DISCRETE;

strata &strata.;

ods output FitStatistics=fits(where=(Criterion='-2 LOG L'))

ParameterEstimates=param (where=(parameter='z'));

run;

%end;

%end;

%if %length(&start.)>0 %then %do;

%if %length(&stop.)=0 %then %do; %put "WARNING: STOP variable is missing"; %abort;%end;

%if %length(&strata.)=0 %then %do;

proc phreg data=aftertrim ;

model (&start.,&stop.)*&case.(0)=z &covvars./TIES=DISCRETE;

ods output FitStatistics=fits(where=(Criterion='-2 LOG L'))

ParameterEstimates=param (where=(parameter='z'));

run;

%end;

%else %do;

proc phreg data=aftertrim ;

model (&start.,&stop.)*&case.(0)=z &covvars./TIES=DISCRETE;

strata &strata.;

ods output FitStatistics=fits(where=(Criterion='-2 LOG L'))

ParameterEstimates=param (where=(parameter='z'));

run;

%end;

%end;

%end;

/* pure log(z) model */

%if &linear_model. eq 2 %then %do;

data new_datain; set aftertrim; ap_new=log(z); run;

%if %length(&start.)=0 %then %do;

%if %length(&time.)=0 %then %do; %put "WARNING: TIME variable is missing"; %abort;%end;

%if %length(&strata.)=0 %then %do;

proc phreg data=new_datain ;

model &time.*&case.(0)=ap_new &covvars./TIES=DISCRETE;

ods output FitStatistics=fits(where=(Criterion='-2 LOG L'))

ParameterEstimates=param (where=(parameter='ap_new'));

run;

%end;

%else %do;

proc phreg data=new_datain ;

model &time.*&case.(0)=ap_new &covvars./TIES=DISCRETE;

strata &strata.;

ods output FitStatistics=fits(where=(Criterion='-2 LOG L'))

ParameterEstimates=param (where=(parameter='ap_new'));

run;

%end;

%end;

%if %length(&start.)>0 %then %do;

%if %length(&stop.)=0 %then %do; %put "WARNING: STOP variable is missing"; %abort;%end;

%if %length(&strata.)=0 %then %do;

proc phreg data=new_datain ;

model (&start.,&stop.)*&case.(0)=ap_new &covvars./TIES=DISCRETE;

ods output FitStatistics=fits(where=(Criterion='-2 LOG L'))

ParameterEstimates=param (where=(parameter='ap_new'));

run;

%end;

%else %do;

proc phreg data=new_datain ;

model (&start.,&stop.)*&case.(0)=ap_new &covvars./TIES=DISCRETE;

strata &strata.;

ods output FitStatistics=fits(where=(Criterion='-2 LOG L'))

ParameterEstimates=param (where=(parameter='ap_new'));

run;

%end;

%end;

%end;

data param; set param; call symput('new_coef',Estimate); call symput('new_std',StdErr);run;

%if &linear_model. eq 0 %then %do;

data fits;set fits; length modeltype $4.; modeltype=&modeltype.; pct=&pct.; pctl=&mu.; coef=&new_coef.;

stderr=&new_std.;

run;

/* data fits;set fits; if modeltype=1 then pctl=pctl-&Tran.; run; */

%end;

%if &linear_model. eq 1 %then %do;

data fits;set fits; length modeltype $4.; modeltype=3; pct=.; pctl=.; coef=&new_coef.; stderr=&new_std.; run;

%end;

%if &linear_model. eq 2 %then %do;

data fits;set fits; length modeltype $4.; modeltype=4; pct=.; pctl=.; coef=&new_coef.; stderr=&new_std.; run;

%end;

/* first 8 model runs corresponding to tau=0.1 */

%if &model_tau.=1 %then %do;

%LET count=%SYSEVALF(&count. + 1);

data fits;set fits; iteration=&count.; run;

/* the very first model examined */

%if &count.=1 %then %do;

data dataout_1;set fits;run; %put "lastly here here here"; %put &count.; %end;

%else %do;

data dataout_1;set dataout_1 fits; run; %end;

%if &pct.<0 %then %do; %let abs_pct=%sysfunc(abs(&pct.)); data mtll_&modeltype._&abs_pct._;set fits; run;; %end;

%else %do; data mtll_&modeltype._&pct.;set fits; run; %end;

%end;

/* second 8 model runs corresponding to tau=0.2 */

%else %if &model_tau.=2 %then %do;

%LET count=%SYSEVALF(&count. + 1);

data fits;set fits; iteration=&count.; run;

/* the very first model examined */

%if &count.=1 %then %do;

data dataout_2;set fits;run; %put "lastly here here here"; %put &count.; %end;

%else %do;

data dataout_2;set dataout_2 fits; run; %end;

%if &pct.<0 %then %do; %let abs_pct=%sysfunc(abs(&pct.)); data mtll_&modeltype._&abs_pct._;set fits; run;; %end;

%else %do; data mtll_&modeltype._&pct.;set fits; run; %end;

%end;

/* continue the rest of model runs, based on selected tau, either 0.1 or 0.2 */

%else %if &model_tau.=9 %then %do;

%LET count=%SYSEVALF(&count. + 1);

data fits;set fits; iteration=&count.; run;

/* based on the selected tau, initiate output dataset*/

%if &count.=9 %then %do;

%if &set_tau.=0.1 %then %do;

data &dataout.;set dataout_1;run;

%end;

%else %if &set_tau.=0.2 %then %do;

data &dataout.;set dataout_2;run;

%end;

%end;

/* append the 9th model and onwards*/

%put "Append the 9th model and onwards";

data &dataout.;set &dataout. fits; run;

%if &pct.<0 %then %do; %let abs_pct=%sysfunc(abs(&pct.)); data mtll_&modeltype._&abs_pct._;set fits; run; %end;

%else %do; data mtll_&modeltype._&pct.;set fits; run; %end;

%end;

run;

%Mend modelpct;

/**************************

* Simulate z over 1000 realizations

**************************/

%macro simulate_z1(modeltype=, nn=);

/* based on range of trimmed and translated ap_data */

%if %upcase(&translate.)= YES or %upcase(&translate.)= Y %then %do;

%let max_ap = %SYSEVALF(&pctlr.-&pctll.+&Tran.);

data newap(keep = z_sim);

do i = &Tran. to &max_ap. by 0.1;

z_sim = i;

output;

end;

run;

%end;

%else %do;

data newap(keep = z_sim);

do i = &pctll. to &pctlr. by 0.1;

z_sim = i;

output;

end;

run;

%end;

/* if linear model, have the min of 0 */

%if &modeltype.=1 %then %do;

data newap; set newap;

z_sim=z_sim-&Tran.;

run;

%end;

/* prepare nn x _n_ matrix to store z_sim */

proc sort data=newap; by z_sim; run;

data newap; set newap; seqno = _n_ ; run;

proc transpose data=newap out=newap_wide prefix=C; /* col name is C1, C2, ..., Cxxx */

id seqno;

var z_sim;

run;

data newap2 (drop=_name_ i); set newap_wide; /* nn*num_z matrix */

do i=1 to &nn.;

output;

end;

run;

/* extract percentiles of z_sim */

proc sql noprint;

select max(z_sim) into: z_p100 from newap;

select min(z_sim) into: z_p0 from newap;

quit;

proc univariate data=newap noprint;

var z_sim;

output out=percentiles pctlpts= 0 to 100 by 5 pctlpre=z_p;

run;

data percentiles; set percentiles; z_p_5=&z_p0.-(z_p5-&z_p0.); run; /* add a variable z_p_5 denoting 5 pctl below z_p0*/

data percentiles; set percentiles; z_p_10=&z_p0.-(z_p10-&z_p0.); run; /* add a variable z_p_10 denoting 10 pctl below z_p0*/

data percentiles; set percentiles; z_p_15=&z_p0.-(z_p15-&z_p0.); run; /* add a variable z_p_15 denoting 15 pctl below z_p0*/

proc transpose data=percentiles out=percentiles;run;

%mend simulate_z1;

%macro simulate_z2(indata=, model=, over_lay=);

/* prepare z for use in simulation, varying depending on perc, set_tau, and funcform */

%if &model.="joint" %then %do;

/* all models examined */

%if %length(&over_lay.)>0 %then %do;

%if %upcase(&over_lay.)=ALL %then %do;

data temp_sim; set &indata.; run;

data temp_sim; set temp_sim; seqno = _n_; wt_final3=wt; run;

%end;

%else %if %upcase(&over_lay.) ne ALL %then %do;

data temp_sim; set &indata.; where best3>=1; run;

data temp_sim; set temp_sim; seqno = _n_; run;

%end;

proc sql noprint;

select max(seqno) into: num_models from temp_sim; /* total number of models examined */

quit;

%let counter=1;

%do k=1 %to &num_models.;

data _null_; set temp_sim;

call symput('wt_each',wt_final3); call symput('z_tau',tau);

call symput('z_mu',mu);call symput('mtype',modeltype);

where seqno=&k.;

run;

/* num of models */

%let N=%SYSEVALF(1000*&wt_each., floor);

%if &N.> 0 %then %do;

/* simulate ap data */

%simulate_z1(modeltype=&mtype., nn=&N.);

data _null_;set percentiles;

call symput(_name_,col1);

run;

proc sql noprint;

select max(seqno) into: num_z from newap; /* total number of z_sim data points */

quit;

%let xxx=&num_z.;

/* calc tau based on set_tau of each model run */

%LET z_tau_val=%SYSEVALF(&z_tau.*(&z_p100.-&z_p0.));

/*calc log transformed ap data*/

%if &z_mu.= -5 %then %do;

%let z_mu_val=&z_p_5.; /* if 5 percentile less than p0, then change to _5 */

data newap2; set newap2;

array CC{&xxx.} C1-C&xxx.;

%do i=1 %to &xxx.;

%if &mtype.=1 %then %do;

CC{&i.}=(CC{&i.})*(1/(1+exp(-(CC{&i.}-&z_mu_val.)/&z_tau_val.)));

%end;

%if &mtype.=2 %then %do;

CC{&i.}= log(CC{&i.})*(1/(1+exp(-(CC{&i.}-&z_mu_val.)/&z_tau_val.)));

%end;

%end;

run;

%end;

%else %if &z_mu. = -10 %then %do;

%let z_mu_val=&z_p_10.; /* if 10 percentile less than p0, then change to _10 */

data newap2; set newap2;

array CC{&xxx.} C1-C&xxx.;

%do i=1 %to &xxx.;

%if &mtype.=1 %then %do;

CC{&i.}=(CC{&i.})*(1/(1+exp(-(CC{&i.}-&z_mu_val.)/&z_tau_val.)));

%end;

%if &mtype.=2 %then %do;

CC{&i.}= log(CC{&i.})*(1/(1+exp(-(CC{&i.}-&z_mu_val.)/&z_tau_val.)));

%end;

%end;

run;

%end;

%else %if &z_mu. < -10 %then %do;

%let z_mu_val=&z_p_15.; /* if 15 or more percentile less than p0, then change to _15 */

data newap2; set newap2;

array CC{&xxx.} C1-C&xxx.;

%do i=1 %to &xxx.;

%if &mtype.=1 %then %do;

CC{&i.}=(CC{&i.})*(1/(1+exp(-(CC{&i.}-&z_mu_val.)/&z_tau_val.)));

%end;

%if &mtype.=2 %then %do;

CC{&i.}= log(CC{&i.})*(1/(1+exp(-(CC{&i.}-&z_mu_val.)/&z_tau_val.)));

%end;

%end;

run;

%end;

%else %do;

%let xx=&z_mu.;

%let z_mu_val=&&z_p&xx.;

%put &z_mu_val.;

data newap2; set newap2;

array CC{&xxx.} C1-C&xxx.;

%do i=1 %to &xxx.;

%if &mtype.=1 %then %do;

CC{&i.}=(CC{&i.})*(1/(1+exp(-(CC{&i.}-&z_mu_val.)/&z_tau_val.)));

%end;

%if &mtype.=2 %then %do;

CC{&i.}= log(CC{&i.})*(1/(1+exp(-(CC{&i.}-&z_mu_val.)/&z_tau_val.)));

%end;

%end;

run;

%end;

%if &counter.=1 %then %do;

data newap3; set newap2; run;

%end;

%else %do;

data newap3; set newap3 newap2; run;

%end;

%let counter=%SYSEVALF(&counter.+1);

%end; /* if &N.> 0 */

%end; /* do k=1 */

%end; /*if %length(&over_lay.)>0*/

%end; /*if &model.="joint"*/

/* optimal model */

%if &model.="optimal" %then %do;

data temp_sim2; set &indata.; where best3=1; run;

proc sort data=temp_sim2 NODUPKEY; by best3; run; /* in case last 2 models had same LL */

data _null_; set temp_sim2;

call symput('wt_each',wt_final3); call symput('z_tau',tau);

call symput('z_mu',mu);call symput('mtype',modeltype);

run;

/* num of models */

%let N=1000;

%simulate_z1(modeltype=&mtype., nn=&N.);

data _null_;set percentiles;

call symput(_name_,col1);

run;

proc sql noprint;

select max(seqno) into: num_z from newap; /* total number of z_sim data points */

quit;

%let xxx=&num_z.;

/* calc tau based on set_tau of each model run */

%LET z_tau_val=%SYSEVALF(&z_tau.*(&z_p100.-&z_p0.));

/* extract mu */

%if &z_mu.= -5 %then %do;

%let z_mu_val=&z_p_5.;

%end;

%else %if &z_mu.= -10 %then %do;

%let z_mu_val=&z_p_10.;

%end;

%else %if &z_mu.= -15 %then %do;

%let z_mu_val=&z_p_15.;

%end;

%else %do;

%let xx=&z_mu.;

%let z_mu_val=&&z_p&xx.;

%end;

data newap3; set newap2;

array CC{&xxx.} C1-C&xxx.;

%do i=1 %to &xxx.;

%if &mtype.=1 %then %do;

CC{&i.}=(CC{&i.})*(1/(1+exp(-(CC{&i.}-&z_mu_val.)/&z_tau_val.)));

%end;

%if &mtype.=2 %then %do;

CC{&i.}= log(CC{&i.})*(1/(1+exp(-(CC{&i.}-&z_mu_val.)/&z_tau_val.)));

%end;

%end;

run;

%end;

data newap3; set newap3; id=_n_; run;

data newap3; set newap3; where 1<=id<=1000; run;

%mend simulate_z2;

/**************************

* Simulate beta over 1000 realizations

**************************/

%macro simulate_beta(indata=, model=, over_lay=);

%put %upcase(&over_lay.);

%if &model.="joint" %then %do;

/* all models examined */

%if %length(&over_lay.)>0 and %upcase(&over_lay.)=ALL %then %do;

data temp_sim; set &indata.; run;

data temp_sim; set temp_sim; seqno = _n_; run;

proc sql noprint;

select max(seqno) into: num_models from temp_sim; /* total number of models examined */

quit;

%do k=1 %to &num_models.;

data _null_; set temp_sim;

call symput('wt_each',wt); call symput('coef_each',coef);call symput('std_each',stderr);

where seqno=&k.;

run;

%let N=%SYSEVALF(1000*&wt_each., floor);

data sample_&k.(keep=beta);

call streaminit(4321);

do i=1 to &N.;

beta=rand("Normal", &coef_each., &std_each.);

output;

end;

run;

%if &k.=1 %then %do;

data sample_all; set sample_&k.; run;

%end;

%else %do;

data sample_all; set sample_all sample_&k.; run;

%end;

%end;

data simdata; set sample_all; run;

data simdata; set simdata; id=_n_; run;

%end;

/* 3 best models examined */

%if %length(&over_lay.)>0 and %upcase(&over_lay.) ne ALL %then %do;

data temp_sim; set &indata.; where best3>=1; run;

data temp_sim; set temp_sim; seqno = _n_; run;

proc sql noprint;

select max(seqno) into: num_models from temp_sim; /* total number of models examined */

quit;

/* first model */

data _null_; set temp_sim;

call symput('wt_each',wt_final3); call symput('coef_each',coef);call symput('std_each',stderr);

where seqno=1;

run;

%let N=%SYSEVALF(1000*&wt_each., floor);

data sample1(keep=beta);

call streaminit(4321);

do i=1 to &N.;

beta=rand("Normal", &coef_each., &std_each.);

output;

end;

run;

/* second model */

data _null_; set temp_sim;

call symput('wt_each',wt_final3); call symput('coef_each',coef);call symput('std_each',stderr);

where seqno=2;

run;

%let N=%SYSEVALF(1000*&wt_each., floor);

data sample2(keep=beta);

call streaminit(4321);

do i=1 to &N.;

beta=rand("Normal", &coef_each., &std_each.);

output;

end;

run;

/* third model */

data _null_; set temp_sim;

call symput('wt_each',wt_final3); call symput('coef_each',coef);call symput('std_each',stderr);

where seqno=3;

run;

%let N=%SYSEVALF(1000*&wt_each., floor);

data sample3(keep=beta);

call streaminit(4321);

do i=1 to &N.;

beta=rand("Normal", &coef_each., &std_each.);

output;

end;

run;

data sample_all; set sample1; run;

data sample_all; set sample_all sample2; run;

data sample_all; set sample_all sample3; run;

data simdata; set sample_all; run;

data simdata; set simdata; id=_n_; run;

%end;

%end;

/* optimal model */

%if &model.="optimal" %then %do;

data temp_sim2; set &indata.; where best3=1; run;

proc sort data=temp_sim2 NODUPKEY; by best3; run; /* in case last 2 models had same LL */

data _null_; set temp_sim2;

call symput('wt_each',wt_final3); call symput('coef_each',coef); call symput('std_each',stderr);

run;

%let N=1000;

data sample(keep=beta);

call streaminit(4321);

do i=1 to &N.;

beta=rand("Normal", &coef_each., &std_each.);

output;

end;

run;

data sample_all; set sample; run;

data simdata; set sample_all; id=_n_; run;

%end;

%mend simulate_beta;

/**************************

* plot c-r function

**************************/

%macro plot_cr(indata_joint=, indata_optimal=, expo=, unit=, over_lay=);

%if %length(&expo.)=0 %then %do;

%let label2='Air pollution concentration';

%end;

%else %do;

%IF %length(&unit.)>0 %then %do; %let label2="&expo. concentration (&unit.)"; %end;

%IF %length(&unit.)=0 %then %do; %let label2="&expo. concentration"; %end;

%end;

%put &label2.;

%put &unit.;

%if %length(&over_lay.)>0 %then %do;

data &indata_joint.; set &indata_joint.; model="Ensemble"; run;

data &indata_optimal.; set &indata_optimal.; model="Optimal"; run;

data combined; set &indata_joint. &indata_optimal.; run;

ods listing close;

ods html image_dpi=200 file='fitmodel.html' path="&output_path." style=listing;

ods graphics / reset noborder width=600px height=400px imagename="Nonlinear_&sysdate.";

title1 "Nonlinear C-R Curve";

proc sgplot data=combined;

series x=ap y=rr_mean / group=model;

band x=ap lower=rr_lcl upper=rr_ucl / group=model transparency=0.75;

xaxis label=&label2.;

yaxis label='Odds Ratio (95% CI)';

refline 1;

keylegend / location=inside position=topleft across=1;

run;

ods html close;

ods listing;

%end;

%else %do;

data &indata_optimal.; set &indata_optimal.; model="Optimal"; run;

data combined; set &indata_optimal.; run;

ods listing close;

ods html image_dpi=200 file='fitmodel.html' path="&output_path." style=listing;

ods graphics / reset noborder width=600px height=400px imagename="Nonlinear_&sysdate.";

title1 "Nonlinear C-R Curve";

proc sgplot data=combined;

series x=ap y=rr_mean / group=model;

band x=ap lower=rr_lcl upper=rr_ucl / group=model transparency=0.75;

xaxis label=&label2.;

yaxis label='Odds Ratio (95% CI)';

refline 1;

keylegend / location=inside position=topleft across=1;

run;

ods html close;

ods listing;

%end;

%mend plot_cr;

**********************************************************************************************************************************************************************************************The end SAS******************************************************

Conditional logistic

##########################################################################################################################################################################

###################################R#####################################

########################################################################

########################################################################

##

## Nonlinear concentraton-response function: model fitting and plotting procedure

##

## Date: Feb 24, 2016

## Version 2.10

##

## Purpose: This program will fit a series of nonlinear concentration-response functions using

## conditional logistic regression modeling to identify optimal nonlinear relationship

##

## It produces 3 outputs: (1) a graph showing nonlinear relationship based on an optimal model and an ensemble model,

## (2) a summary table listing coefficient, standard error, loglik, and function form of each cox model that was examined, and

## (3) a descriptive table showing distribution of air pollution exposure variable in the origianl and trimmed datasets

##

##

## For more details about this modeling approach, please refer to the accompanying paper Masoud, Szyszkowicz,...,Burnett et al 2016 Air Quality, Atmosphere and Health

##

## Should there be any question with this program, please contact:

## Drs Hong Chen (Hong.Chen@oahpp.ca) and Rick Burnett (Rick.Burnett@hc-sc.gc.ca)

##

##

## Please read the following notes before running the program

##

## 1. All categorical variables should be defined as factor variables before calling bestcox().

## To do this, you may use, for example, inputdata$education <- as.factor(inputdata$education).

##

## 2. When calling bestcox(), you need to define parameters according to the column names of your input dataset.

##

## 3. Your input dataset should be a dataframe. Also need to specify status of 1=case 0=control.

##

## 4. By default, the plot will display a nonlinear curve based on optimal model only.

## This can be done either by leaving out overlay parameter in the bestcox() or explicitly define overlay=NA.

##

## To overlay ensemble model based on the 3 best models examined, define overlay="best3" in the bestcox().

##

## To overlay ensemble model based on all models examined, define overlay="all" in the bestcox().

##

## 5. By default, the bestcox() will translate air pollution exposure variable such that it has a minimum of unity.

##

## If users do not wish to translate data, you need to specify translate=F or FALSE

##

## 6. By default, output_dir is set as R's default working directory

##

## If users wish to specify a different output directory, you need to ensure to have write permission

##

##

## To illustrate how to use this routine, 4 examples are given below:

##

## Example 1: (without trimming and translating air pollution variable, with 1 strata variable,

## and overlay ensemble model based on 3 best models)

##

## out <- bestcox(data = inputdata, translate=F, casename = "case", lowperc = 0, upperc = 100,

## expo_name = "no2", cova_name = c("age","sex"), strata_name= c("match_id"), expo_unit = "ppb", overlay=3,

## output_dir="F:\\your output directory\\")

## out

##

## Example 2: (with trimmed and translated air pollution variable, with 1 strata variable,

## and overlay ensemble model based on 3 best models)

##

## out <- bestcox(data = inputdata, translate=T, casename = "death", lowperc = 1, upperc = 99,

## expo_name = c("pm25"), cova_name = c("age", "sex"), strata_name= c("match_id"), expo_unit = c("ug/m3"), overlay="best3",

## output_dir="F:\\your output directory\\")

## out

##

## Example 3: (without trimmming and translated air pollution variable, with 2 strata variables,

## and overlay ensemble model based on all models examined)

##

## out <- bestcox(data = inputdata, translate=F, casename = "case", lowperc = 0, upperc = 100,

## expo_name = c("no2"), cova_name = c("age"), strata_name= c("match_id", "sex"), expo_unit = c("ppb"), overlay="all",

## output_dir="F:\\your output directory\\")

## out

##

## Example 4: (with trimmed and translated air pollution variable, with 2 strata variables, and only show optimal model)

##

## out <- bestcox(data = inputdata, translate=T, casename="case", lowperc = 1, upperc = 99,

## expo_name="no2", cova_name = c("sex", "income", "bmi"), strata_name= c("match_id", "age"), expo_unit = c("ppb"), overlay=NA,

## output_dir="F:\\your output directory\\")

## out

##

##

########################################################################

#########################################################

## function to search for the optimal nonlinear model

#########################################################

library(survival)

library(MASS)

bestcox <- function(data,translate=TRUE,start=NA,end=NA,casename,lowperc,upperc,expo_name,cova_name,strata_name=NA,expo_unit,overlay=NA,output_dir=NA){

data$ap <- data[,expo_name]

data$ap[data$ap<1] <- 1 # convert any conc < 1 to 1

# Characterize the distribution of air pollution exposure variable in the original dataset

size_original <- length(data$ap)

summary_original <- summary(data$ap)

# Trim data, data translation, and tau

data_trim <- subset(data, ap <= quantile(data$ap,upperc/100,na.rm = T) & ap >= quantile(data$ap, lowperc/100, na.rm = T))

rm(data) # remove original dataset to save memory space

if (translate){

ap.min <- min(data_trim$ap, na.rm = T) # translate AP

tran <- 1

} else {

ap.min <- 0 # no translate

tran <- 0

}

data_trim$ap_trans <- data_trim$ap - ap.min + tran # translate z to have the min of 1

data_trim$ap_trans_lm <- data_trim$ap - ap.min # translate z to have the min of 0

data_trim$ap_trans_log <- log(data_trim$ap_trans)

set_tau <- NA

if (length(set_tau)==0 || any(is.na(set_tau)) || set_tau=='') {

# set_tau <- 0.1

tau.2 <- 0.1*(max(data_trim$ap_trans, na.rm = T) - min(data_trim$ap_trans, na.rm = T))

# set_tau <- 0.2

tau.2[2]<- 0.2*(max(data_trim$ap_trans, na.rm = T) - min(data_trim$ap_trans, na.rm = T))

num_tau <- 2

}else{

tau <- set_tau*(max(data_trim$ap_trans, na.rm = T) - min(data_trim$ap_trans, na.rm = T))

num_tau <- 1

}

step_history <- NULL

# Create a function to extract LL from Conditional logistic regression model

coxmodel <- function(funcform, loca_perc, method = "exact"){

# Create f(z)*Wt

if (loca_perc < 0){

loca_para <- min(data_trim$ap_trans, na.rm = T) + (min(data_trim$ap_trans, na.rm = T) - quantile(data_trim$ap_trans, abs(loca_perc)/100, na.rm = T))

loca_para_lm <- min(data_trim$ap_trans_lm, na.rm = T) + (min(data_trim$ap_trans_lm, na.rm = T) - quantile(data_trim$ap_trans_lm, abs(loca_perc)/100, na.rm = T)) # translate z to have the min of 0

}else{

loca_para <- quantile(data_trim$ap_trans, loca_perc/100, na.rm = T)

loca_para_lm <- quantile(data_trim$ap_trans_lm, loca_perc/100, na.rm = T) # translate z to have the min of 0

}

logit_w <- 1/(1+exp(-(data_trim$ap_trans - loca_para)/tau))

logit_w_lm <- 1/(1+exp(-(data_trim$ap_trans_lm - loca_para_lm)/tau)) # translate z to have the min of 0

capture_mu <- NA # capture mu

if (funcform == "linear"){

data_trim$expo <- logit_w_lm * data_trim$ap_trans_lm # translate z to have the min of 0

capture_mu <- loca_para_lm # capture mu

}

if (funcform == "log"){

data_trim$expo <- logit_w * data_trim$ap_trans_log

capture_mu <- loca_para # capture mu

}

if (funcform == "pure.linear"){

data_trim$expo <- data_trim$ap_trans_lm # translate z to have the min of 0

capture_mu <- NA # capture mu

}

if (funcform == "pure.log"){

data_trim$expo <- data_trim$ap_trans_log

capture_mu <- NA # capture mu

}

rm(logit_w) # remove logit_w to save memory space

rm(logit_w_lm) # remove logit_w_lm to save memory space

# Create clogit formula

## without strata variable

if (length(strata_name)==0 || any(is.na(strata_name)) || strata_name=='') {

stop("Program halted - please specify a strata variable(s) in order to run conditional logistic regression model.")

## with strata variable

} else {

coxformula <- paste(casename,"~expo+",paste(cova_name,collapse="+"),"+","strata(",paste(strata_name, collapse=","),")",sep="")

}

# Call clogit

coxfit <- clogit(as.formula(coxformula), data = data_trim, method = method)

est <- summary(coxfit)$coefficients[c("expo"),c("coef","se(coef)")]

result <- data.frame(coef = est[1], se.coef=est[2], LL = coxfit$loglik[2], mu=capture_mu) # capture mu

rm(coxfit) # remove coxfit to save memory space

return(result)

} # end of clogit model

# Transformation Form Selection (8 or 16 Models, depending if users specify tau)

if (length(set_tau)==0 || any(is.na(set_tau)) || set_tau=='') {

tau <- tau.2[1] # tau=0.1

step_a <- data.frame(funcform = c(rep("linear",4),rep("log",4)), loca_perc = rep(c(0,25,50,75),2),

coef = NA, se.coef = NA, LL = NA, mu=NA)

for (i in 1:nrow(step_a)){

step_a[i,c("coef","se.coef","LL", "mu")] <- coxmodel(step_a$funcform[i], step_a$loca_perc[i])

}

step_a$tau <- tau

LL.step_a <- step_a[step_a$LL == max(step_a$LL,na.rm = T),]$LL

tau <- tau.2[2] # tau=0.2

step_b <- data.frame(funcform = c(rep("linear",4),rep("log",4)), loca_perc = rep(c(0,25,50,75),2),

coef = NA, se.coef = NA, LL = NA, mu=NA)

for (i in 1:nrow(step_b)){

step_b[i,c("coef","se.coef","LL", "mu")] <- coxmodel(step_b$funcform[i], step_b$loca_perc[i])

}

step_b$tau <- tau

LL.step_b <- step_b[step_b$LL == max(step_b$LL,na.rm = T),]$LL

if (LL.step_a > LL.step_b){

step_0 <- step_a

rejected.models <- step_b # retain rejected tau and related model outputs

rejected.tau <- step_b[1,]$tau # retain rejected tau and related model outputs

}else{

step_0 <- step_b

rejected.models <- step_a # retain rejected tau and related model outputs

rejected.tau <- step_a[1,]$tau # retain rejected tau and related model outputs

}

}else{

step_0 <- data.frame(funcform = c(rep("linear",4),rep("log",4)), loca_perc = rep(c(0,25,50,75),2),

coef = NA, se.coef = NA, LL = NA, mu=NA)

for (i in 1:nrow(step_0)){

step_0[i,c("coef","se.coef","LL", "mu")] <- coxmodel(step_0$funcform[i], step_0$loca_perc[i])

}

step_0$tau <- tau

}

step_history <- step_0

step_history <- subset(step_history, select=-c(tau))

step_0 <- step_0[step_0$LL == max(step_0$LL,na.rm = T),]

funcform <- as.character(step_0$funcform)

tau <- step_0$tau # define best tau

set_tau <- tau/(max(data_trim$ap_trans, na.rm = T) - min(data_trim$ap_trans, na.rm = T)) # best set_tau (0.1 or 0.2)

step_0 <- subset(step_0, select=-c(tau)) # drop tau

set_tau_rejected <- rejected.tau/(max(data_trim$ap_trans, na.rm = T) - min(data_trim$ap_trans, na.rm = T)) # rejected set_tau

# Transformation Location Parameter Selection

#para0 <- step_0$loca_perc

#

#if(para0 == 25){

# step_temp <- cbind(funcform, loca_perc = 0 ,as.vector(coxmodel(funcform, 0)))

# step_0 <- rbind(step_0, step_temp)

# step_history <- rbind(step_history, step_temp)

#}else if(para0 == 50){

# step_temp <- cbind(funcform, loca_perc = 75 ,as.vector(coxmodel(funcform, 75)))

# step_0 <- rbind(step_0, step_temp)

# step_history <- rbind(step_history, step_temp)

#}

#step_0 <- step_0[step_0$LL == max(step_0$LL,na.rm = T),]

para0 <- as.numeric(step_0$loca_perc)

step_temp <- rbind(cbind(funcform, loca_perc = (para0 + 5) ,as.vector(coxmodel(funcform, para0 + 5))),

cbind(funcform, loca_perc = (para0 - 5) ,as.vector(coxmodel(funcform, para0 - 5))))

step_0 <- rbind(step_0, step_temp)

step_history <- rbind(step_history, step_temp)

step_1 <- step_0[step_0$LL == max(step_0$LL,na.rm = T),]

para1 <- as.numeric(step_1$loca_perc)

# STOP if reaching 15% below mu=1 or LL is not smaller

##while(!(para1 %in% c(-5,100,para0))){

while(!(para1 %in% c(-15,100,para0))){

para_temp <- para1 + (para1 - para0)

para0 <- para1

para1 <- para_temp

step_temp <- cbind(funcform, loca_perc = para1 ,as.vector(coxmodel(funcform, para1)))

step_0 <- rbind(step_1, step_temp)

step_history <- rbind(step_history, step_temp)

step_1 <- step_0[step_0$LL == max(step_0$LL,na.rm = T),]

para1 <- as.numeric(step_1$loca_perc)

}

# Reformat step_history and add back rejected model outputs

rownames(step_history) <- NULL

step_history$tau <- set_tau # define best tau (0.1 or 0.2)

step_history_1to8 <- step_history[1:8,]

step_history_9tolast <- step_history[9:length(step_history[,1]),]

rejected.models$tau <- set_tau_rejected # define set_tau_rejected (0.2 or 0.1)

step_history_1to8 <- rbind(step_history_1to8, rejected.models)

step_history <- rbind(step_history_1to8, step_history_9tolast) # re populate "step_history"

# Add ensemble weights for 3 models around the best fit, ie., based on best mu with + and - 5th percentile

step_history_sort3 <- step_history

rownames(step_history_sort3) <- NULL # re start row number

bestLL <- max(step_history_sort3$LL)

step_history_sort3$best3 <- ifelse(step_history_sort3$LL==bestLL, 1, 0)

step_history_sort3$iteration <- rownames(step_history_sort3)

best.models <- step_history_sort3[step_history_sort3$best3==1,]

best.models.sort <- best.models[order(best.models$iteration),]

best.final.LL.iteration <- best.models.sort[length(best.models.sort[,1]),]$iteration

final.mu <- best.models.sort[length(best.models.sort[,1]),]$loca_perc

best.model.form <- best.models.sort[length(best.models.sort[,1]),]$funcform

if (sum(step_history_sort3$best3)==2) {

if (length(step_history_sort3[,1]) > best.final.LL.iteration){

step_history_sort3[length(step_history_sort3[,1]),]$best3 <- 3

} else if (length(step_history_sort3[,1]) == best.final.LL.iteration) {

sec.final.mu <- best.models.sort[(length(best.models.sort[,1])-1),]$loca_perc

if (final.mu > sec.final.mu) {

step_history_sort3[step_history_sort3$loca_perc==(final.mu-10) & step_history_sort3$funcform==best.model.form,]$best3 <- 3

} else {

step_history_sort3[step_history_sort3$loca_perc==(final.mu+10) & step_history_sort3$funcform==best.model.form,]$best3 <- 3

}

}

} else if (sum(step_history_sort3$best3)==1) {

##if (final.mu==-5) {

## step_history_sort3[step_history_sort3$loca_perc==0 & step_history_sort3$funcform==best.model.form,]$best3 <- 2

## step_history_sort3[step_history_sort3$loca_perc==5 & step_history_sort3$funcform==best.model.form,]$best3 <- 3

if (final.mu==-15) {

step_history_sort3[step_history_sort3$loca_perc==-10 & step_history_sort3$funcform==best.model.form,]$best3 <- 2

step_history_sort3[step_history_sort3$loca_perc==-5 & step_history_sort3$funcform==best.model.form,]$best3 <- 3

} else if (final.mu==100) {

step_history_sort3[step_history_sort3$loca_perc==95 & step_history_sort3$funcform==best.model.form,]$best3 <- 2

step_history_sort3[step_history_sort3$loca_perc==90 & step_history_sort3$funcform==best.model.form,]$best3 <- 3

} else {

step_history_sort3[step_history_sort3$loca_perc==(final.mu-5) & step_history_sort3$funcform==best.model.form,]$best3 <- 2

step_history_sort3[step_history_sort3$loca_perc==(final.mu+5) & step_history_sort3$funcform==best.model.form,]$best3 <- 3

}

}

# in rare occasion, models with set_tau_rejected may be assigned best3=2 or 3, thus need to be assigned to 0

step_history_sort3[step_history_sort3$tau==set_tau_rejected,]$best3 <-0

nn <- length(step_history_sort3[step_history_sort3$best3>=1,]$LL) # number of best fit models, normally this should be 3

step_history_sort3$LL.diff <- NA

step_history_sort3[step_history_sort3$best3>=1,]$LL.diff <- exp(step_history_sort3[step_history_sort3$best3>=1,]$LL - min(step_history_sort3[step_history_sort3$best3>=1,]$LL))

step_history_sort3$wt.final3 <- NA

step_history_sort3[step_history_sort3$best3>=1,]$wt.final3 <- step_history_sort3[step_history_sort3$best3>=1,]$LL.diff / sum(step_history_sort3[step_history_sort3$best3>=1,]$LL.diff, na.rm = T)

step_history_sort3<-subset(step_history_sort3, select =-c(LL.diff, best3, iteration))

if (nn >= 1) {

step_history_sort <- step_history_sort3

} else {

step_history_sort <- step_history

step_history_sort$wt <- NA

step_history_sort$wt.final3 <- NA

}

# Add ensemble weights for all the models

step_history_sort$wt <- exp(step_history_sort$LL-min(step_history_sort$LL))/sum(exp(step_history_sort$LL-min(step_history_sort$LL)))

rownames(step_history_sort) <- NULL

# Plot

if (nn >= 1) {

finalmodels.best.nn <- subset(step_history_sort, !is.na(wt.final3)) ## by default, limit to the best nn=3 models

finalmodels.best.nn.final <- finalmodels.best.nn[order(finalmodels.best.nn$LL),]

##if overlay==ALL then ensemble model would be global all models examined

if (!is.na(overlay) & toupper(as.character(overlay))=='ALL'){

nn <- length(step_history_sort[,1])

step_history_sort.nn <- step_history_sort

step_history_sort.nn$wt.final3 <- step_history_sort$wt

finalmodels.best.nn.final <- step_history_sort.nn[order(step_history_sort.nn$LL),]

}

##result <- try(plot.bestmodel(ap_data=data_trim$ap, finalmodels=finalmodels.best.nn.final, expo_name=expo_name, unit=expo_unit, nn=nn))

result <- try(plot.bestmodel(ap_data=data_trim$ap, finalmodels=finalmodels.best.nn.final, expo_name=expo_name, unit=expo_unit, nn=nn, overlay=overlay, set_tau=set_tau, set_tau_reject=set_tau_rejected, tran=tran, translate=translate))

if (class(result)=="try-error") {

print("Unable to find joint model! Plot is suppressed and only summary table is produced.")

next

}

} else {

print("Unable to find optimal model! Plot is suppressed and only summary table is produced.")

}

# Pure linear z model: exp(beta*z) (note that loca_perc = 75 is only a place holder)

step_pure_linear <- cbind(funcform="pure.linear", loca_perc = 75 ,as.vector(coxmodel(funcform="pure.linear", 75)))

step_pure_linear$loca_perc <- NA

step_pure_linear$tau <- NA # NA for tau

step_pure_linear$wt.final3 <- NA

step_pure_linear$wt <- NA

rownames(step_pure_linear) <- NULL

# Pure log(z) model: exp(beta*log(z)) (note that loca_perc = 75 is only a place holder)

step_pure_log <- cbind(funcform="pure.log", loca_perc = 75 ,as.vector(coxmodel(funcform="pure.log", 75)))

step_pure_log$loca_perc <- NA

step_pure_log$tau <- NA # NA for tau

step_pure_log$wt.final3 <- NA

step_pure_log$wt <- NA

rownames(step_pure_log) <- NULL

# Output summary table from each step including coef, std, and wt

step_history_sort <- rbind(step_history_sort,step_pure_linear)

step_history_sort <- rbind(step_history_sort,step_pure_log)

##colnames(step_history_sort)[colnames(step_history_sort)=="loca_perc"] <- "location"

colnames(step_history_sort)[2] <- "location"

colnames(step_history_sort)[4] <- "se"

colnames(step_history_sort)[8] <- "finalwt"

# re-order summary table

step_history_sort_final <- step_history_sort[,c("funcform","location","mu","tau","coef","se","LL","wt","finalwt")]

step_history_sort_final$LL <- format(round(step_history_sort_final$LL, 5), nsmall = 6) ## show 6 decimals of LL

#return(step_history_sort_final)

#if (num_tau == 1) {

# step_history_sort_final$tau <- set_tau

#}else{

#step_history_sort_final$tau <- NA

#step_history_sort_final[1:8,]$tau <- 0.1

#step_history_sort_final[9:16,]$tau <- 0.2

#step_history_sort_final[17:length(step_history_sort_final[,1])-2,]$tau <- set_tau

# step_history_sort_final$tau <- set_tau

#}

write.table(step_history_sort_final, file=paste(output_dir, "search.results.csv", sep=""), sep = ",", col.names = NA)

# add 2 summary tables showing the distribution of air pollution exposure variable in the original and trimmed datasets, respectively

size_trimmed <- length(data_trim$ap)

summary_trimmed <- summary(data_trim$ap)

overall_summary <- list("count_of_obs_in_original_dataset"=size_original,

"distr_of_exp_in_original_dataset"=summary_original,

"count_of_obs_in_trimmed_dataset"=size_trimmed,

"distr_of_exp_in_trimmed_dataset"=summary_trimmed,

"summary_model_fitting"=step_history_sort_final)

# output all 3 summary tables: (1) summary of model fitting and (2) distr of exp var in both original and trimmed datasets

return(overall_summary)

}

#########################################################

## function to plot nonlinear CR relationship

#########################################################

plot.bestmodel <- function(ap_data, finalmodels, expo_name, unit, nn, overlay, set_tau, set_tau_reject, tran, translate){

x <- seq(tran, max(ap_data)-min(ap_data)+tran, 0.1) # based on range of trimmed and translated ap_data

nx <- length(x)

if (finalmodels[length(finalmodels[,1]),]$funcform == "linear") {

x <- x-tran # translate z to have the min of 0

}

if (translate) {

x <- x

} else {

x <- seq(min(ap_data), max(ap_data), 0.1) # if translate=FALSE, use full range of original AP data

nx <- length(x)

}

# prepare x1 for use in simulation, varying depending on perc, set_tau, and funcform

sim.x1 <- function(x_sim, perc, set_tau_sim, funcform_sim){

if (perc < 0){

mu <- min(x_sim, na.rm = T) + (min(x_sim, na.rm = T)-quantile(x_sim, abs(perc)/100, na.rm = T))

}else{

mu <- quantile(x_sim, perc/100, na.rm = T)

}

tau_sim <- set_tau_sim*(max(x_sim)-min(x_sim))

logit <- exp((x_sim-mu)/tau_sim)/(1+exp((x_sim-mu)/tau_sim))

if (funcform_sim== "linear"){

x1<-x_sim*logit

}

if (funcform_sim == "log"){

x1<-log(x_sim)*logit

}

# x1 <- x1-min(x1) # set min(x1) as reference, thus beta*min(x1)=0

return(x1)

}

# Consider only optimal model - simulate 1000 realizations based on se.coef alone

nsim<-1000

ran<-matrix(0, nsim, 1)

rr<-matrix(0, nsim, nx)

medRR<-matrix(0, nx, 1)

upcl<-matrix(0, nx, 1)

lowcl<-matrix(0, nx, 1)

loca_perc_sim <- finalmodels[finalmodels$LL==max(finalmodels$LL, na.rm=T),]$loca_perc

funcform_sim <- finalmodels[length(finalmodels[,1]),]$funcform

x1 <- sim.x1(x_sim=x, perc=loca_perc_sim, set_tau_sim=set_tau, funcform_sim=funcform_sim)

for (i in 1:nsim) {

ran[i,]<-rnorm(1, finalmodels[length(finalmodels[,1]),]$coef, finalmodels[length(finalmodels[,1]),]$se.coef)

for (j in 1:length(x)) {

rr[i,j]<-exp(ran[i,1]*x1[j])

}

}

for (j in 1:length(x)) {

medRR[j] <- mean(rr[,j])

lowcl[j] <- quantile(rr[,j], 0.025)

upcl[j] <- quantile(rr[,j], 0.975)

}

# Incorporate 3 best models or ALL models - simulate 1000 realizations based on se.coef AND weights derived from LL

if (nn >= 2) {

nsim<-1000

ran.3<-matrix(0, nsim, 1)

rr.3<-matrix(0, nsim, nx)

medRR.3<-matrix(0, nx, 1)

upcl.3<-matrix(0, nx, 1)

lowcl.3<-matrix(0, nx, 1)

pp <- 1 # position variable in the 1000 sim

nn <- nn # consider top 3 models

nsim.sum <- 0 # count of nsim to ensure the last run lead to rownum of exactly 1000

# k from 0 to nn-1: varying depending on models included and pooled

for (k in 0:(nn-1)) {

nsim.wt <- nsim * round(finalmodels[length(finalmodels[,1])-k,]$wt.final3, digits = 3)

loca_perc_sim <- finalmodels[length(finalmodels[,1])-k,]$loca_perc

funcform_sim <- finalmodels[length(finalmodels[,1])-k,]$funcform

if (finalmodels[length(finalmodels[,1])-k,]$tau==set_tau_reject){

x1 <- sim.x1(x_sim=x, perc=loca_perc_sim, set_tau_sim=set_tau_reject, funcform_sim=funcform_sim)

}else{

x1 <- sim.x1(x_sim=x, perc=loca_perc_sim, set_tau_sim=set_tau, funcform_sim=funcform_sim)

}

if (k==nn-1) {nsim.wt <- nsim - nsim.sum}

for (i in pp:(pp+nsim.wt-1)) {

# for models with weights~0, i may exceed 1000 thus throw an error on out of bound

if (i<=1000){

ran.3[i,]<-rnorm(1, finalmodels[length(finalmodels[,1])-k,]$coef, finalmodels[length(finalmodels[,1])-k,]$se.coef)

for (j in 1:length(x)){

rr.3[i,j]<-exp(ran.3[i,1]*x1[j])

}

}

}

pp <- pp+nsim.wt

nsim.sum <- nsim.sum+nsim.wt

}

for (j in 1:length(x)) {

medRR.3[j] <- mean(rr.3[,j])

lowcl.3[j] <- quantile(rr.3[,j], 0.025)

upcl.3[j] <- quantile(rr.3[,j], 0.975)

}

}

#transform x back to original scale of AP data for plotting

if (translate) {

if (finalmodels[length(finalmodels[,1]),]$funcform == "linear") {

x <- x+min(ap_data) # translate z to have the min of 0

} else {

x <- x+min(ap_data)-tran # translate z to have the min of 1

}

}

##whether or not overlay ensemble model

if (length(overlay)==0 || any(is.na(overlay)) || overlay==''){

# plot optimal model only

par(las = 1 , cex = 1)

# use predicted values from optimal model for the plot

medRR.3 <- medRR

lowcl.3 <- lowcl

upcl.3 <- upcl

plot(x, upcl.3, lwd=4, type="l", col="#DEEBF7", frame.plot=T, ylim=c(min(lowcl.3)-0.25, max(upcl.3)+0.25), ylab="Odds Ratio", xlab=paste(toupper(expo_name), " (", unit, ")"))

polygon(x=c(x, rev(x)), y=c(lowcl.3, rev(upcl.3)), col="#DEEBF7", border=NA, lty=2)

lines(x, medRR.3, lwd=3, col="#08519C")

lines(x, lowcl.3, lwd=2, col="#DEEBF7")

nn <- 1000

} else {

# plot joint model

par(las = 1 , cex = 1)

if (nn < 2) {

# if only optimal model exists, use predicted values from the optimal model for the plot

medRR.3 <- medRR

lowcl.3 <- lowcl

upcl.3 <- upcl

}

# in rare occasion, max(upcl.3)=INF

upcl.3.excluded.inf <- upcl.3[upcl.3<100]

max.ylim <- max(upcl.3.excluded.inf, na.rm=TRUE)+0.25

if (is.na(max.ylim)){max.ylim <- 5}

lowcl.3.excluded.inf <- lowcl.3[lowcl.3<100]

min.ylim <- min(lowcl.3.excluded.inf, na.rm=TRUE)-0.25

if (is.na(min.ylim)){min.ylim <- 0}

plot(x, upcl.3, lwd=4, type="l", col="#DEEBF7", frame.plot=T, ylim=c(min.ylim, max.ylim), ylab="Odds Ratio", xlab=paste(toupper(expo_name), " (", unit, ")"))

polygon(x=c(x, rev(x)), y=c(lowcl.3, rev(upcl.3)), col="#DEEBF7", border=NA, lty=2)

lines(x, medRR.3, lwd=3, col="#08519C")

lines(x, lowcl.3, lwd=2, col="#DEEBF7")

# Overlay optimal model

lines(x, medRR, lwd=2, col="red")

lines(x, lowcl, lwd=1, lty=2, col="red")

lines(x, upcl, lwd=1, lty=2, col="red")

}

# Add rugs, legend, and reference line

## add rugs=ticks at datapoints

####axis(side = 1 , line = -1.2 , at = jitter(x) , labels = F , tick = T , tcl = 0.8 , lwd.ticks = 0.1 , lwd = 0)

## rugs and labels at 1Q, median and 3Q

####axis(side = 1 , line = -1.0 , at = fivenum(x)[2:4], lwd = 0 , tick = T, tcl = 1.2 , lwd.ticks = 1 , col.ticks = "black" , labels = c("Quartile 1","Median","Quartile 3"), cex.axis = 0.7, col.axis = "black" , padj = -2.8)

####axis(side = 1 , line = 0.0 , at = fivenum(x)[2:4], lwd = 0 , tick = T, tcl = 0.2 , lwd.ticks = 1 , col.ticks = "black", labels = FALSE)

## add legend and RR=1 line

if (nn > 100) {

legend("topleft", inset=c(0,0), c("optimal model"), col=c("blue"), lty=1, lwd=3)

} else if (nn >= 2) {

legend("topleft", inset=c(0,0), c("ensemble model", "optimal model"), col=c("blue", "red"), lty=1, lwd=3)

} else {

legend("topleft", inset=c(0,0), c("optimal model", "optimal model"), col=c("blue", "red"), lty=1, lwd=3)

}

abline(1,0, col = "gray", lty=3, lwd=1)

box(bty = "n")

}

################################The END######################################
